# RIPK3 promoter hypermethylation in hepatocytes protects from bile acid-induced inflammation and necroptosis

**DOI:** 10.1038/s41419-023-05794-0

**Published:** 2023-04-18

**Authors:** Jessica Hoff, Ling Xiong, Tobias Kammann, Sophie Neugebauer, Julia M. Micheel, Nikolaus Gaßler, Michael Bauer, Adrian T. Press

**Affiliations:** 1 Department of Anesthesiology and Intensive Care Medicine, Nanophysiology Group, Jena University Hospital, Jena, 07747 Germany; 2 Center for Sepsis Control and Care, Jena University Hospital, Jena, 07743 Germany; 3 Department of Clinical Chemistry and Laboratory Diagnostics, Jena University Hospital, Jena, 07747 Germany; 4 Pathology, Jena University Hospital, Jena, 07747 Germany; 5 Faculty of Medicine, Friedrich Schiller University Jena, Jena, 07747 Germany

**Keywords:** Necroptosis, Cell signalling

## Abstract

Necroptosis facilitates cell death in a controlled manner and is employed by many cell types following injury. It plays a significant role in various liver diseases, albeit the cell-type-specific regulation of necroptosis in the liver and especially hepatocytes, has not yet been conceptualized. We demonstrate that DNA methylation suppresses RIPK3 expression in human hepatocytes and HepG2 cells. In diseases leading to cholestasis, the RIPK3 expression is induced in mice and humans in a cell-type-specific manner. Overexpression of RIPK3 in HepG2 cells leads to RIPK3 activation by phosphorylation and cell death, further modulated by different bile acids. Additionally, bile acids and RIPK3 activation further facilitate JNK phosphorylation, IL-8 expression, and its release. This suggests that hepatocytes suppress RIPK3 expression to protect themselves from necroptosis and cytokine release induced by bile acid and RIPK3. In chronic liver diseases associated with cholestasis, induction of RIPK3 expression may be an early event signaling danger and repair through releasing IL-8.

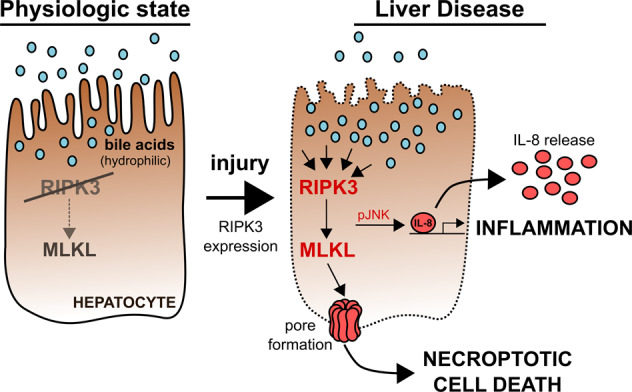

## Introduction

Regulated cell death signaling events, e.g., apoptosis, ferroptosis, necroptosis, or pyroptosis, are essential for maintaining tissue homeostasis. In addition, these pathways are involved in the induction and modulation of the immune response [1, 2] and tissue regeneration [3, 4], where each pathway has its specific regulatory mechanism and molecular components [5].

Necroptosis is a pro-inflammatory cell death type that signals damage and repair to many cell types following injury [6, 7]. Mechanistically, the formation of the necroptosis-defining necrosome requires phosphorylation of receptor-interacting serine/threonine-protein kinase 3 (RIPK3) [8] and activation of mixed lineage kinase domain-like protein (MLKL) leading to pore formation and cell death [9–11]. The first steps in the induction of necroptosis are shared with the apoptotic pathway. It has been reported that receptor-interacting serine/threonine-protein kinase 1 (RIPK1) is recruited to an active cytokine receptor, such as tumor necrosis factor receptor type-1 (TNFR1), where it is phosphorylated (pRIPK1) in a multiprotein complex, also known as complex I [12, 13]. pRIPK1 interacts with Fas-associated death domain protein to activate caspase 8 (CASP8) inducing apoptosis. Unlike apoptosis, the first step of necroptosis involves the phosphorylation of RIPK3 with concomitant CASP8 inhibition [14]. Phosphorylated RIPK3 is associated with a multiprotein complex with MLKL, resulting in its phosphorylation (pMLKL) Table 1. The pRIPK3/pMLKL complex is recruited to a membrane where multiple pMLKL proteins oligomerize into a pore, ultimately leading to cell lysis [15–17].

**Table 1:** Sequences of 8 representative CpG sites in the methylation analysis.

CpG sites	Genomic target sequence	Bisulfite converted target sequence
**Set 1**	**cg**gcaggtgctcaggaaa**cg**ctagtcaaggagaaaggca	**yg**gcaggtgctcaggaaa**yg**ctagtcaaggagaaaggca
**Set 2**	cttt**cg**ctctgccccctgccccACCCCAGGGG **CG**GGACTGTAGAGGC**GC**CTATAAGGGAAG	cttt**yg**ctctgccccctgccccACCCCAGGGG **YG**GGACTGTAGAGG**YG**TTTATAAGGGAAG
**Set 3**	GTTCAGTCAACT**CG**GAAAAAGGGTAACAACC**CG**GAAAGTAGACTCAC**CG**TCTTGGTCTAGAGACTGACCCCTGCACAGA	GTTCAGTCAACT**YG**GAAAAAGGGTAAgCAACC**YG**GAAAGTAGACTCAC**YG**TCTTGGTCTAGAGACTGACCCCTGCACAGA

Lowercase letters represent the sequence upstream of the 5′ UTR region, and capital letters the sequence in the 5′ UTR region. a/A: adenine; c/C: cytosine; g/G: guanine; t/T: thymine; y/Y: determining nucleotide after methylation.

Necroptosis is a promising target for future tumor therapeutics [18]. However, its role in the liver is controversial [19–24]. Studies have cast doubt on hepatocyte RIPK3 expression under physiological conditions [25]. By contrast, hepatocyte injury was reduced in RIPK3 knockouts in varied murine models of acute and chronic liver injury (ethanol-induced hepatitis [20], methionine-and-choline-induced fatty liver disease [23]), albeit no protection was seen against damage induced by acetaminophen [26, 27], early ischemia reperfusion [28, 29] or concanavalin-A [30, 31] (Supplementary Table S1).

Also, necroptosis is emerging as a critical mechanism in the pathogenesis of cholestasis. In cholestatic liver disease, bile acids are retained, and the bile flow is disrupted [32]. In cholestasis, the bile acid profile in serum is shifted to the glycine-/ taurine-conjugated primary bile acids CA and CDCA [33–35].

Bile acids are abundant metabolites in hepatocytes involved in developing different liver diseases [36, 37]. Altogether, 15 bile acids are detected in humans. Their formation is the primary pathway of cholesterol catabolism tightly regulated within the liver parenchyma to prevent the cytotoxic accumulation of bile acids [38, 39]. Cholic acid (CA) and chenodeoxycholic acid (CDCA) are primary bile acids, described as the dominant but not exclusive forms found in the liver. Hepatocytes conjugate CA and CDCA to taurine or glycine during biotransformation before being secreted into the bile [39]. In the colon, bile acids are subjected to various microbial-mediated transformations, including deconjugation and transformation of primary to secondary bile acids (lithocholic acid (LCA) and ursodeoxycholic acid (UDCA)) [40]. Based on the structural formation, bile acids are classified by their hydrophobicity (hydrophobic: LCA > CDCA > CA > UDCA) [41]. The hydrophobic bile acids (LCA, CDCA) are toxic and potent inducers of apoptotic or necrotic cell death. In contrast, hydrophilic bile acids are often described as cytoprotective [42–44].

Besides the well-known function of bile acids as detergents in the digestive tract and signaling under physiologic conditions, they are also highly active signaling molecules for eukaryotic cells in supraphysiological concentrations as they occur in various liver diseases. Furthermore, the importance of bile acids in regulating inflammation has been highlighted, for instance, in their ability to trigger inflammation and cell death [45, 46]. The presence of pathologically increased bile acid concentrations in hepatocytes, by accumulation, induces different cell death mechanisms (e.g., apoptosis, necrosis, or necroptosis) [44]. This fact implies that hepatocytes must have acquired an endogenous mechanism to counteract bile acids’ pro-inflammatory and cell-toxic properties, e.g., due to the loss of critical mediators of inflammation and cell death.

Here, we investigate the expression profile of hepatocellular RIPK3 protein, responsible for the induction of necroptosis, under physiological and pathological liver conditions. Further, we investigate RIPK3 regulatory mechanisms using bile acids in vitro to prevent and trigger hepatocellular inflammation and tissue regeneration.

## Materials and methods

### Cell isolation and culture

HepG2 cells were cultured in Dulbecco’s Modified Eagle Medium containing F12 nutrient mix (DMEM:F12; Biozym) supplemented with 10% fetal calf serum (FCS) and 100 iU penicillin and 100 iU streptomycin at 37 °C in a humidified atmosphere of 5% CO_2_. Before the day of the experiment, cells were washed with phosphate-buffered saline without calcium and magnesium (PBS) and cultured into either 6-, 12- or 96-well tissue culture plates depending on the experimental conditions.

Plateable, cryopreserved primary human hepatocytes (pHep) from one male donor (45 years, BMI 24.2) and donor pools (20 male or 20 female) were purchased from Lonza, Switzerland. The characterization of the hepatocytes from single male donors and donor pools is provided in Supplementary Table S2. According to the manufacturer’s instructions, cells were thawed and cultured using Lonza’s recommended hepatocyte culture media. Hepatocytes were seeded at 2 × 10^6^ cells per well in a 6-well plate coated with collagen type I (Corning) at 10 µg cm^−2^.

**Fig. 1:**
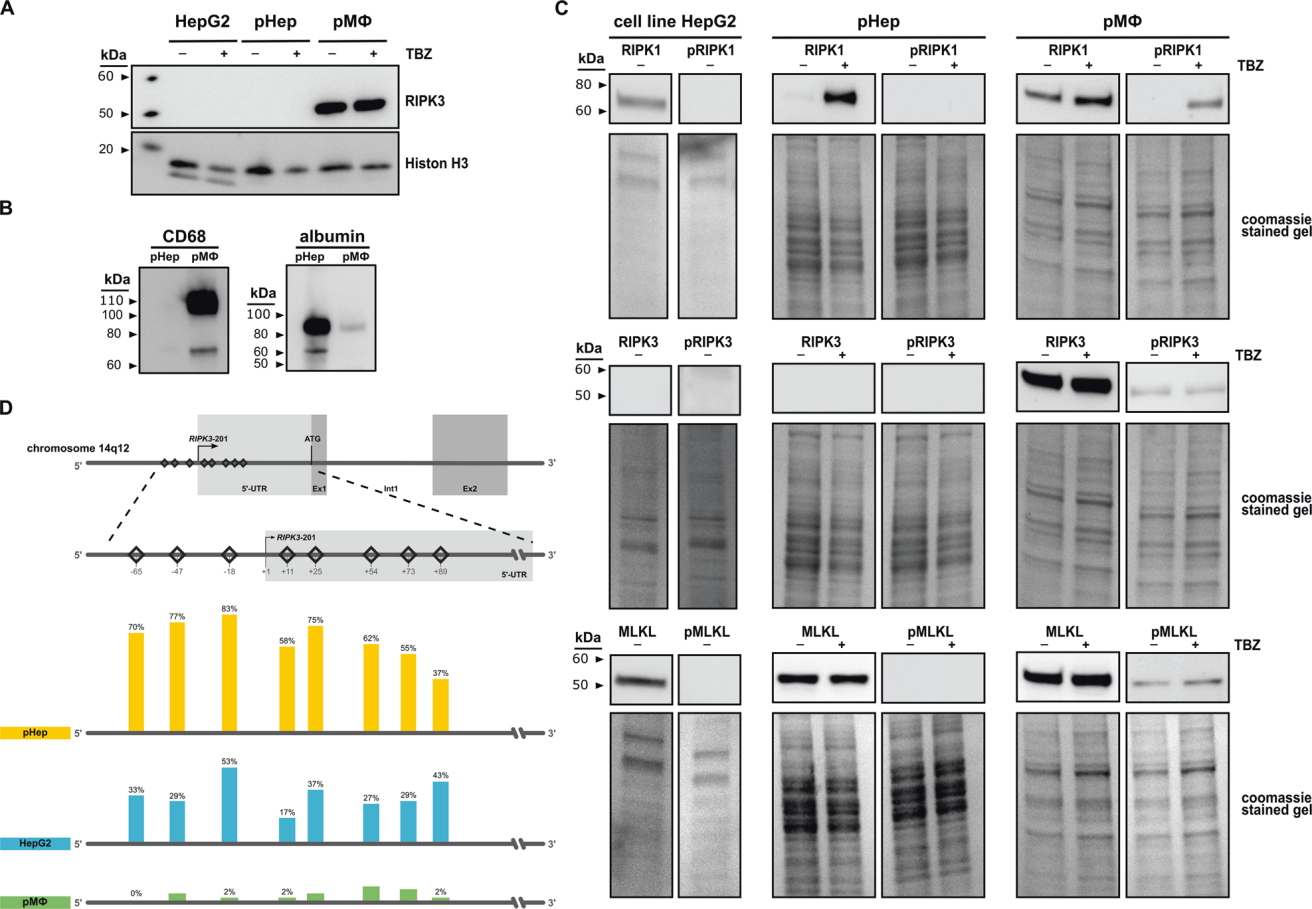
Necroptosis signaling is absent in primary human hepatocytes under physiological conditions. **A** Protein expression of RIPK3 in HepG2 cells, primary human hepatocytes (pHep), and primary human macrophages (pMФ). (+) indicates TBZ stimulation with necroptosis-inducing reagents where cells were pre-incubated for 0.5 h with BV6 (5 µmol L^−1^) and zVAD-fmk (20 µmol L^−1^) before stimulation with TNF-α (20 ng mL^−1^) for 6 h. Histone H3 and coomassie-stained gels were used to control the loading. **B** Protein expression of molecules with high abundance in hepatocytes (albumin) or macrophages (CD68) are detected to assess the purity of the pHep and pMФ. **C** Protein expression of molecules in the necroptosis signaling pathway detected in HepG2 cells, pHep, and pMФ. (+) indicates TBZ stimulation with necroptosis-inducing reagents where cells were pre-incubated for 0.5 h with BV6 (5 µmol L^−1^) and zVAD-fmk (20 µmol L^−1^) before stimulation with TNF-α (20 ng mL^−1^) for 6 h. Coomassie staining of SDS-PAGE gel was used as a loading control. *n* = 2–5. **D** Pyrosequencing detected RIPK3 promoter methylation analysis in pMФ, pHep, and the hepatocellular carcinoma cell line (HepG2). Bars and numbers depict the mean percentage of methylated cytosine in the different promoter regions of each sample. Sequencing was performed on 1 (pHep, pMФ) or 2 (HepG2 cells) individual samples, each containing DNA from 1 million cells. Statistics: Figure Data, additional statistical information, and *p* values are provided in Supplementary Materials 2 and 3.

According to the manufacturer’s protocol, primary human macrophages (pMФ) were isolated from the whole blood of healthy volunteers with Biocoll separating solution (Merck) and seeded at a density of 2 × 10^6^ cells per well into a 6-well plate. After 5 days of differentiation by cultivating in Dulbecco’s Modified Eagle Medium (DMEM; Lonza) supplemented with 10% FCS, 10 ng mL^−1^ M-CSF (ReproTech), and 10 µg mL^−1^ ciprofloxacin (Fresenius Kabi), cells were washed with PBS and subsequently used. Purification of the primary cells was characterized by specific markers (Fig. 1A).

### Transfection

HepG2 cells were transfected for 24 h with 1 µg purified plasmid DNA of pcDNA-FLAG or pcDNA3-FLAG-RIPK3 using Lipofectamine 3000 (ThermoFisher Scientific) as a transfection reagent in serum-reduced Opti-MEM medium (ThermoFisher Scientific) according to the manufacturer’s protocol. The pcDNA-FLAG plasmid was purchased from ThermoFisher Scientific. pcDNA3-FLAG-RIPK3 (Addgene plasmid, http://n2t.net/addgene:78815; RRID: Addgene_78815) was a gift from Jaewhan Song [47].

### Stimulation with necroptosis-mix

Cells were washed with PBS and pre-treated with 5 µmol L^−1^ inhibitor of apoptosis protein antagonist BV6 (APExBIO) and 20 µmol L^−1^ pan-caspase-inhibitor zVAD-fmk (Bachem) for 30 min before stimulation with 20 ng mL^−1^ tumor necrosis factor-α (TNF-α) (Prospec Inc) in cell culture media.

### Stimulation with bile acids

After transfection with pcDNA-FLAG and pcDNA3FLAG-RIPK3 for 24 h, HepG2 cells were carefully washed with PBS and stimulated with DMSO-diluted bile acids ([G/T]CA, [G/T]CDCA, [G/T]UDCA, TLCA: 50 µmol L^−1^; LCA: 5 µmol L^−1^; GLCA: 10 µmol L^−1^) in DMEM:F12 medium (without supplements).

### Mice

Male and female FVB/N(Rj) mice at 8–12 weeks of age were used for all experiments. FVB/N(Rj) mice were partially bred within the Jena University Hospital animal facility under specific pathogen-free conditions and purchased from Janvier Labs. The animals had access to conventional rodent chow and water ad libitum. They were maintained under a constant humidity (50–60%), a 12-h light/dark cycle (incl. 20 min dim phases), and a constant temperature (24 °C). All experimental protocols were approved by the ethics committee and local government authorities in Thuringia, Germany (UKJ-19-010).

### Experimental mouse models

#### Surgical animal models

Surgery was performed under anesthesia inhalation (1–2% Isoflurane, CP-Pharma, and 100 mL min^−1^ carbogen) and 1 mg kg^−1^ body weight p.o. Meloxicam (0.5 mg mL^−1^ suspension, CP-Pharma) was given 1 h before surgery for pain relief. A midline incision was used to open the abdomen and expose the bile duct and liver. After the surgical procedure (details for different models are given below), the abdominal layers were closed with 4-0 antibacterial sutures (Ethicon) and Bupivacaine (2–4 mg kg^−1^ body weight, PUREN Pharma) was administered intra-incisional for topical anesthesia. In addition, the animals received Ringer acetate (20 mL kg^−1^, Berlin Chemie AG) subcutaneously for fluid resuscitation and were offered an additional heat source (warm lamp) during the recovery phase. Regular and soaked food was available on the ground for the animals at all times after surgery. The animals were scored and weighed a minimum of twice daily. The scores were designed to reflect post-surgical conditions and specific symptoms of surgical intervention. Supplementary Table S3 provides additional information on the model regarding the change in body weight, ALAT, and ASAT can be found in.

##### General surgery

This group was used as a sham group. The abdominal layers were closed after liver and bile duct exposure without additional intervention.

##### Bile duct ligation (BDL)

Intrasurgical, the bile duct was ligated twice with two 6-0 braided silk sutures (Teleflex Medical).

##### Warm ischemia-reperfusion injury (IR)

Intrasurgical, a microvascular clamp was placed above the left lateral branch of the portal vein to interrupt the blood flow to the left lateral lobe. The liver was covered and kept moist with Ringer acetate while the body temperature was maintained at 37 °C with a heating plate. After 60 min of partial hepatic ischemia, the clamp was removed to initiate the reperfusion for 23 h.

#### Non-surgical animal models

##### Acetaminophen-induced liver injury (APAP)

Animals were fastened for 16 h before APAP injection to reduce the liver’s metabolic activity and glutathione levels. Acetaminophen (Sigma Aldrich) was dissolved in pre-warmed PBS and mice were treated with 300 mg kg^−1^ body weight intraperitoneally [48]. After the APAP injection, mice had free access to food and water. Sham animals received an intraperitoneal injection of PBS.

##### Peritoneal contamination and infection (PCI)

Human feces (60–70 µL feces suspension) diluted in Ringer acetate (Berlin Chemie AG) (PCI group) or Ringer acetate alone (sham group) was injected intraperitoneally. The bacterial composition of the human feces suspension, mainly *Escherichia coli* (2.5 × 10^8^ CFU/mL), *Actinobacteria* (2.5 × 10^8^ CFU/mL), and *Bifidobacteria* (2.9 × 10^8^ CFU/mL), used in this study, had been characterized previously [49]. Additionally, 2.5 mg of metamizole (Novaminsulfon-Ratiopharm, Ratiopharm) was administered every 6 h on the animal’s tongue. Twice daily, animals received 25 mg kg^−1^ body weight meropenem (Inresa Pharmaceutical, 2.5 mg mL^−1^ diluted in Ringer acetate) subcutaneously.

### Hepatocyte isolation

Animals subjected to surgical or non-surgical models of liver injury were sacrificed after defined time points by an overdose of ketamine (>300 mg kg^−1^ body weight, CP-Pharma) and xylazine (>50 mg kg^−1^ body weight, Bayer). For hepatocyte isolation, liver perfusion was performed. The system comprises a peristaltic pump with adjustable speed, silicone tubing immersed in different buffers in the water bath (38 °C), and a cannula (we used 25 G) at the tube outlet. The pump speed was set to a maximum of 5 mL min^−1^ (beginning) and was increased to 12 mL min^−1^ for perfusion after the portal vein cannulation. The perfusion was started using Krebs Henseleit Buffer (KHB, Biochrom) supplemented with 8 U mL^−1^ heparin (Heparin-Natrium-2500-Ratiopharm, stock solution: 5000 I.E. mL^−1^, Ratiopharm). When the liver appeared pale, the perfusion medium was changed to Liver Digest Medium (LDM, ThermoFisher Scientific). Perfusion was maintained until the liver appeared digested. Livers were separated, kept in a cell culture dish with some LDM after perfusion, and incubated for another 5 min at 37 °C. The tissue was strained through a 70 µm nylon cell strainer (Corning) into a conical tube using approximately 30 mL of DMEM (ThermoFisher Scientific). Hepatocytes were isolated and purified by centrifugation thrice for 4 min at 40 × *g* and 4 °C. The supernatant was removed and replaced with 30–40 mL DMEM to wash the hepatocyte pellet between each centrifugation step. Aliquots and lysates were prepared after counting hepatocytes in 10 mL DMEM.

### Methylation analysis

Quantitative DNA methylation analysis from purified primary human hepatocytes employing methylation samples was obtained by methylation-sensitive Pyrosequencing (EpigenDX) and Illumina sequencing (ZymoResearch). Monocytes (Pyrosequencing) and non-parenchymal cells (NPC) (Illumina sequencing) served as reference material. Pyrosequencing was performed on single individual hepatocyte donors. Illumina sequencing was performed on hepatocyte donor pools (20 male and 20 female). All human hepatocytes and NPC were obtained from Lonza (Switzerland). The monocytes were kindly provided by Dr. Ghait (Universitätsklinikum Jena).

The DNA-extraction, bisulfite conversion, sequencing, and bioinformatics data are detailed in the Supplementary information methods section ‘Methylation Analysis by Pyrosequencing’ and ‘Methylation Analysis by Illumina sequencing’.

### Western blot

After stimulation, cells were washed with PBS before lysis in RIPA buffer (150 mmol L^−1^ NaCl, 1 mmol L^−1^ EDTA, 0.1% SDS, 1% Triton X-100, 500 mmol L^−1^ Tris-HCl, 0.5% deoxycholic acid) containing inhibitors of phosphatases (1:10 PhosphoStop; Roche) and proteases (1:100 Protease Inhibitor Cocktail; ThermoFisher Scientific). After determination of the protein concentration by BCA Protein Assay Macro Kit (Serva Gelelectrophoresis GmbH), 10 µg of cell extracts were loaded and separated by SDS-PAGE, transferred to 0.2 µm PVDF membranes (Carl Roth), blocked with 5% bovine serum albumin (BSA) or nonfat dry milk in TBS-T and incubated with monoclonal antibody rabbit IgG (in 5% BSA/ nonfat dry milk in TBS-T) overnight at 4 °C. All antibodies were purchased from Cell Signaling Technology targeting either RIPK1 (#3493, 1:750), pRIPK1 (#65746, 1:750), RIPK3 (#13526, 1:750), pRIPK3 (#93654, 1:750), RIPK3 (#95702, 1:750), MLKL (#14993, 1:750), pMLKL (#91689, 1:750), JNK (#9258, 1:1000), pJNK (#4668, 1:1000), albumin (#4929, 1:1000), CD68 (#86985, 1:1000) or MCAM (#68706, 1:1000). The RIPK3 antibody shows a specific signal at around 55 kDa, matching the theoretical molecular weight between 46 to 62 kDa. Besides the Coomassie staining of SDS-PAGE gel, a Histone H3 (Cell Signaling Technology, #4499, 1:2000) antibody was used for loading control. Membranes were washed with TBS-T and incubated with HRP-conjugated goat anti-rabbit IgG antibody (1:2000 in 5% nonfat dry milk in TBS-T; Jackson ImmunoResearch) at room temperature for 1 h. For visualization, the chemiluminescence method was performed with standard and ultra-sensitivity substrates (SERVA*Light* Eos CL HRP WB Substrate Kit; SERVA*Light* EosUltra CL HRP WB Substrate Kit; Serva Gelelectrophoresis GmbH). Raw data files of all western blots are depicted in the supplementary information.

### RIPK3 staining of human samples

To investigate the relationship between RIPK3 expression and cholestasis, patients suffering from various liver diseases (Supplementary Table S4) were classified according to the bilirubin plasma concentration into two groups, no cholestasis (<21 µmol L^−1^) and cholestasis (≥21 µmol L^−1^). Paraffin-embedded liver tissue sections (4 µm thickness) were mounted on glass slides. For immunostaining, tissue sections were deparaffinized and permeabilized by citrate buffer (pH 6) for 25 min (Dako). Slices were blocked with 5% donkey serum (37 °C, 30 min) and then incubated with a primary antibody targeting RIPK3 (#95702, Cell Signaling Technology) at 4 °C overnight diluted in antibody diluent (Dako). Negative controls were incubated with antibody diluent only. Slides were then incubated with Alexa-Fluor 568-conjugated anti-rabbit IgG (1:500, ThermoFisher Scientific) and 5 U mL^−1^ DY-636 conjugated Phalloidin (Dyomics) for 60 min at room temperature. Finally, the sections were mounted with Roti-Mount FluorCare mounting media (Carl Roth).

Images were acquired on a laser scanning microscope (LSM-780, Carl Zeiss, Germany) at ×400 magnification (×40, numeric aperture 0.95, Carl Zeiss, Germany) and 4.82 pixels per micron. Alexa-Fluor 568 fluorescence was excited at 561 nm and emitted light between 597–630 nm was recorded. To visualize the tissue structure, autofluorescence was detected at 530–595 nm by excitation of the specimen at 488 nm. Images were further analyzed using the FIJI distribution of ImageJ v 1.51 (Freeware, NIH, USA). The representative pictures were taken with a 63× objective (NA: 1.40 oil). Ten different regions per image were analyzed (40× objective, NA: 0.95), and the mean fluorescence intensity was measured to quantify RIPK3 expression in hepatocytes. A specific threshold was set for all the images, selecting signals visible from the endothelium around the hepatocyte to evaluate RIPK3 expression in endothelial cells. The mean signal intensity of the negative staining control (background) in both groups was subtracted.

### Quantification of bile acids by mass spectrometry

A LC–MS/MS in-house assay concentration of 15 bile acids was determined in HepG2 cells and isolated primary murine hepatocytes with two sample preparations. First, threefold (w/v) ethanol-phosphate buffer (15% 0.01 mol L^−1^ phosphate buffer solution pH 7.5, 85% ethanol) was added to pre-weighed isolated primary murine hepatocytes. Samples were homogenized in a pebble mill (QiaShredder) and centrifuged at 16000 g for 5 min. The supernatant was used for bile acid quantification. Second, 1 × 10^6^ HepG2 cells were seeded and incubated for two days. Afterward, cells were washed twice with 500 µL PBS, trypsinized with 100 µL 0.05% trypsin-EDTA for 3 min at 37 °C and 5% CO_2,_ and incubated with 1 mL DMEM to stop the trypsin reaction. Next, the cell suspension was centrifuged for 3 min at 4 °C and 260 × *g*. Afterward, the cell pellet was washed by resuspending them twice in 500 µL 4 °C cold PBS and centrifuged for 3 min at 4 °C and 260 × *g*. The washed pellet was resuspended in 100 µL PBS and homogenized in a pebble mill (QiaShredder). After centrifugation for 5 min at 4 °C and 13,000 × *g*, the supernatant was used for bile acid quantification. The sample preparation was then followed by protein precipitation and filtration of the samples. For quantification, an Agilent 1200 high-performance liquid chromatography system (Agilent Technologies GmbH, Germany) with a CTC-PAL autosampler coupled to an API 4000 Triple Quadrupole mass spectrometer with electrospray ionization source (AB Sciex, Germany) was used. All chromatographic separations were performed with a reverse-phase analytical column. The mobile phase consisted of water and methanol containing formic acid and ammonia acetate at a total flow rate of 300 µL min^−1^.

### Site-directed mutagenesis

The Q5 Site-Directed Mutagenesis PCR reaction was performed on 20 ng of the RIPK3 vector according to the supplier’s instruction (New England Biolabs) using primers and annealing temperatures (*T*_m_) in Supplementary Table S5. The PCR product was mixed with the provided kinase and ligase DpnI mix and transformed into NEB 5-alpha competent *E. coli*. The transformed *E. coli* were spread onto LB agar plates containing ampicillin (100 µg mL^−1^). Isolated colonies were expanded into overnight cultures and the pDNA was isolated with the ZymoPURE Plasmid MiniPrep according to manufacturer’s protocol (Zymo Research). The mutation in the sequence was confirmed by sequencing (Macrogen).

### Live-cell imaging of cell death

HepG2 cells were seeded, transfected, and stimulated as described in sections ‘Cell isolation and culture’, ‘Transfection’, and ‘Stimulation’ with bile acids’.

For live-cell imaging Propidium Iodide (PI) (Carl Roth, Germany), dissolved in deionized water (2 mg ml-1 stock), was added to the cells in serum-free medium (DMEM:F12; Biozym, Germany) (PI final: 2 µg mL^−1^). Cells were imaged after at least 5 min of incubation without washing using a LSM-780 (Carl Zeiss, Jena, Germany) equipped with an incubation chamber set to 37 °C, 5% CO_2,_ and a 20× plan-apochromatic objective with a numeric aperture of 0.8 (Carl Zeiss, Jena, Germany). H33342 was illuminated at 405 nm (diode laser) and PI at 561 nm (diode-pumped solid-state laser). Fluorescence was captured on a photomultiplier tube through a 410 and 499 nm bandpass filter (H33342) and a GaAsP detector using 593 nm and 712 nm bandpass (PI). Images were recorded at 16-bit with a pixel dwell time of 1.58 µs and a pixel size of 0.42 µm × 0.42 µm.

The data were analyzed using FIJI distribution of ImageJ v 1.53c applying a previously developed Fluorescent Nuclei Measurement Macro [50]. Before performing the macro, all images applied background subtraction (50 px, rolling ball radius). Then, data from all images were combined in tables and PI-positive nuclei fractions were computed in R GNU (v 4.0.5) using R-Studio (v 1.4.1103).

### Calf intestinal phosphatase assay

20 µg protein lysate was incubated in calf intestinal phosphatase (CIP)-buffer (100 mmol L^−1^ NaCl, 50 mmol L^−1^ Tris-HCl, 10 mmol L^−1^ MgCl_2_, 1 mmol L^−1^ DTT, an inhibitor of protease) containing 1 U CIP per µg protein for 1 h at 37 °C. Afterward, a western blot was performed with 4 µg protein, as indicated in the ‘Western Blot’ section.

### Lambda phosphatase assay

Twenty micrograms protein lysate was incubated with lambda phosphatase according to the manufacturer’s protocol (New England Biolabs). Afterward, a western blot was performed with 15 µg protein, as indicated in the ‘Western Blot’ section.

### IL-8 enzyme-linked immunoassay

Transfection was performed as described in paragraph ‘Transfection’ and stimulation as described in paragraph’ Stimulation with Bile Acids’. HepG2 cells were washed with PBS, lysed in the RIPA buffer and the protein concentration was measured with the BCA Protein Assay Macro Kit according to the manufacturer’s protocol (Serva Gelelectrophoresis GmbH). One hundred microliters of the total lysate was used for the IL-8 enzyme-linked immunosorbent assay (ELISA). The assay was performed using human IL-8 ELISA Max Deluxe (BioLegend) following manufacturer’s instructions. Absorbance was measured at 450 and 570 nm using a plate reader (EnSpire, Perkin Elmer).

### Quantitative PCR

Transfection and stimulation were performed as described in paragraphs’ Transfection ‘and ‘Stimulation with Bile Acids’. RNA isolation was performed with the Direct-zol RNA MicroPrep Kit according to manufacturer’s protocol (Zymo Research). RNA concentration and purity were assessed spectrophotometrically on NanoDrop 2000c (ThermoFisher Scientific). For analysis of IL-6, IL-8, IL-33, and HMGB1, cDNA was generated using the RevertAid First Strand cDNA Synthesis Kit according to the manufacturer’s protocol (ThermoFisher Scientific). A quantitative PCR was set up with a 25 ng cDNA template, 0.5 µmol L^−1^ of each, forward and reverse primer for the individual genes of interest (Eurofins Genomics, Germany) and GoTaq qPCR 2× Master Mix (Promega, Germany). The qPCR was carried out on a RotorGene Q (Qiagen) using the following temperature protocol: 95 °C for 2 min, followed by 40 cycles at 95 °C for 15 s, 60 °C for 60 s, and 70 °C for 30 s. A final ramp from 70 to 95 °C for around 90 s was set to determine melting curves.

The mRNA in each sample was normalized to the sample-specific Ct-value of the housekeeping gene HPRT. Then, fold induction of gene expression was calculated using the Pfaffl method [51]. Primers used are found in Supplementary Table S5.

### Statistical analysis

The statistical analysis was conducted in SigmaPlot 13.0 (Systat Software, USA). The results are depicted as mean values with a standard error if not otherwise stated. In addition, information about replicates and significance tests is provided within the figure legend.

Before each significance test, the data were examined for normality (Shapiro–Wilk test) and equal distribution (Brown–Forsyth test). Afterward, corresponding parametric or non-parametric tests statistically evaluated the data. The applied test methods are provided in the figure legends. The figure data are provided in Supplementary Information 2. The complete statistical evaluation, including test justifications, the results of Shapiro-Wilk and Brown–Forsyth tests and *p* values, is provided figure-wise in Supplementary Information 3.

## Results

### RIPK3 promoter methylation protects hepatocytes from necroptosis under physiological conditions

RIPK3 was not detected in HepG2 cells, a hepatocellular carcinoma cell line, and primary human hepatocytes (pHep) (Figs. 1A and S1A). Primary human macrophages (pMФ), which served as a positive control for RIPK3 expression [27, 52–54] validated the antibodies by their high levels of expressed RIPK3 (Fig. 1A).

A western blot against CD68 and albumin was performed from the same lysates to analyze the purity of the isolated primary hepatocytes and macrophages. CD68, a protein highly expressed by monocytes and macrophages, served as a macrophage-specific marker, whereas albumin, which has great abundance in hepatocytes, was used to characterize hepatocytes. We did not detect CD68 expression in the hepatocyte lysate, indicating the high purity of the samples (Fig. 1B). However, a low albumin expression was found in macrophage samples (Fig. 1B). This expression may be attributed to the cultivation medium that is bound to the macrophages despite washing steps or due to low expressed (advanced glycation end-product)-albumin in macrophages [55].

While RIPK3 expression was absent in hepatocytes, RIPK1 and MLKL were highly expressed in three cell types HepG2, pHep, and the positive control pMФ (Fig. 1C, Supplementary Table S6). Next, we were interested in whether an inflammatory molecule could induce RIPK3 expression. We selected the previously investigated inflammatory, necroptosis-activating mix of TNF-α, the pan-caspase inhibitor zVAD-fmk, and an inhibitor of the apoptosis protein (IAP) family (TBZ-mix). This mix was previously established to induce, depending on the stimulation duration, the expression, and the phosphorylation of the necroptotic proteins RIPK1, RIPK3, and MLKL in pMФ [56, 57]. In our model, stimulating pHep for 6 h with TBZ-mix increases the expression of RIPK1. A slight increase in pRIPK1 levels are notable but did not become significant (Figs. 1C and S1B). RIPK3 and pRIPK3 were again not detected in the hepatocytes, while MLKL expression remained similar (Fig. 1C). Interestingly, TBZ increases the pMLKL signal significantly in pHep (Fig. S1B). TBZ in pMФ induced significant phosphorylation of RIPK1 after 6 h. At the chosen time-point, no effect on RIPK3 or MLKL phosphorylation was observed (Fig. S1B). Thus, the TBZ-mix activates RIPK1 in pHep and pMФ but does not induce a detectable RIPK3 expression after 6 h stimulation.

To investigate the background of this undetectable RIPK3 expression in hepatocytes, we performed a DNA methylation-specific sequencing analysis by Pyro- and Illumina Sequencing. With both methods, we independently investigated CpG islands in the human *RIPK3* promoter regions to elucidate a cause for the lack of RIPK3 expression (Fig. 1D, Supplementary Table S7). Pyrosequencing analyzed eight promoter elements located at the 5′ untranslated region (UTR) and the initiation site of transcription in the RIPK3 gene, located approximately −65 to +89 base pairs around the transcriptional start. RIPK3 promoter regions in pHep were hypermethylated with relative methylated cytosine levels ranging from 37–83% and a global methylation level overall analyzed areas of 65% (Fig. 1D). Similarly, the promoter region of the RIPK3 gene in HepG2 cells was hypermethylated. Nevertheless, with a relative cytosine methylation of 21–50% (35% globally) in HepG2 cells, the hypermethylation is less pronounced than in pHep (Fig. 1D). To strengthen this finding in pHep, we further performed methylation-specific Illumina sequencing in the same region with DNA samples from donor pools of 20 male or female patients. In addition, a human NPC liver cell sample was utilized as a positive control with RIPK3 expression. While in the NPC sample, global RIPK3 promoter methylation quantified was 13%, the methylation levels of the same region in pHep were 63% in females and 53% in males (Supplementary Table S7), confirming the methylation results obtained by pyrosequencing in pHep.

Ultimately the hypomethylating reagent 5-azacytidine (5-Aza) [58] or an LPS and cytokine mix mimicking a much stronger pro-inflammatory hepatic mileau [59] than the TBZ-mix induced RIPK3 expression in HepG2 cells within 24 h (Fig. S2).

### RIPK3 expression during acute and chronic liver injuries

The RIPK3 promoter is methylated in hepatocytes, and RIPK3 expression is silenced in naïve HepG2 cells and human primary hepatocytes. Methylation patterns, however, are highly dynamic. The experiments with 5-Aza and an LPS-cytokine mix suggest RIPK3 expression induction during inflammation (Fig. S2). Thus we further studied whether RIPK3 may be induced due to liver injury facilitating necroptosis, aggravating inflammation, and liver cell death. We utilized liver injury models previously investigated in the context of necroptosis research. We chose the bile duct ligation model (BDL), which resembles chronic cholestasis due to a post-hepatic sterile injury [60]. Further, we induced warm ischemia for 1 h followed by reperfusion resulting in solid ischemia-reperfusion (IR) liver tissue injury after 24 h. This IR injury model is suitable for investigating pericentral cell death and injury due to the generation of oxidative stress and the production of inflammatory cytokines [61]. The model mimics the pathophysiological events occurring during liver transplantation or shock [62]. The acetaminophen-induced liver injury (APAP) is an acute model of liver damage that mimics a drug-induced liver injury, a common adverse effect encountered in clinical practice [63, 64]. Lastly, the peritoneal contamination and infection model (PCI) is employed to induce bacterial sepsis, one of the leading causes of death in intensive care units. This model results in acute peritonitis associated with a strong pro-inflammatory phenotype and even systemic cytokine storm, mimicked by the LPS and cytokine mix inducing the RIPK3 expression in HepG2 cells [65, 66].

**Fig. 2:**
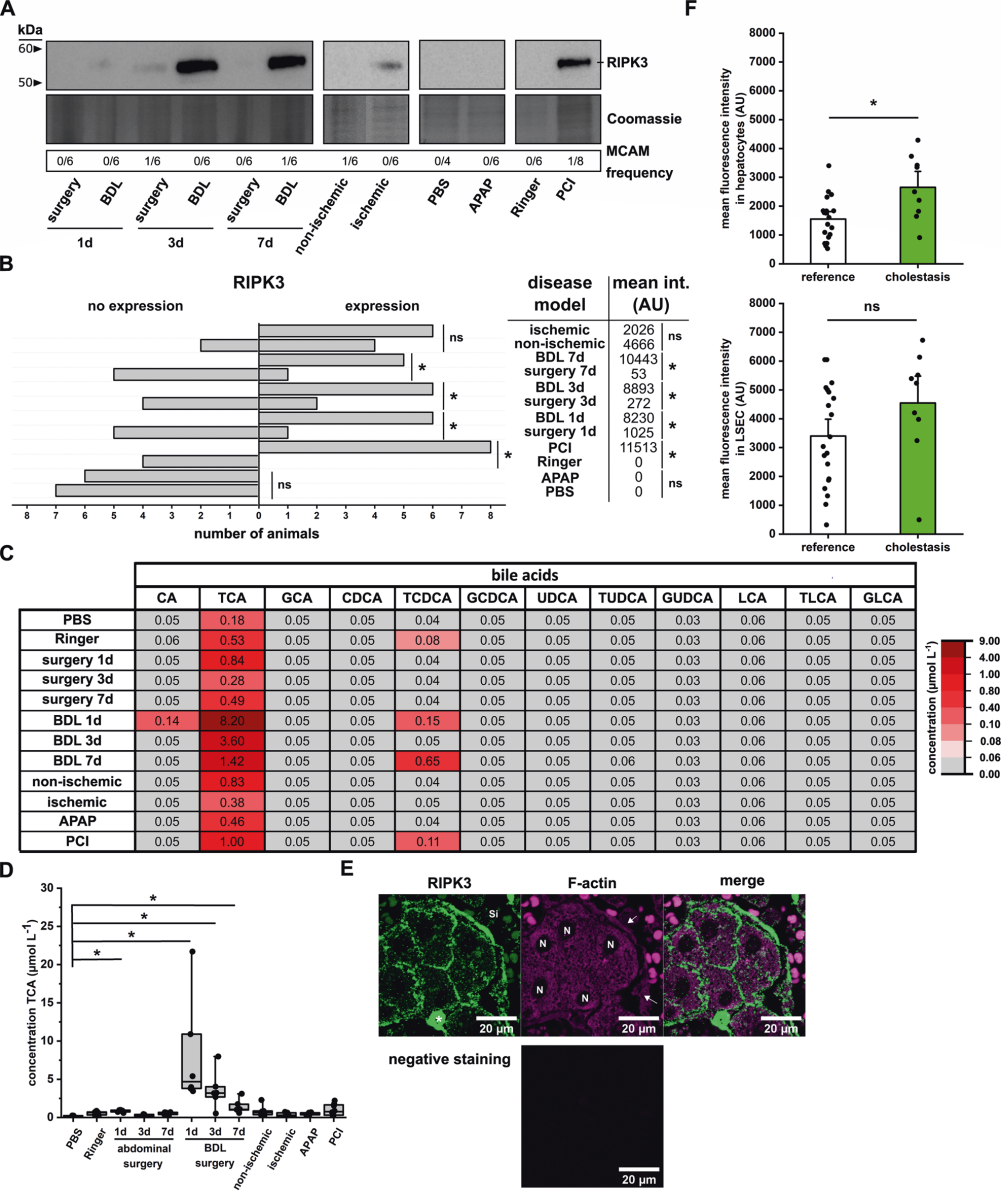
RIPK3 expression in hepatocytes is present under non-physiological conditions. **A** Representative western blot analysis of RIPK3 expression in primary murine hepatocytes isolated from different animal models of liver injury. **B** The absolute animal number expressing RIPK3 primary murine hepatocytes in different injury models and the derived mean RIPK3 signal intensity. Control groups received Ringer acetate or PBS, i.p. Animals in the abdominal surgery group get exposure to the liver and bile duct without additional intervention, whereas the BDL group has a ligated bile duct. For the ischemic animals, the left lateral branch of the portal vein was clamped to interrupt the blood flow to the left lateral lobe. Animals in the PCI and APAP group received a defined amount of human feces (PCI, 60–70 µL feces suspension) or acetaminophen (APAP, 300 mg kg^−1^ body weight) i.p. Data are presented as a relative frequency bar plot as well as the mean intensity of the signal. RIPK3 expression: *p < 0.05 vs. corresponding control group, z-test. ns not significant. Mean intensity signal: *p < 0.05 corresponding control group, unpaired *t*-test (Ringer vs. PCI: Student’s *t*-test; surgery 1d vs. BDL 1d: Welch’s *t*-test; surgery 3d/ 8d vs. BDL 3d/ 8d, PBS vs. APAP and non-ischemic vs. ischemic: Mann–Whitney test). ns: not significant. **C** Mean bile acid concentration in isolated primary murine hepatocytes detected by LC–MS/MS. The LLOQ (0.05) was assumed when a bile acid was undetected. **D** The concentration of TCA in the primary murine hepatocytes from different animal models was used. Data are presented as a median box plot (IQR: perc 25.75, median line, whiskers from minimum to maximum). *p < 0.05 vs. PBS, Kruskal–Wallis test with post-hoc Dunn’s test. **E** Representative fluorescence images of human liver sections stained for RIPK3 (green). The tissue was visualized by counterstaining F-actin with phalloidin (magenta). The negative staining control was treated and processed as the images but not incubated with primary antibodies or phalloidin. Si: sinusoid, N: nucleus, *: immune cells, arrow: liver sinusoidal endothelial cells (LSEC). **F** Image analysis of RIPK3 expression in hepatocytes and LSECs. Data are presented as mean bar plots with standard errors and individual data points. *p < 0.05 vs. control, unpaired *t*-test (LSEC: Student’s *t*-test; hepatocytes: Welch’s *t*-test). ns not significant. Statistics: Figure Data, additional statistical information, and *p* values are provided in Supplementary Materials 2 and 3.

We isolated hepatocytes from all models and analyzed the purity and expression of RIPK3. A western blot assessed the purity of each individual isolated mouse hepatocyte lysate against the melanoma cell adhesion molecule (MCAM), also known as CD146, which is highly expressed in liver sinusoidal endothelial cells (LSEC) [67–69], as well as various immune cells (e.g., lymphocytes, macrophages) [70–72]. In most samples, MCAM was not detected (Fig. S3) and only 3 out of 72 samples were contaminated with MCAM-expressing cells. The amount of samples contaminated for each group is provided as “MCAM frequency” in Fig. 2A.

In hepatocyte lysates obtained from animals 24 h after bile duct ligation (BDL group) and systemic infection (PCI), RIPK3 expression was increased significantly. RIPK3 was increased in hepatocytes from the ischemic lobe and in circa 60% of the respective non-ischemic lobes 24 h after an IR injury (Fig. 2B). In contrast, only a few animals expressed RIPK3 after sterile abdominal surgery. Further, the RIPK3 expression levels after sterile abdominal surgery were the lowest compared to the other disease models (Fig. 2B). In the acutely toxic APAP liver injury model and the control groups receiving intraperitoneal injections of sterile PBS and Ringer acetate, RIPK3 expression was not detectable in isolated hepatocytes after 24 h (Figs. 2A, B and S4). These data demonstrate that under specific pathological conditions, hepatocytes regulate RIPK3 expression and may undergo necroptosis. Since BDL and PCI, with the highest RIPK3 expression rates, are associated with a cholestatic injury, we further evaluated the bile acid composition in primary murine hepatocytes from those same animals. Taurine-conjugated primary bile acids (taurine-conjugated cholic acid (TCA) > taurine-conjugated chenodeoxycholic acid (TCDCA) > taurine-conjugated ursodeoxycholic acid (TUDCA) and taurine-conjugated lithocholic acid (TLCA)), the predominant bile acid in primary murine hepatocytes [38], were the principal accumulated bile acids in the different liver injury models. In contrast, unconjugated and glycine-conjugated bile acids were mainly below the lower limit of quantitation (Fig. 2C). The animals with bile duct ligation (BDL) showed a significant increase compared to the PBS-injected control group (Fig. 2D). Also, on the first day after sterile surgery animals showed significantly elevated TCA levels. Polymicrobial sepsis (PCI group) resulted in a notably twofold TCA elevation over the Ringer Acetate control group, which failed to get significance in the multivariate analysis. The absence of an unconjugated primary bile acid accumulation in the different injury models depicted that the hepatocellular metabolization and conjugation are functional while their elimination capacity is diminished. Thus, hepatic clearance of bile acids is decreased.

To compare whether RIPK3 expression can also be induced in patients with a cholestatic liver injury, we compared the hepatic RIPK3 expression in patients with elevated bilirubin levels, thus cholestasis. Patients that underwent liver surgery without clinically relevant elevated bilirubin levels were included in the reference group. The different underlying primary diseases are summarized in Supplementary Table S4. In addition, Alanine aminotransferase (ALAT), aspartate aminotransferase (ASAT), albumin, and C reactive protein (CRP) were assessed. Patients with a total bilirubin plasma level of ≥21 µmol L^−1^ were considered cholestatic, while patients from this cohort with plasma bilirubin of <21 µmol L^−1^ served as reference. In the control group, specimens from 13 female and 30 male patients with a mean age of 59 years (0.95 confidence interval (CI): 6.1) were included. The “cholestasis group” consisted of specimens of 4 female and 6 male patients with a mean age of 57 years (0.95 CI: 10.2). Both groups did not differ significantly regarding CRP and ALAT plasma levels. In contrast, bilirubin and ASAT levels were significantly elevated, and albumin decreased in the cholestasis group (Supplementary Table S4).

RIPK3 expression was found in hepatocytes, LSECs, and immune cells of all specimens regardless of their bilirubin level, indicating that the RIPK3 expression is induced in hepatocytes upon various liver injuries (Fig. 2E). In the hepatocytes, the RIPK3 fluorescent intensity (FI) was significantly increased the cholestasis group. A similar but not significant tendency was observed in LSECs.

Those results confirm the hepatocellular RIPK3 expression induction in mice and humans for multiple inflammatory liver diseases associated with cholestasis.

### Bile acids are sensitive to affect RIPK3 activation that triggers IL-8 release

**Fig. 3:**
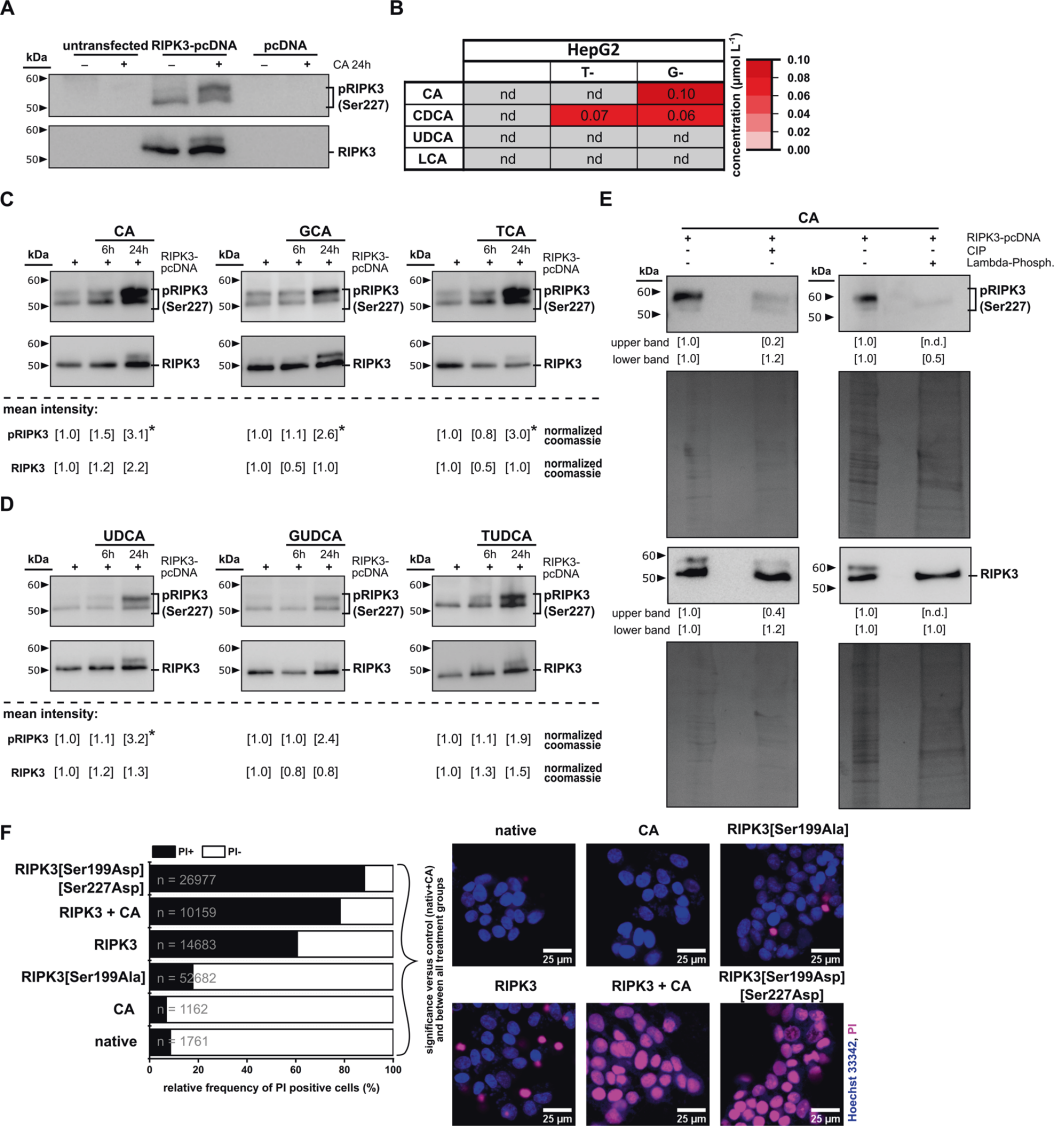
Bile acids can affect RIPK3 activation. **A** A representative blot of RIPK3 expression and phosphorylation after transfection of the hepatocellular carcinoma cell line (HepG2) with a pcDNA plasmid and RIPK3-pcDNA plasmid for 24 h. **B** Amount of bile acids in HepG2 cells detected by LC–MS/MS. The analysis was performed on 3 individual samples. Gray-colored values were not detected (nd). **C**, **D** RIPK3 phosphorylation and expression in HepG2 cells upon stimulation with different endogenous bile acids (50 µmol L^−1^) for 6 h or 24 h. Stimulation with un-, taurine (T)- or glycine (G)-conjugated **C** cholic acid (CA) and **D** ursodeoxycholic acid (UDCA). **C**, **D** Coomassie staining of SDS-PAGE gel was used as a loading control. n = 3–4. **C** *p < 0.05 vs. control, RIPK3: one-way ANOVA; pRIPK3: one-way ANOVA with post-hoc Dunnett’s test. **D** *p < 0.05 vs. control, RIPK3: UDCA with one-way ANOVA, TUDCA/ GUDCA with Kruskal–Wallis test; pRIPK3: UDCA with one-way ANOVA with post-hoc Dunnett’s test, TUDCA/ GUDCA with Kruskal–Wallis test. **E** RIPK3-pcDNA transfected HepG2 cells are lysed and treated with calf intestinal phosphatase (CIP) for 30 min at 37 °C or Lambda phosphatase for 30 min at 30 °C (400 U) before western blot analysis of RIPK3 expression and phosphorylation. The upper lane was not detected (nd) in the treated samples. Coomassie-stained gels were used as loading controls. **F** HepG2 cells were transfection with RIPK3/ RIPK3 mutants (24 h) and stimulation with cholic acid (CA) (24 h; 50 µmol L^−1^). The frequency bar depicts the percentage of PI-positive (magenta) dead cells. Representative micrographs are on the right side. Cells were counterstained with Hoechst 33342 (blue). The numbers of analyzed cells in three experiments (n) for each condition are provided in the bar plot. Z-test with Bonferroni adjusted *p* value. The *p* values of treatment groups versus control and among each other were smaller than 0.005. Statistics: Figure Data, additional statistical information, and *p* values are provided in Supplementary Materials 2 and 3.

RIPK3 expression can be triggered in HepG2 cells in vitro by inflammatory cytokines and is associated with inflammation and cholestatic liver injury in mice and humans. Since bile acids are potent signaling molecules, quickly elevating in the liver during inflammation, we investigated whether RIPK3 activation and subsequent signaling events in necroptosis might be sensitive to bile acids. Therefore, we overexpressed a previously characterized human RIPK3 (NM_006871.3) construct with an N-terminal FLAG-tag driven by a CMV promoter (RIPK3-FLAG [47]) in HepG2 cells that did not express RIPK3 endogenously (Fig. 3A). The transfection with the vector backbone alone did not lead to an expression of endogenous RIPK3 and the detected RIPK3 is entirely attributed to the RIPK3-FLAG construct in the expression vector (Fig. 3A). Further, HepG2 did not accumulate considerable amounts of bile acids under our standard cultivation conditions. Only small amounts of glycin-conjugated cholic acid (GCA) (0.1 µmol L^−1^), TCDCA, and glycine-conjugated CDCA (GCDCA) (0.07 and 0.06 µmol L^−1^) were detectable by LC–MS (Fig. 3B). Thus, to investigate the effects of the different bile acids on heterologously expressed RIPK3, we incubated HepG2 cells with bile acids at 50 µmol L^−1^ dissolved in methanol. LCA and glycine-LCA (GLCA) exhibited cell toxicity. LDH-release depicted significant membrane damage from 50 µmol L^−1^ (G)LCA incubation, quantified by the released lactate dehydrogenase activity in the supernatant. Therefore LCA and GLCA were used for stimulation at their highest non-toxic concentration (LCA: 5 µmol L^−1^, GLCA: 10 µmol L^−1^) (Fig. S5). Incubating HepG2 cells with different bile acids increased their respective intracellular concentration leaving the non-incubated bile acid concentrations unaffected (Fig. S6).

Overexpression of RIPK3-FLAG leads to a basal phosphorylation in HepG2 cells (Fig. 3A, C, D), suggesting that various endogenous mechanisms may trigger its activation already directly after expression. The unconjugated hydrophilic cholic acid (CA) and ursodeoxycholic acid (UDCA) increased RIPK3-FLAG expression after 6 and 24 h compared to untreated HepG2 cells expressing RIPK3-FLAG. Additionally, both bile acids increased RIPK3-FLAG phosphorylation after 24 h (Figs. 3C, D and S7A). Also, TCA and glycine-conjugated CA (GCA) but not UDCA metabolites significantly increased RIPK3-FLAG phosphorylation after 24 h (Figs. 3C, D and S7A). CDCA, LCA, and their metabolites did not increase RIPK3-FLAG phosphorylation (Fig. S7B, C). In contrast, TCDCA, LCA, and the LCA metabolites (TLCA, GLCA) even significantly decreased RIPK3-FLAG expression and phosphorylation levels over 24 h (Fig. S7B, C).

The western blots returned a double band for the pRIPK3. In some groups with high levels of pRIPK3, even a mass shift and double band for RIPK3 were found, which drew our attention since this phenomenon was observed but, to our knowledge, was not described before. Mutating the known RIPK3 ubiquitinylation site (lysine 5) [73, 74] did not abolish the mass shift (Fig. S8). We then evaluated whether RIPK3 hyperphosphorylation, during its activation [75] could result in the detected mass shift. Protein lysate from RIPK3-FLAG transfected, and CA-stimulated HepG2 cells were therefore incubated with calf intestinal phosphatase (CIP) or lambda phosphatase (LP), the latter being highly specific for phospho-serine, -threonine, and -tyrosine residues. CIP and LP abolished the pSer227 RIPK3-FLAG signal and diminished the mass shift observed in the total RIPK3-FLAG blot (Fig. 3E). Thus, bile acids, especially CA and UDCA, stimulated RIPK3 phosphorylation and its hyperphosphorylation, both indicating the activation of necroptosis signaling.

The CA-induced RIPK3-FLAG phosphorylation further translated into a downstream MLKL phosphorylation after 24 h, while for GLCA, significantly reducing (p)RIPK3-FLAG levels, pMLKL levels were also remarkably reduced, depicting necroptosis signaling mediated depending on the overexpressed RIPK3-FLAG activation (Fig. S9).

To fortify that bile acids trigger necroptosis in a RIPK3-dependent manner, we stimulated RIPK3-FLAG transfected HepG2 cells with CA, which potently activated RIPK3 after 24 h once more. This time we confirmed cell death and membrane damage 24 h after CA stimulation with the help of propidium iodide (PI) in a live-cell imaging experiment (Fig. 3F). In a separate experiment, we stained similarly treated HepG2 cells with a fixable fluorescent live-dead stain and an anti-pRIPK3 antibody to identify and discriminate dead (dead^+^) and pRIPK3-positive (pRIPK3^+^) cells (Fig. S10).

We further added two reference groups, where HepG2 cells expressed either a permanently activate, phosphor-positive RIPK3 (RIPK3[Ser199Asp, Ser227Asp]) or inactivated, phospho-negative RIPK3 (RIPK3[Ser199Ala]) variant [76]. The phosphor-negative variant only resulted in 18% PI^+^ and 3% dead^+^ cells, not significantly differing from naïve and CA-stimulated controls not expressing RIPK3-FLAG (below 10% PI positive (PI^+^) or dead^+^ in both groups). Thus, neither a permanently inactive RIPK3-FLAG variant nor CA drives cell death in this experiment.

In contrast, the transfection with the permanently active RIPK3[Ser199Asp, Ser227Asp] variant resulted in 88% PI^+^ HepG cells. Co-staining of the live-dead stain and pRIPK3 returned 64% dead^+^, 14% pRIPK3^+^, and 16% double-positive HepG2 cells for the active RIPK3[Ser199Asp, Ser227Asp] variant.

Transfection of HepG2 cells with the wild-type human RIPK3-FLAG increased the PI+ cells’ fraction to 61%. The fraction of dead^+^, pRIPK3^+^, and double-positive cells increased similarly significantly to a total of 52% (30% dead^+^, 21% pRIPK3^+^, and 1% double-positive). The stimulation of RIPK3-FLAG expressing HepG2 cells with CA significantly increased the fraction of PI^+^ cells further to 78%. Also, in the co-stian experiments, the fractions of dead^+^, pRIPK3^+^, and double-positive (32%, 41%, and 13%) cells increased significantly compared to the negative controls and wild-type RIPK3-FLAG transfected cells.

Those experiments confirmed that CA induces an activating RIPK3 phosphorylation, which translates into a detectable MLKL phosphorylation, increased membrane permeability and cell death.

**Fig. 4:**
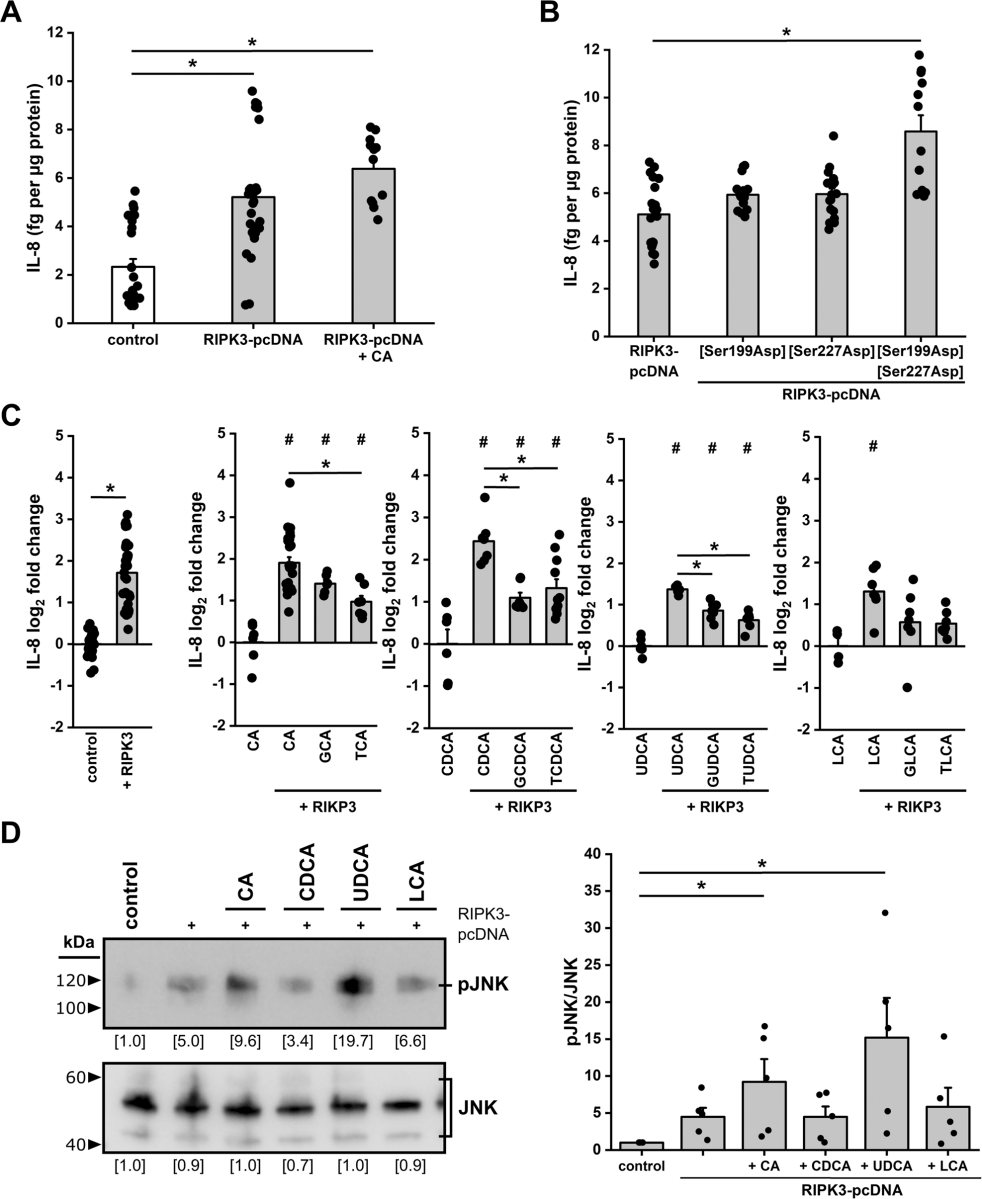
RIPK3 facilitates bile acid-mediated IL-8 release. **A** Relative secretion of the pro-inflammatory cytokine IL-8 in HepG2 cells after transfection with RIPK3-pcDNA and stimulation with CA (24 h; 50 µmol L^−1^). Data are presented as mean bar plots with standard error and individual data points. *p < 0.05 vs. control, Kruskal–Wallis test with post-hoc Dunn’s test. **B** Secretion of the pro-inflammatory cytokine IL-8 in HepG2 cells after transfection with RIPK3-pcDNA plasmid and phospho-positive mutants of RIPK3 to aspartic acid (Asp). Data are presented as mean bar plots with standard error and individual data points. *p < 0.05 vs. RIPK3-pcDNA, Kruskal–Wallis test with post-hoc Dunn’s test. **C** *IL-8* mRNA levels in HepG2 cells with and without transfection of the RIPK3-pcDNA and 24 h stimulation with different bile acids (24 h; 50 µmol L^−1^, LCA: 5 µmol L^−1^, GLCA: 10 µmol L^−1^). Data are presented as mean bar plots with standard error and individual data points. ^#^ p < 0.05 vs. bile acid treatment without RIPK3 expression and * p < 0.05 vs. indicated groups, one-way ANOVA with post-hoc Tukey test. **D** JNK expression and phosphorylation in HepG2 cells upon stimulation with different endogenous bile acids for 24 h (50 µmol L^−1^, LCA: 5 µmol L^−1^). Coomassie staining of SDS-PAGE gels was used as a loading control. Data are presented as mean bar plots with standard error. Single data points indicate biological replicates. *p < 0.05 vs. control, Kruskal–Wallis test with post-hoc Dunn’s test. Statistics: Figure Data, additional statistical information, and *p* values are provided in Supplementary Materials 2 and 3.

It has been described that RIPK3 activation can result in inflammation[13, 77]. Hepatocytes react to inflammatory stress with IL-8 expression, which plays a significant role in the activation of local and the attraction of circulating immune cells, particularly monocytes and neutrophils, that carry out the hepatic immune defense [78, 79]. RIPK3-FLAG expression in HepG2, readily resulting in phosphorylation (Fig. 3A), increased released IL-8 levels significantly (Fig. 4A). CA, which further increased RIPK3-FLAG phosphorylation, elevated secreted IL-8 protein levels slightly further and significantly to unstimulated HepG2 cells.

To further investigate the effects of RIPK3 activation on IL-8 protein release in HepG2 cells, we individually mutated both RIPK3 activation sites in our RIPK3-FLAG vector to aspartic acid (Asp), mimicking phosphorylation. However, those mutations hardly increased the IL-8 protein levels compared to wild-type RIPK3. Only the permanently active RIPK3[Ser199Asp, Ser227Asp] variant significantly increased secreted IL-8 levels compared to wild-type RIPK3 (Fig. 4B). RIPK3-FLAG wild-type also significantly stimulated *IL-8* gene expression in HepG2 cells (Fig. 4C).

Notably, unconjugated bile acids (CA, CDCA, UDCA, LCA) stimulated *IL-8* gene expression in the presence of RIPK3-FLAG significantly compared to the bile acid stimulation without previous RIPK3 expression. Conversely, incubation with most of their glycine- and taurine-conjugates again reduced the *IL-8* gene expression compared to their unconjugated counterparts.

The gene expression of other cytokines involved in hepatocytes’ inflammatory response [80–84], namely Interleukin-33 (IL-33), Interleukin-6 (IL-6), and *High-Mobility-Group-Protein B1 (HMGB1)*, were tested but were not altered significantly in RIPK3-FLAG transfected and CA-stimulated HepG2 cells (Fig. S11). Those findings suggest that RIPK3 activation by bile acids in HepG2 cells is predominantly associated with an IL-8 response (Fig. 4A–C, Fig. S11).

Previous work indicated IL-8 expression is controlled by different pathways, whereby the Jun-(N)-terminal kinase (JNK) signaling pathway is one prominent example [85, 86]. pJNK is not detected in naïve HepG2 cells throughout all experiments. Using anisomycin, a potent JNK activator, we confirmed the specificity of the pJNK antibody and HepG cell’s ability to maintain a JNK phosphorylation over 24 h with a peak after 30 min (Fig. S12). In the experiments, we utilized the four primary, unconjugated bile acids (CA, CDCA, UDCA, LCA) and TCDCA, which activated *IL-8* gene expression the strongest, to investigate JNK activation by Thr183/ Tyr185 phosphorylation. RIPK3-FLAG overexpression directly stimulated JNK phosphorylation significantly in an ELISA (Fig. S13) and was well detectable by western blotting (Fig. 4D). This finding also correlates with the immediate RIPK3-FLAG activating phosphorylation and increased IL-8 protein and gene expression in those cells (Fig. 3, Fig. 4A–D).

Stimulation with the hydrophilic bile acids (CA, UDCA) led to activation and phosphorylation of RIPK3-FLAG, further enhancing the phosphorylation of JNK significantly (Fig. 4D). Again, CA showed the most potent effects also on JNK phosphorylation in RIPK3-FLAG transfected HepG2 cells. However, CA also significantly stimulated JNK phosphorylation in HepG2 cells not transfected with the RIPK3 vector (Fig. S14).

Thus the expression of RIPK3 in hepatocytes but not necessarily its activation might cause the observed IL-8 expression, and the observed effects of IL-8 expression induced by bile acids are independent of RIPK3 signaling.

This notion was confirmed by transferring HepG2 cells once more with the different phosphor-positive, active, and phosphor-negative inactive RIPK3-FLAG variants. Quantifying pJNK levels by an ELISA 24 h after transfecting those mutants confirmed RIPK3 wild-type and all active and inactive variants independently stimulated JNK phosphorylation significantly (Fig. S13).

## Discussion

RIPK3 and MLKL phosphorylation are hallmarks of necroptosis, resulting in the formation of the necrosome, pMLKL pore formation, inflammation, and cell death [8, 87, 88].

Purified primary human and murine hepatocytes did not express RIPK3. Analyzing the RIPK3 promoter region with two independent methylation-specific sequencing methods returned up to 65% of promoter hypermethylation in human hepatocytes. A global hypermethylation loss was described in human hepatocellular carcinoma HepG2 cells [89]. However, compared to 4% RIPK3 promoter methylation in primary monocytes and 13% in non-parenchymal liver cells with previously confirmed RIPK3 expression [90], the remaining 35% RIPK3 promoter methylation in HepG2 cells was sufficient to suppress its expression. In line with a previous report, the hypomethylating agent, 5-aza-2′-deoxycytidine, induced expression RIPK3 in HepG2 cells after 24 h [58], strengthening RIPK3 promoter hypomethylation as the cause for its absence in hepatocytes.

Primary human hepatocytes are senisitive to cytokine signaling [91] and reacted to a mix of TNF-α, the pan-caspase inhibitor zVAD-fmk, and an inhibitor of the apoptosis protein (IAP) after 6 h with RIPK1 expression and a minor but significant MLKL phosphorylation without a detectable RIPK3 expression induction, that may point towards the recently discussed mechanism of a RIPK3 independent MLKL phosphorylation [88]. Only a 24-h-long stimulation with a pro-inflammatory mix of LPS and cytokines mimicking an inflammatory liver milieu in infection [59] conducted in HepG2, since they are phenotypically more stable than primary cells and do not undergo a culture-dependent phenotypic change [92], resulted in a detectable RIPK3 expression.

RIPK3 analysis from hepatocytes of different mouse models of inflammatory, cholestatic injury revealed an association of RIPK3 expression induction in models of chronic liver failure but not during an acute toxic liver insult.

In humans with chronic liver diseases, RIPK3 expression was confirmed by immunofluorescent staining. In addition, patients receiving liver surgery due to different tumor and liver diseases were divided into their bilirubin levels into two groups (cholestasis vs. non-cholestasis). Both groups did not significantly differ in their CRP levels, an acute-phase protein secreted by hepatocytes upon an inflammatory stimulus and a general clinical marker for inflammation. However, hepatocellular RIPK3 expression was significantly higher in patients with cholestasis than those without clinically diagnosed cholestasis.

These results demonstrate that chronic inflammatory diseased conditions, particularly if associated with cholestasis, induce RIPK3 expression and sensitize hepatocytes to necroptotic cell death.

To be able to explore the function of RIPK3 in hepatocytes, we selected the human hepatoma HepG2 cell line since they do not express RIPK3 endogenously and are utilized as a representative model of the human liver by various laboratories since they display a high degree of morphological and functional differentiation to generate reproducible results [93].

Profound phosphorylation was detectable when the RIPK3-FLAG construct was expressed in HepG2 cells. We challenged the model with different bile acids at concentrations reported to be present in human liver tissue during cholestasis [33]. Bile acids are endogenously actively uptaken through highly specialized basolateral transporters in Hepatocytes, such as NTCP and OATPs [94]. However, HepG2 cells do not express NTCP [95, 96], contributing the LC–MS confirmed bile acid uptake into the cells to an OATP activity and diffusion [97].

The bile acids CA, UDCA, and their metabolites induced RIPK3-FLAG phosphorylation even over the activation already observed from overexpressing the protein alone. We observed a phospho-serine (Ser) 227 RIPK3-FLAG positive double band also detectable using the RIPK3 antibody [8, 98], particularly after bile acid stimulation. As shown by Chen 2013, RIPK3 contains multiple phosphorylation sites (Ser199, Ser227) that exhibit different functions and are indispensable for necroptosis induction [98]. Ser199 is the critical residue for inducing kinase activity [76], whereas Ser227 is crucial for generating necroptosis [98]. Additionally, ubiquitination was reported to be regulated during RIPK3 activation [99]. Mutating the RIPK3 ubiquitinylation site (Lys5) did not change the bile acid-induced phosphorylation and mass shift. However, incubating the stimulated samples with CIP and lambda phosphatase prior-gel loading diminished the mass shift, indicating RIPK3 hyperphosphorylation during the bile acid-mediated RIPK3 activation.

RIPK3 phosphorylation is known as the pacemaker of necroptosis. In HepG2 cells, the phosphorylation of the heterologous expressed RIPK3-FLAG resulted in downstream MLKL phosphorylation. CA could not trigger cell death in HepG2 cells, a potent activator of RIPK3 in this model, in the absence of RIPK3. RIPK3 expression resulted in an increased number of pRIPK3-positive and dead HepG2 cells. Those numbers increased significantly when HepG2 cells were additionally treated with CA. Those data suggest that bile acids, mainly CA, not only induce RIPK3 phosphorylation but also carry further inducing necroptosis and cell death in HepG2 cells.

Due to the excellent capacity for biotransformation and storage function of hepatocytes, an increase of conjugated bile acids is often observed first in the pathophysiology of diseases [100–102]. Similarly to CA, also TCA caused a significant RIPK3 hyperphosphorylation, which is crucial for the consecutive activation of necroptosis [103, 104]. TCA and TUDCA can facilitate cell survival and act as anti-cholestatic metabolites [105]. Our results further demonstrate that the same choleretic bile acids (TCA, TUDCA) can induce necroptosis in hepatocytes in various liver diseases associated with hepatocellular RIPK3 expression. This reveals a novel pathophysiological mechanism in the progression of liver diseases, with RIPK3 expression as a molecular switch determining the hepatocyte’s fate.

The hepatocellular accumulation of TCA, and in some cases also CA, during various liver injuries, supports the notion that hepatocytes employ necroptosis signaling to induce tissue remodeling and inflammation. Hyperphosphorylation of RIPK3 had been recognized previously as a critical event in the activation cascade of RIPK3 and necroptosis [75]. As mentioned before, Ser199 (kinase activity) and Ser227 (interaction with MLKL and induction of necroptosis) contain specific functions during the activation of RIPK3. Activation of RIPK1 induces the activation of RIPK3. The following hyperphosphorylation of RIPK3 induces the interaction with MLKL via the RIP homotypic interaction motif. The entire mechanism of RIPK3 hyperphosphorylation has yet to be elucidated. Still, it seems likely that activation of RIPK3 induces phosphorylation at Ser199, which in turn auto-phosphorylates Ser227 to form a stable complex with MLKL [98, 106].

Hydrophobic TCDCA also frequently increased during cholestasis, reducing the expression and phosphorylation of RIPK3. In the context of heterogeneous bile acid toxicity, these effects are known as the bile acid paradox [107, 108]. This effect further indicates that other unconjugated bile acids besides CA exert cell toxicity primarily by regulating apoptosis, and their function as a detergent in high concentrations may lead to direct tissue necrosis [44, 109–111]. The accumulation of bile acids may not represent the primary cause of liver injury but likely promote disease progression and chronification due to a chronic inflammatory response [33]. Further, this study’s findings support previous controversial results obtained from RIPK3-knockout mice after various types of experimental liver injury. RIPK3-knockout mice were specially protected from injuries that commonly result in chronic liver diseases (e.g., obstructive cholestasis [32, 112], ethanol-induced liver injury [20]), inflammatory liver diseases (e.g., sepsis [113]), and fatty liver-related liver diseases [23], but not in the situation of acute toxic damage as caused by, e.g., acetaminophen, that may lead to an accumulation of unconjugated bile acids in blood [26, 27] (Supplementary Table S1). In general, liver damage could be triggered by different modes of cell death. Besides apoptosis, alternative mechanisms are characterized on the molecular level, known as necrosis, necroptosis, or pyroptosis. It is essential to decipher the hepatic cell death mechanism for different liver diseases [112].

As RIPK3-knockout mice are protected from liver damage during different types of chronic injury, we postulate that the epigenetic profile, which is regulated in a highly dynamic manner during injury and liver regeneration, may be remodeled rapidly during cell stress and will modify hepatocyte susceptibility to endogenous metabolites, inflammatory signaling, and cell death.

While strong effects on RIPK3 phosphorylation were observed for CA, UDACA, and their metabolites stimulation after 24 h, CDCA, LCA, and their metabolites reduced RIPK3 expression and phosphorylation without inducing membrane damage. Those opposing effects indicate the high complexity of the underlying metabolic signaling network affecting even the heterologous RIPK3 expression and necroptosis on multiple levels. In light of the potent RIPK3 activation through CA, UDCA, and their metabolites, the epigenetic RIPK3 suppression suggests that hepatocytes protect themselves from necroptosis induced by noxious and endogenous metabolites such as bile acids.

Those findings lead to the hypothesis that RIPK3 silencing is a mechanism employed by hepatocytes to avoid RIPK3-dependent necroptosis under physiological conditions associated with the initiation of cytokine signaling and cell death due to MLKL pore formation [114].

Cell death studies report an inflammatory response to RIPK3 activation [19]. In HepG2 cells, the transfection with a RIPK3 vector immediately results in RIPK3 phosphorylation, increased *IL-8* gene expression, and IL-8 release into the supernatant. IL-8 is an early cytokine hepatocytes secrete to activate local macrophages and attract neutrophils and monocytes, stimulating the immune response if not canceled by anti-inflammatory signals [115, 116]. Unconjugated bile acids (CA, CDCA, UDCA, LCA) did not further increase *the IL-8 gene expression* in RIPK3-FLAG transfected HepG2 cells after 24 h. In contrast, GCA, the taurine and glycine bile acid metabolites decreased *IL-8* gene expression by roughly 50% compared to their unconjugated counterparts. This effect confirms other findings suggesting that the reduction of the hydrophobicity by bile acid conjugation is protective and, in turn, increased cell-protective properties due to increased hydrophilicity and impermeability to the cell membrane [44, 117]. Notably, RIPK3 expression and cholic acid stimulation did not significantly alter the gene expression of the hepatic cytokines *IL-6, IL-33*, and *HMGB1*. The *IL-8* gene expression found after transfection the RIPK3 vector in HepG2 further resulted in significantly elevated IL-8 levels in the supernatant. CA stimulation of RIPK3 transfected HepG2 cells increased the IL-8 levels in the supernatant even more profoundly. Also, the transfection of a permanently activated RIPK3 variant (RIPK3[Ser199Asp, Ser227Asp]) resulted in increased IL-8 supernatant concentrations, suggesting the RIPK3 activation dependent release of IL-8 that hepatocytes in vivo that is uncoupled from gene expression.

IL-8 expression had been described as a consequence of JNK phosphorylation, a pathway involved in several physiological and pathological inflammatory processes [118]. The JNK pathway is one of the three major groups of mitogen-activated protein kinases (MAPK), which plays a significant role in acute and chronic liver injuries by regulating the liver’s metabolism and cell death pathway [119]. In line with previous studies, we confirmed that CA activates the JNK pathway without RIPK3 expression, which inhibited bile acid synthesis in other work [120, 121]. Besides this, activation of JNK, in general, is also known to contribute to the expression of various pro-inflammatory cytokines (IL-8, IL-6, IL-17) [86, 122, 123]. Further, a RIPK3- and bile acid-dependent expression of IL-33 might contribute to JNK activation [124]. In our HepG2 model, however, a RIPK3- and bile acid-dependent transcriptional regulation could not be confirmed after 24 h.

JNK phosphorylation was increased in HepG2 cells through heterologous RIPK3 expression. However, levels of JNK phosphorylation did not differ when a permanently active RIPK3 or an active RIPK3 variant was transfected. This suggests that the RIPK3 expression, but not its activation drives JNK phosphorylation in HepG2 cells. Further, HepG cells transfected with RIPK3 and stimulated with CA and UDCA increased JNK phosphorylation. Those effects suggest that JNK phosphorylation can be induced independently by bile acids and RIPK3 expression, eventually adding up to each other in HepG2 cells.

How RIPK3 induces JNK phosphorylation precisely is still researched. Some work previously suggested a RIPK3-dependent MLKL-mediated JNK activation [125]. Since inactivated RIPK3 expression resulted in a solid JNK phosphorylation and indirect signaling, that still depends on RIPK3 activity seems unlikely in our HepG2 cell model.

Bile acid acts as a central signaling molecule in hepatocytes that may trigger inflammation in various ways. JNK activation, IL-8 expression, and release are associated with RIPK3 expression, which is silenced under physiological conditions. In the presence of RIPK3, cholic acid stimulates IL-8 release and might additively act on JNK phosphorylation signaling that may result in metabolic and inflammation adaptations.

Hepatocytes seemingly employ epigenetic RIPK3 silencing as an endogenous master switch protecting them, on the one hand, from endobiotic metabolites, e.g., bile acids that otherwise induce necroptosis but also RIPK3 mediated inflammation and JNK phosphorylation.

In summary, hepatocytes do not express RIPK3 under basal expression due to RIPK3 promoter methylation. However, inflammatory cytokines induced RIPK3 expression in vitro. Further, hyperbilirubinemia in patients was associated with higher RIPK3 expression in hepatocytes. CA efficiently triggered the key events characterizing necroptosis RIPK3 dependently. CA increased RIPK3 phosphorylation, downstream MLKL phosphorylation, cell death, and IL-8 release. Consequently, RIPK3 suppression under basal conditions can is a self-protective cell mechanism in hepatocytes since only RIPK3 silencing allows them to carry out biotransformation of endogenous bile acids without triggering necroptosis.

## Supplementary information

Supplementary Material - 1

Original Data File

Supplementary Material - 3

Original Data File

Authors Checklist

## Data Availability

The datasets generated and analyzed in this study are included in this published article and the Supplementary Information files. Additional data are available from the authors on reasonable request.
