# Supplementary material for: RIPK3 promoter hypermethylation in hepatocytes protects from bile acid-induced inflammation and necroptosis
Source: Cell Death Dis. 2023 Apr 18;14(4):275. doi: 10.1038/s41419-023-05794-0 (PMC10113265; doi:10.1038/s41419-023-05794-0)
Supplement: Supplementary file 1 — Supplementary Material - 1 [file 41419_2023_5794_MOESM1_ESM.pdf]

Supplementary Material - 1

RIPK3 promoter hypermethylation in hepatocytes protects from bile acid-induced inflammation and necroptosis

Jessica Hoff[1,2], Ling Xiong[1,2], Tobias Kammann[1,2], Sophie Neugebauer[3], Julia M. Micheel[1,2], Nikolaus Gaßler[4], Michael Bauer[1,2], Adrian T. Press[1,2,5]

[1] Department of Anesthesiology and Intensive Care Medicine, Nanophysiology Group, Jena University Hospital, Jena 07747, Germany
[2] Center for Sepsis Control and Care, Jena University Hospital, Jena 07743, Germany
[3] Department of Clinical Chemistry and Laboratory Diagnostics, Jena University Hospital, Jena 07747, Germany
[4] Pathology, Jena University Hospital, Jena 07747, Germany
[5] Faculty of Medicine, Friedrich Schiller University Jena, Jena 07747, Germany

Correspondence

Adrian Press,
Am Klinikum 1, 07747 Jena
+49 3641/ 9 323139
Adrian.Press@med.uni-jena.de

Keywords

necroptosis, hepatocytes, RIPK3, bile acids, methylation, inflammation

Supplementary Information – Methods

Cell culture

Cryopreserved primary human hepatocytes donor pools (pHep) with 20 male (average age: 36.9 years, average body mass index (BMI): 26.3) or 20 female (average age: 41.1 years, average BMI: 29.2) donors were purchased from Lonza, Switzerland. Experiments with the cells were performed as indicated in the Western Blot section.

LDH assay

Lactate dehydrogenase (LDH) activity can be used as a marker of cell membrane integrity to assess the cytotoxicity caused by compounds. For measuring the effects of the bile acids LCA, GLCA, and TLCA on HepG2 cells, the CytoTox 96 Non-Radioactive Cytotoxicity Assay (#G1780, Promega, Germany) was performed. Following incubation with the bile acids described in 'Stimulation with Bile Acids', 50 µL of cell supernatant was transferred into a 96-well plate and mixed with 50 µL of the substrate. After 30 min incubation, the stop solution was added, and absorbance was measured at 490 nm. The LDH release was determined by subtracting the media background and calculating the amount according to positive control.

5-Aza and cytokine mix stimulation

Cells were treated with 10 µmol L^{-1} 5-Azacytidine (5-Aza) (Biomol) or a cytokine mix (50 ng mL^{-1} TNF-α (Immunotools, Germany), 10 ng mL^{-1} IL-1β (Immunotools, Germany), 10 ng mL^{-1} IFN-γ (Immunotools, Germany), 100 ng mL^{-1} LPS (Prospec, Israel)) for 24 h in cell culture media.

Methylation analysis by pyrosequencing

According to the manufacturer's protocol, Genomic DNA was extracted from cell cultures using the DNeasy blood & tissue kit (Qiagen). Cells were resuspended in the proteinase K digestion buffer (40 µg proteinase K) and incubated at 50°C for 30 min. Cell debris was pelleted by centrifugation at 14000 g for 10 minutes. For DNA methylation analysis, 500 ng of the purified genomic DNA was bisulfite converted using the EZ DNA Methylation-Direct kit (Zymo Research). Bisulfite-treated DNA was purified according to the manufacturer's protocol and was eluted to a final volume of 46 µL. PCRs were performed using 1 µL bisulfite-treated DNA and 10 µmol L^{-1} of each target-validated primer from the EpigenDx Assay (ADS1678FP, ADS1678RPB). One primer was biotin-labeled and HPLC-purified to purify the final PCR product using sepharose beads. PCR products were bound to Streptavidin Sepharose HP (GE Healthcare Life Sciences) for immobilization. Afterward, the immobilized PCR products were purified, washed, denatured with 0.2 µmol L^{-1} NaOH solution, and washed using the Pyrosequencing Vacuum Prep Tool (Qiagen) as stated in the manufacturer's protocol. A methylation assay ADS1678 (EpigenDx) was performed, which reports the methylation of eight representative CpG sites in the regulatory region (**Tab. 1**). 10 µL of the PCR products were sequenced by pyrosequencing on the PSQ96 HS system (Qiagen) following the manufacturer's instructions. The methylation status of each CpG site was determined individually using QCpG software (Qiagen). The software calculates the level at each CpG site as the percentage of the methylated alleles divided by the sum of all methylated and unmethylated alleles.

Methylation analysis by Illumina sequencing

DNA was generated as described in paragraph 'Methylation Analysis'. In addition, 67 ng of the purified genomic DNA was bisulfite converted, amplified, and sequenced for

DNA methylation analysis. A methylation assay was performed by Zymo Research, which reports the methylation of 21 representative CpG sites (**Tab S6**). The PCR product was sequenced by Illumina sequencing following the manufacturer's instructions. Zymo Research calculated the methylation status of each CpG site.

Imaging cell death and necroptosis

HepG2 cells were seeded, transfected, and stimulated as described in sections *Cell isolation and culture*, *Transfection*, and *Stimulation with bile acids*.

For co-staining, transfected and stimulated HepG2 cells were stained with ThermoFisher fixable Live/Dead Stain (LIVE/DEAD™ Fixable Orange (602) Viability Kit, for 561 nm excitation, #L34983, 1:1000, ThermoFisher Scientific) for 30 min. The cells were then fixed for 15 min in 4% formalin (Roti Histofix, Carl Roth, Germany). After fixation, the permeabilization (0.1% Triton-X100 in TBS, 10 min, RT) and blocking (5% donkey serum, 1 h, RT) followed. Next, the primary antibody pRIPK3 (Ser227) (D6W2T) (#93654, Cell Signaling Technology) diluted 1:500 in TBS was left on the cells overnight at 4 °C. The next day the cells were washed twice with TBS and secondary antibody (Chicken anti-Rabbit IgG (H+L) Cross-Adsorbed Secondary Antibody, Alexa Fluor 647, #A-21443, ThermoFisher Scientific) diluted 1:500 in blocking buffer was added for 1 h, rocking at ambient temperature. Then, after two more washing steps with TBS, the nuclei were stained with 5 µg mL-1 H33342 in type-1 water. After two final washing steps, the samples were stored in PBS at 4 °C.

Imaged on a laser scanning microscope (LSM-780) with optimized filter setting for the three fluorescent molecules, H33342 (λ_{ex}: 390-405 nm (diode laser), λ_{em}: 410-556 nm), live dead stain (λex: 561 nm (DSPP laser), λem: 570-650) and Alexa Fluor 647 stained pRIPK3 (λex: 633 nm (HeNe laser), λem: 651-755 nm). Multiple images were taken

with a 20x plan-apochromat (NA 0.8) objective and a resolution of 1.2044 px µm $^{-1}$ on each well. In addition, three individually seeded stimulated and treated wells were imaged. Images were manually analyzed for the staining pattern with the FIJI distribution of ImageJ and the Cell Counter Plugin. (PMID: 27911396) Cells positive for the nucleus stain (H33342), live dead stain (Live/Dead Fixable Orange 602), pRIPK3 staining, or double and triple positive cells were distinguished.

JNK ELISA

Transfection was performed as described in paragraph 'Transfection' and stimulation as described in paragraph' Stimulation with Bile Acids'. HepG2 cells were washed with PBS and lysed in the RIPA buffer, and the protein concentration was measured with the BCA Protein Assay Macro Kit according to the manufacturer's protocol (Serva Gelelectrophoresis GmbH). In addition, 50 µL of the total lysate was used for the JNK ELISA. The assay was performed following the manufacturer's instruction using human JNK 1/2 (pT183/Y185 + Total) ELISA (Abcam). Absorbance was measured at 450 nm using a plate reader (EnSpire, Perkin Elmer).

Supplementary Information – Figures

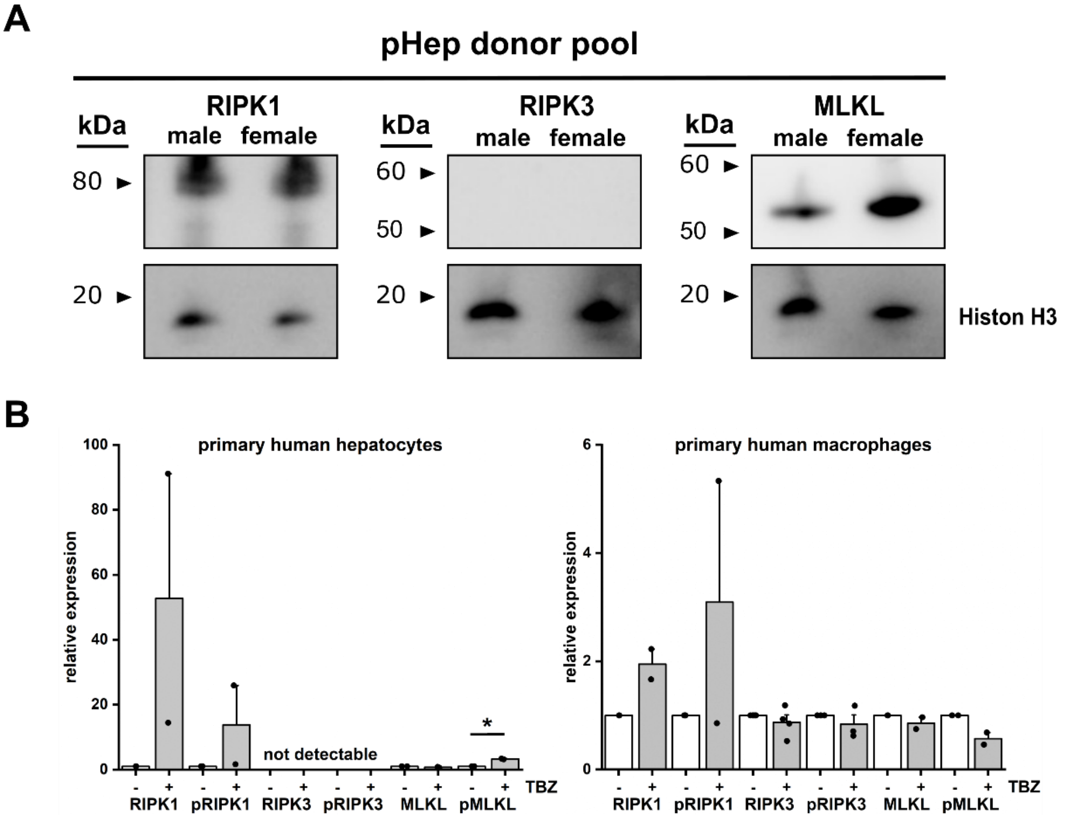

Supplementary Figure S1: Analysis of RIPK1, RIPK3, and MLKL. (**A**) Protein expression of molecules in the necroptosis signaling pathway was detected in male and female pHep donor pools (20 donors each). 20 µg proteins were loaded. Histone H3 was used as a loading control. (**B**) Densiometric analysis of the western blot results in Figure 1 C. * $p<0.05$ vs. no (-) TBZ, unpaired t-test (hepatocytes: Welch's t-test; macrophages: RIPK1/ pRIPK1/ MLKL/ pMLKL with Welch's t-test; RIPK3 with Mann-Whitney test and pRIPK3 with Student's t-test).

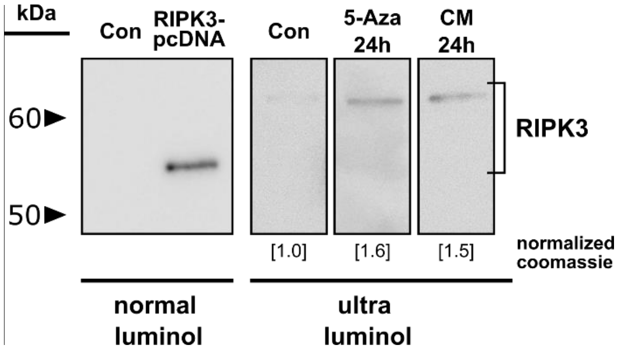

Supplementary Figure S2: Hypomethylating agents and cytokine mix trigger RIPK3 expression in HepG2 cells. Protein expression of RIPK3 detected in HepG2 after treatment with 5-Azacytidine (5-Aza) (10 µmol L^{-1}) or a cytokine mix (50 ng mL^{-1} TNF-α, 10 ng mL^{-1} IL-1β, 10 ng mL^{-1} IFN-γ, 100 ng mL^{-1} LPS) for 24 h. In addition, 20 µg of proteins were loaded. Coomassie-stained gels were used as a loading control.

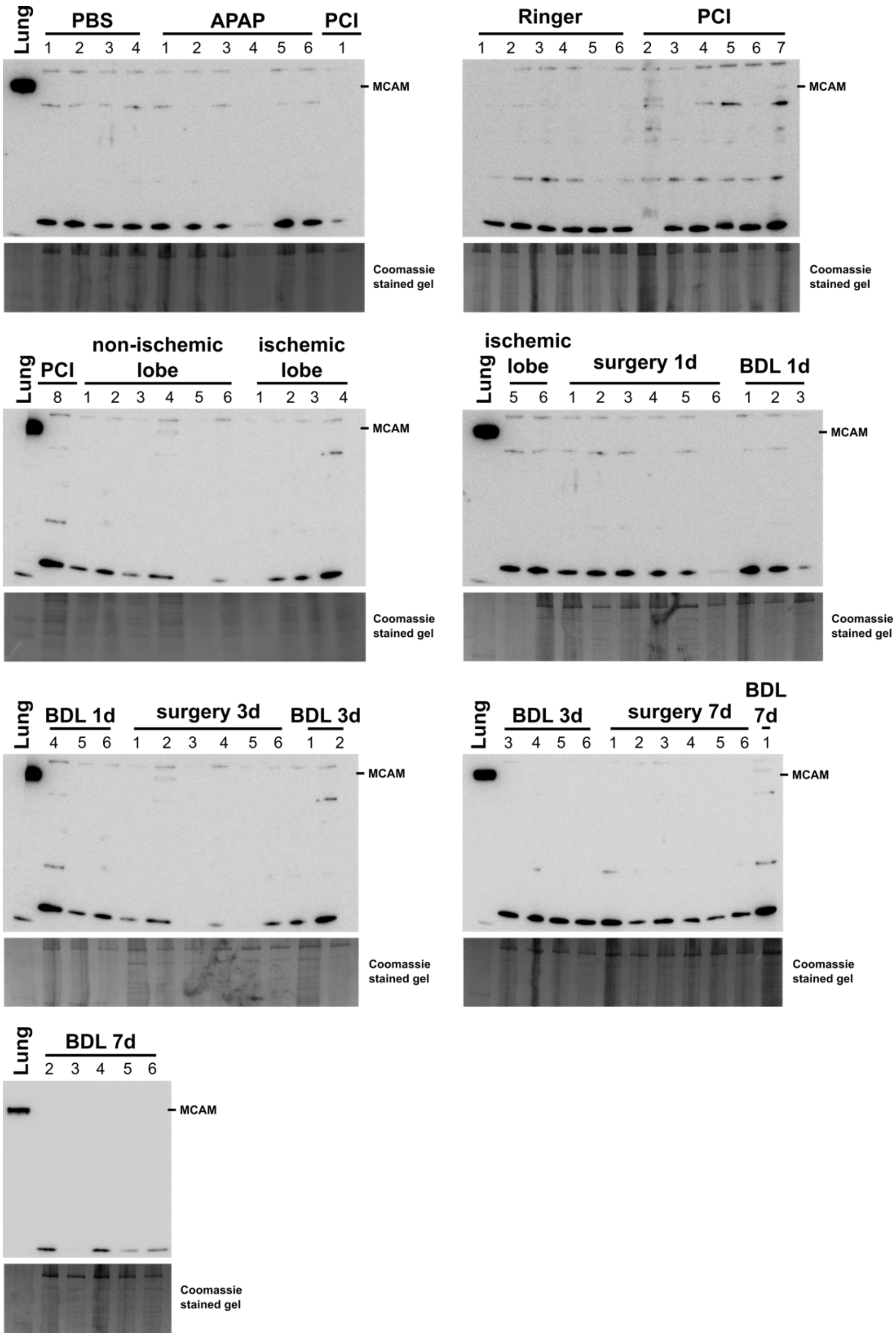

Supplementary Figure S3: Purity of primary murine hepatocytes. Western blot analyzed the endothelial cell marker melanoma cell adhesion molecule (MCAM/ CD146) in different primary murine hepatocytes. Lung lysate was used as a positive control. In addition, 10 µg proteins were loaded. Coomassie-stained gels were used as a loading control.

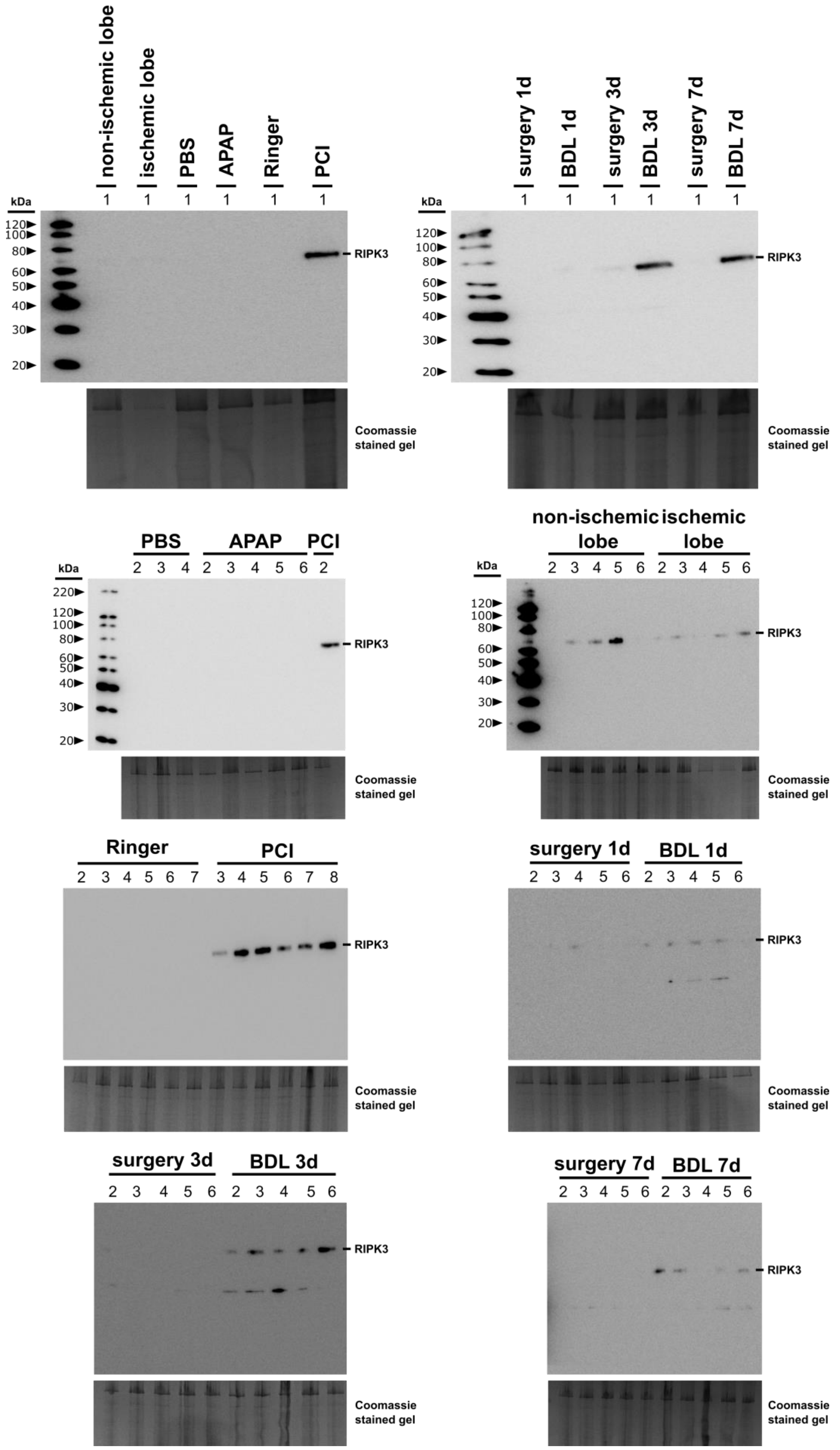

Supplementary Figure S4: RIPK3 expression in primary murine hepatocytes. Western blot analyzed the protein expression of RIPK3 in different primary murine hepatocytes. 10 µg proteins were loaded. Coomassie-stained gels were used as a loading control.

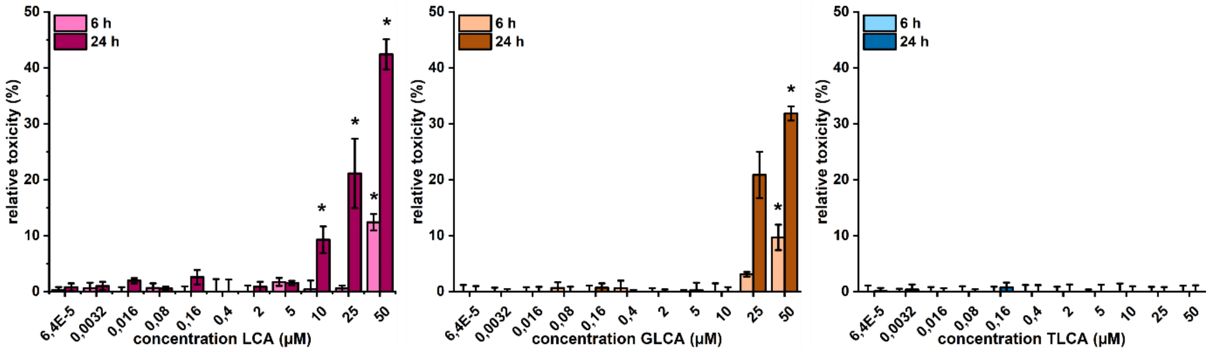

Supplementary Figure S5: Cytotoxicity of (un)conjugated lithocholic acid. HepG2 cells were exposed to lithocholic acid (LCA), its glycine- (GLCA), and taurine- (TLCA) conjugated metabolites for 24 h in defined concentrations. The LDH assay investigated toxicity. Data are presented as a mean bar plot with standard error after subtracting the control. The experiment was performed twice with n=4 per group. * $p<0.05$ vs. concentration 6,4E-5, Kruskal-Wallis test with post-hoc Dunn's test; GLCA 6h: one-way ANOVA with post-hoc Dunnett's test.

condition	transporter specificity (Thomas et al. 2008)	property	stimulation	total bile acid concentration in HepG2 cells after 24 h (µmol L^{-1})	effect on RIPK3 phosphorylation (fold-change to untreated control) (Fig. 3, Fig. S6)
control				0.00	-
LCA	-	hydrophobic	5 µmol L^{-1}	0.29	0.5
GLCA	NTPC, OATP, BSEP, OST	↓	10 µmol L^{-1}	0.23	0.1
TLCA			50 µmol L^{-1}	0.71	0.4
CDCA	-			0.33	1.7
GCDCA	NTPC, OATP, BSEP, OST			0.15	1.0
TCDCA				0.19	0.2
CA	-			0.12	3.5
GCA	NTPC, OATP, BSEP, OST			0.07	1.5
TCA				0.14	2.3
UDCA	-			0.14	4.6
GUDCA	NTPC, OATP, BSEP, OST			0.05	2.0
TUDCA		hydrophilic		0.09	2.4

stimulated \ measured	bile acids											
	LCA	GLCA	TLCA	CDCA	GCDCA	TCDCA	CA	GCA	TCA	UDCA	GUDCA	TUDCA
control	nd	nd	nd	nd	nd	nd	nd	nd	nd	nd	nd	nd
LCA	0.29	nd	nd	nd	nd	nd	nd	nd	nd	nd	nd	nd
GLCA	nd	0.16	0.08	nd	nd	nd	nd	nd	nd	nd	nd	nd
TLCA	nd	nd	0.71	nd	nd	nd	nd	nd	nd	nd	nd	nd
CDCA	nd	nd	nd	0.33	nd	nd	nd	nd	nd	nd	nd	nd
GCDCA	nd	nd	nd	0.07	0.09	nd	nd	nd	nd	nd	nd	nd
TCDCA	nd	nd	nd	0.06	nd	0.13	nd	nd	nd	nd	nd	nd
CA	nd	nd	nd	0.06	nd	nd	0.06	nd	nd	nd	nd	nd
GCA	nd	nd	nd	nd	nd	nd	nd	0.07	nd	nd	nd	nd
TCA	nd	nd	nd	nd	nd	nd	nd	nd	0.14	nd	nd	nd
UDCA	nd	nd	nd	nd	nd	nd	nd	nd	nd	0.14	nd	nd
GUDCA	nd	nd	nd	nd	nd	nd	nd	nd	nd	nd	0.05	nd
TUDCA	nd	nd	nd	nd	nd	nd	nd	nd	nd	0.04	nd	0.05

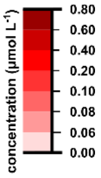

Supplementary Figure S6: Mean bile acid concentration (µmol L^{-1}) in HepG2 cells. HepG2 cells were stimulated with different bile acids for 24 h (50 µmol L^{-1}, LCA: 5 µmol L^{-1}, GLCA: 10 µmol L^{-1}). The concentration of each bile acid was determined by LC-MS/MS measurement. Data are presented as absolute values. nd: not detected. n=3 per group.

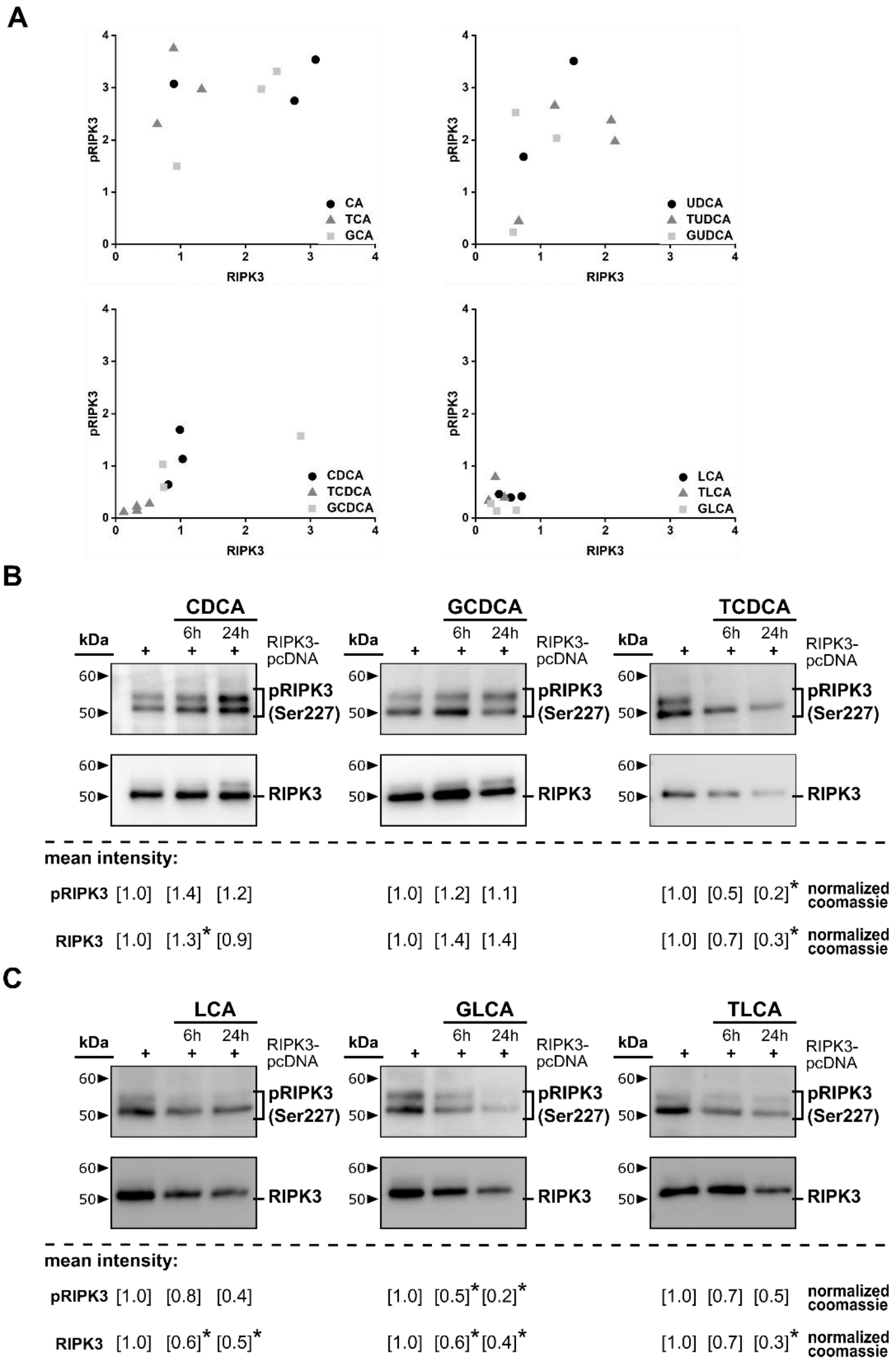

	unconjugated	Glycine conjugated	Taurine conjugated
**CA**	3	3	3
**UDCA**	3	4	4
**CDCA**	3	3	4
**LCA**	3	3	3

Supplementary Figure S7: Analysis of RIPK3 phosphorylation and expression. (**A**) Representation of individual values of RIPK3 and pRIPK3 expression analyzed by WB. (**B-C**) RIPK3 phosphorylation and expression upon stimulation with different endogenous bile acids (50 µmol L^{-1}, LCA: 5 µmol L^{-1}, GLCA: 10 µmol L^{-1}) for 6 or 24 h. Stimulation with un-, taurine (T)- or glycine (G)-conjugated (**B**) chenodeoxycholic acid (CDCA) and (**C**) lithocholic acid (LCA). (**B-C**) Coomassie staining of SDS-PAGE gel was used as a loading control. n=3-4 per group. Heatmap shows significant differences (green) between the treatment and control groups. (**B**) * $p<0.05$ vs. control, RIPK3: CDCA/ TCDCA with one-way ANOVA with post-hoc Dunnett's test, GCDCA with one-way ANOVA; pRIPK: CDCA/ GCDCA with one-way ANOVA and TCDCA with Kruskal-Wallis test. (**C**) * $p<0.05$ vs. control, RIPK3: one-way ANOVA with post-hoc Dunnett's test; pRIPK: LCA/ TLCA with one-way ANOVA and GLCA one-way ANOVA with post-hoc Dunnett's test.

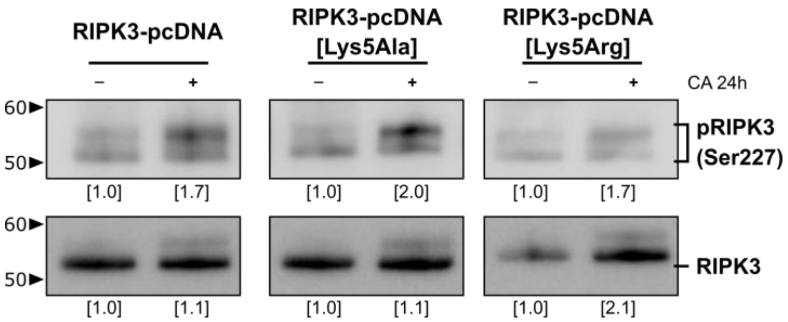

Supplementary Figure S8: Analysis of ubiquitination-induced RIPK3 band mass shift. RIPK3 expression and phosphorylation in HepG2 cells transfected with a mutated plasmid at the ubiquitination site Lys5 to alanine (Ala, permanent inhibition) and arginine (Arg, permanent activation) and stimulated with CA (50 µmol L^{-1}) were analyzed by western blot in a single experiment.

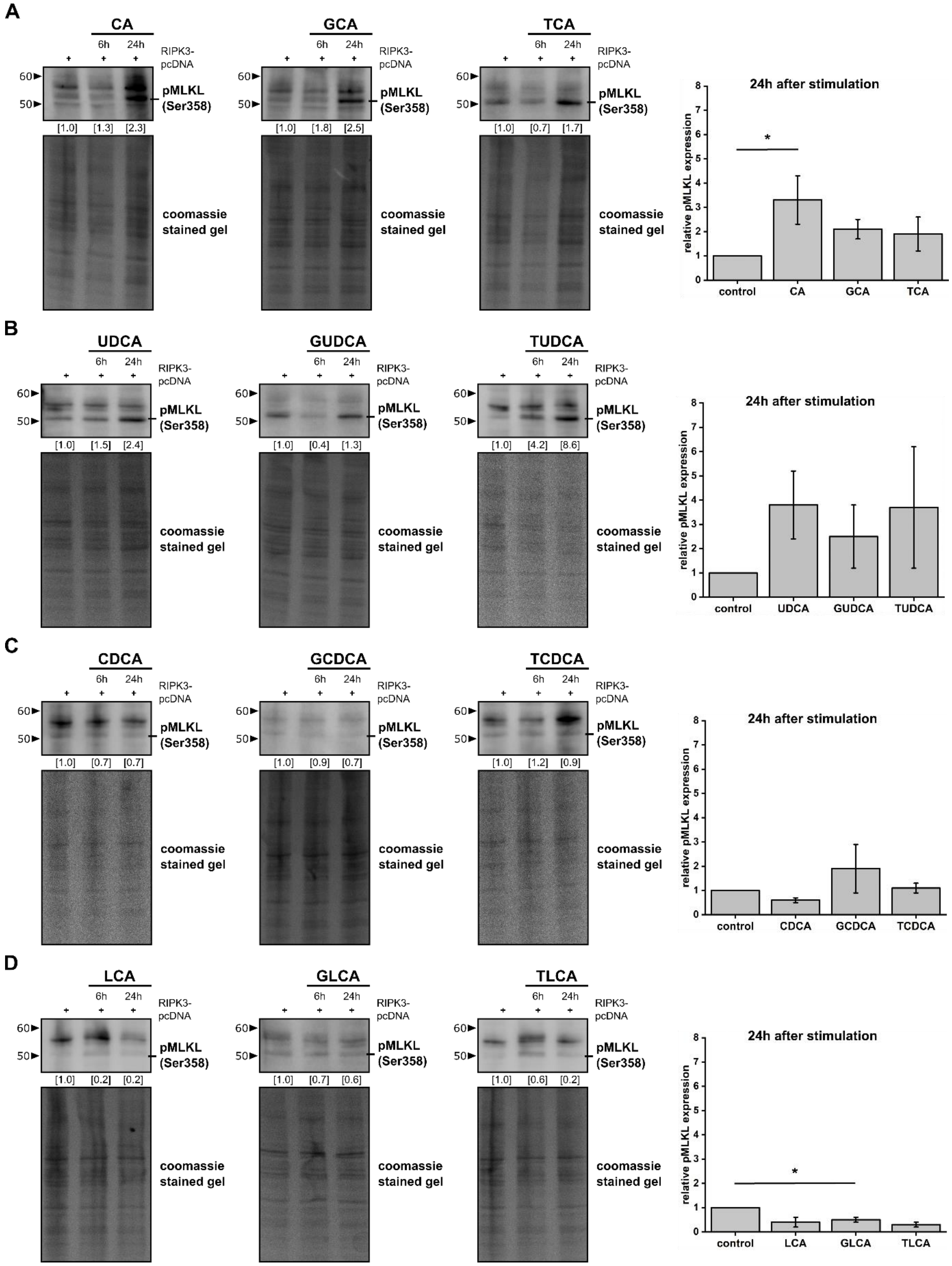

182

	unconjugated	Glycine conjugated	Taurine conjugated
**CA**	2	2	3
**UDCA**	2	2	2
**CDCA**	2	2	3
**LCA**	2	2	2

Supplementary Figure S9: Analysis of MLKL phosphorylation. (**A-D**) MLKL phosphorylation upon stimulation with different endogenous bile acids (50 µmol L^{-1}, LCA: 5 µmol L^{-1}, GLCA: 10 µmol L^{-1}) for 6 or 24 h. Stimulation with un-, taurine (T)- or glycine (G)-conjugated (**A**) cholic acid (CA), (**B**) ursodeoxycholic acid (UDCA), (**C**) chenodeoxycholic acid (CDCA), and (**D**) lithocholic acid (LCA). (**A-D**) Coomassie staining of SDS-PAGE gel was used as a loading control. n=2-3 per group. * $p<0.05$ vs. control, (**A**) one-way ANOVA with post-hoc Dunnett's test, (**B**) Kruskal-Wallis test with post-hoc Dunn's test, (**C**) Kruskal-Wallis test and (**D**) Kruskal-Wallis test with post-hoc Dunn's test.

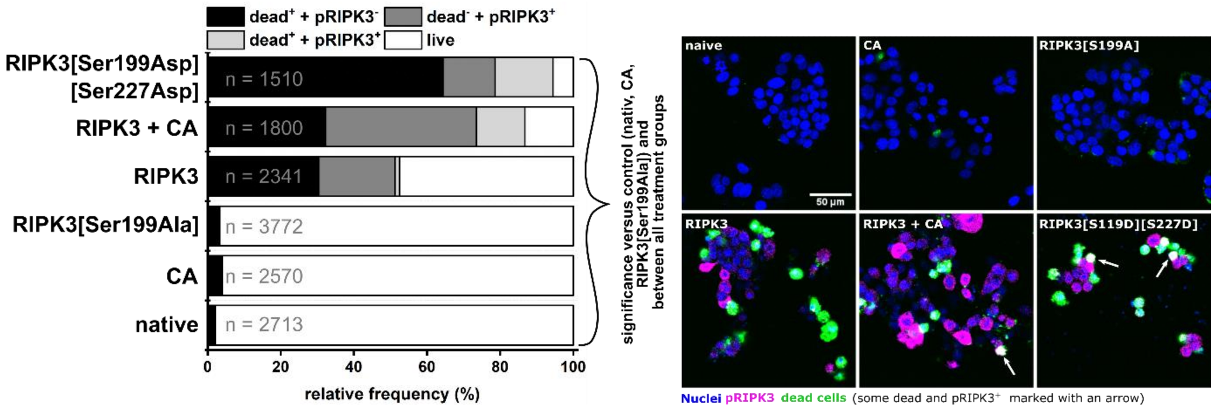

Supplementary Figure S10: Cell death analysis with fixable Live/Dead staining. HepG2 cells were transfected with RIPK3/ RIPK3 mutants (24 h) and stimulation with cholic acid (CA) (24 h; 50 µmol L-1). The relative frequency bar depicts the percentage of live, dead, and pRIPK3-positive cells. Representative micrographs are on the right side. Cells were counterstained with Hoechst 33342 (blue). The numbers analyzed for three experiments are given as n for each condition with the bar plot. Bonferroni-adjusted p-value due to 9 conditions: * $p<0.0056$ vs. control (native, CA, RIPK3[Ser199Ala]), Chi-Square test. Specific p-values could be taken from the attached document with significance tests (PDF file).

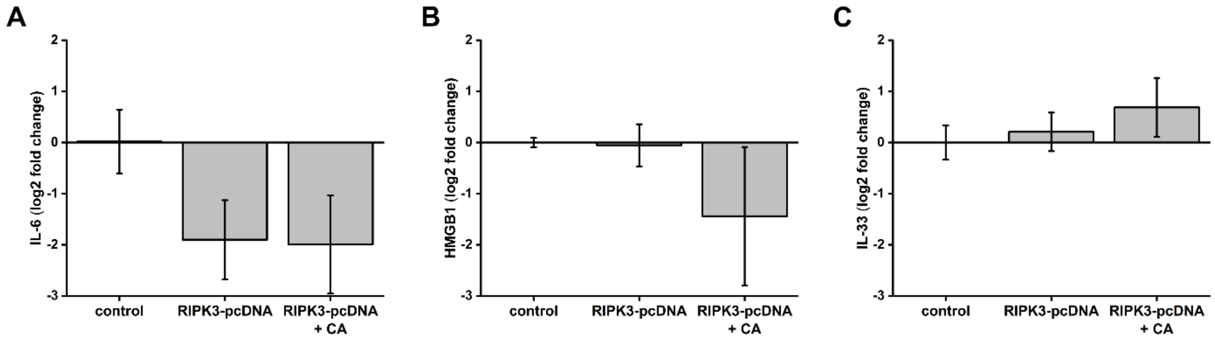

Supplementary Figure S11: mRNA-Expression of cytokines associated with liver inflammation. mRNA levels of IL-6, HMBG1, and IL-33 in HepG2 cells with and without transfection of the RIPK3-pcDNA and stimulation with CA (50 µmol L^{-1}). Data are presented as log2 fold change. n=2-7 per group. No significant differences were observed when tested with * $p<0.05$ vs. control, IL-33, and HMBG1 (Kruskal-Wallis test); IL-6 (one-way ANOVA).

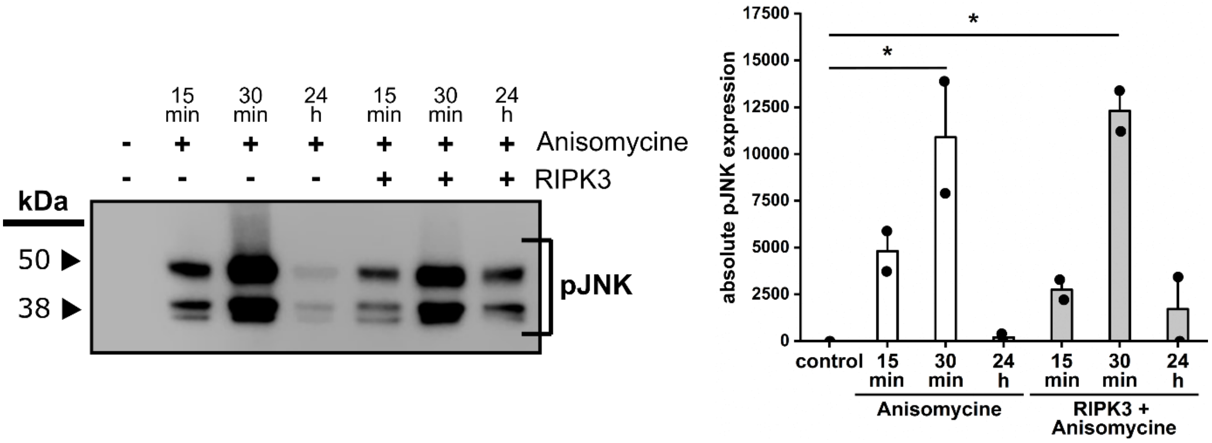

Supplementary Figure S12: Time-dependent induction of JNK phosphorylation. JNK phosphorylation in HepG2 cells upon transfection with RIPK3-pcDNA (1 µg) and stimulation with Anisomycine (50 ng mL-1), an activator of JNK, for 15 min, 30 min, and 24 h. Coomassie staining of SDS-PAGE gel was used as a loading control. Single data points indicate biological replicates. * p<0.05 vs. control, Kruskal-Wallis test with post-hoc Dunn's test.

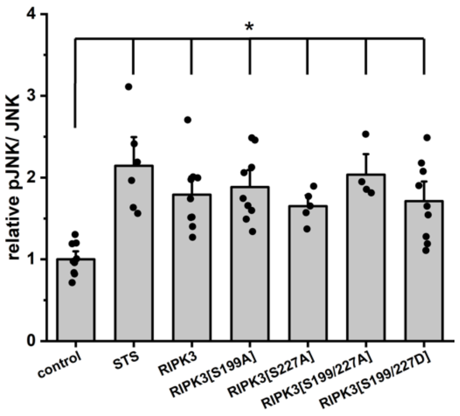

Supplementary Figure S13: RIPK3-dependent activation of JNK. An ELISA measured JNK activation. Data are presented as a mean bar plot with standard error. Staurosporine (STS) (17 h; 1 µmol L^{-1}) served as positive control. Single data points reflect individual replicates. * $p<0.05$ vs. control, one-way ANOVA with post-hoc Dunnett's test.

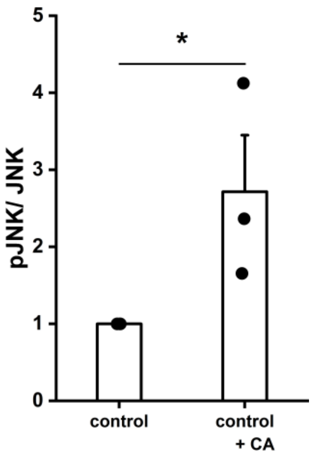

Supplementary Figure S14: RIPK3 independent activation of JNK. JNK expression and phosphorylation in HepG2 cells upon transfection with RIPK3-pcDNA (1 µg) and stimulation with CA for 24 h (50 µmol L^{-1}). Coomassie staining of SDS-PAGE gels was used as a loading control. Data are presented as a mean bar plot with standard error and individual data points. * $p<0.05$ vs. control, one-tailed t-test (Student's t-test).

Supplementary Information – Tables

Supplementary Table S1: Overview of the effects of RIPK3 deficiency in different liver disease models. Red: knock-out (KO) shows no protection; green: KO shows protection. * literature mentioned in the manuscript.

	model	technique	results	literature
severe	**acetaminophen-induced liver injury (APAP)**	RIPK1 inhibition (necrostatin-1, Nec-1)	protective against hepatic injury	PMID: 25349782; PMID: 24440347
		RIPK1 inhibition (Nec-1) or RIPK3-KO	protective against injury, reduced inflammation	30*
		RIPK3-KO	not protective against APAP injury	27*
		RIPK3-KO mice with RIPK1 knock-down (KD)/ RIPK1 inhibition (Nec-1)	protective against APAP toxicity	27*
		MLKL-KO	not protective	27*; PMID: 27756058
	**Concanavalin A (ConA; autoimmune hepatitis)**	RIPK1 inhibition (Nec-1)	protective against ConA-induced liver injury, reduced inflammation	PMID: 24198446
		RIPK1 inhibition (Nec-1)	exacerbates hepatitis with increased cell death	30*
		RIPK3-KO	delayed but not protected from hepatic injury	30*
		RIPK3-KO	no protection from ConA-induced hepatic injury	PMID: 27756058
		RIPK3-KO	no protection from liver damage	31*
		MLKL-KO	attenuation of ConA-induced liver damage	PMID: 27756058

	**ischemia-reperfusion injury (IR)**	RIPK1 inhibition (Nec-1)	no protective effects on hepatic IR injury	28*
		RIPK3-KO	no protective effects on early hepatic IR injury (4-6h) but in the later phase (24h)	28*
		MLKL-KO	protected from IR liver injury, reduced inflammation	PMID: 31026418
**chronic**		**RIPK3 expression** is **increased** in human/ murine liver biopsies with **chronic liver diseases**		20*; PMID: 26769846, PMID: 26201023
	**alcoholic hepatitis**	RIPK1 inhibition (Nec-1)	no attenuation of ethanol-induced liver injury	20*
		RIPK3-KO	prevents liver injury following ethanol feeding, reduced inflammation	20*
		RIPK3-KO	more resistant to alcohol-induced liver injury, decreased inflammation	PMID: 26769846
	**nonalcoholic steatohepatitis (NASH)/ nonalcoholic fatty liver disease (NAFLD)**	RIPK3-KO (high-fat diet (HFD))	not protected against HFD-induced liver injury associated with increased inflammation and apoptosis	PMID: 27301788
		RIPK3-KO (methionine-choline-deficient (MCD))	protected from MCD-induced NASH and inflammation	23*
		RIPK3-KO (MCD)	improvement of hepatic damage and inflammation during MCD-induced NASH	PMID: 26201023
		RIPK3-KO (carbon tetrachloride (CCl_4))	not protective against CCl_4-induced liver fibrosis	23*

Supplementary Table S2: Characterization of primary human hepatocytes (single donor, donor pool). (Lonza, Switzerland)

parameter	hHep $_{\text{single donor}}$	hHep ♂$_{\text{DP20}}$	hHep♀$_{\text{DP20}}$
**number of donors**	1 male	20 female	20 male
**donor age average**	45 years	36.9 years	41.1 years
**donor BMI average**	24.2	26.3	29.2
**donor background**<br>**Asian**<br>**Afro America**<br>**Caucasian**<br>**others**	 0.0%<br>0.0%<br>100.0%<br>0.0%	 5.0%<br>5.0%<br>80.0%<br>10.0%	 0.0%<br>10.0%<br>85.0%<br>5.0%
**no drugs/ alcohol/ tobacco**	no information	45.0%	60.0%
**use of**<br>**illicit drugs**<br>**tobacco**<br>**heavy alcohol**	no information	 35.0%<br>35.0%<br>5.0%	 20.0%<br>20.0%<br>5.0%
**serologies**<br>**CMV**<br>**EBV**<br>**HBV**<br>**HCV**<br>**HIV**	no information	 positive<br>positive<br>negative<br>negative<br>negative	 positive<br>positive<br>negative<br>negative<br>negative

Supplementary Table S3: Overview of different liver disease parameters. Values are presented as mean±SD. PBS: phosphate-buffered saline without calcium and magnesium, BDL: Bile Duct Ligation, APAP: Acetaminophen-induced liver injury, PCI: Peritoneal Contamination and Infection, i.p.: intraperitoneal, no values: insufficient amount plasma obtained to analyze biochemical parameters.

model	change in body weight within 24 h (g)	ALAT (µmol $(L*s)^{-1}$)	ASAT (µmol $(L*s)^{-1}$)
**PBS i.p.**	2.4 ± 0.4	0.9 ± 0.1	4.8 ± 1.1
**Ringer acetate i.p.**	0.5 ± 0.5	0.8 ± 0.1	4.4 ± 0.5
**abdominal surgery 1 d**	-0.4 ± 0.7	1.0 ± 0.2	5.6 ± 0.7
**abdominal surgery 3 d**	-1.0 ± 1.1	no values	4.6 ± 0.8
**abdominal surgery 7 d**	-0.8 ± 0.7	0.6 ± 0.1	3.0 ± 0.3
**BDL surgery 1 d**	-1.4 ± 0.6	no values	no values
**BDL surgery 3 d**	-2.1 ± 1.4	10.0 ± 1.3	23.5 ± 3.6
**BDL surgery 7 d**	-1.6 ± 1.4	10.4 ± 0.8	13.2 ± 0.5
**ischemia-reperfusion injury 1 h**	-1.5 ± 0.4	7.7 ± 1.9	21.0 ± 7.6
**APAP 24 h**	-0.4 ± 1.0	21.7 ± 19.8	13.6 ± 10.3
**PCI 24 h**	-2.4 ± 0.7	0.9 ± 0.1	4.3 ± 0.4

Supplementary Table S4: Summary of disease parameters from human liver sections. CI: confidence interval; SD: standard deviation. * $p<0.05$ vs. reference group. For bilirubin, aspartate aminotransferase (ASAT), alanine aminotransferase (ALAT), and C-reactive protein (CRP), the Mann-Whitney test was applied (data are not normally distributed). For albumin, the unpaired t-test (Student's t-test) was performed (normality and equal distribution present).

	**reference group** (bilirubin < 21 µmol L^{-1})	**cholestasis group** (bilirubin ≥ 21 µmol L^{-1})
**mean age ± CI**	57 ± 6.4 years	59 ± 10.1 years
**gender ratio**	12 female, 8 male	4 female, 5 male
**bilirubin ± SD**	11.6 ± 4.3 µmol L^{-1}	74.4 ± 56.3 µmol L^{-1} *
**ASAT ± SD**	0.5 ± 0.2 µmol L^{-1}	1.6 ± 2.6 µmol L^{-1} *
**ALAT ± SD**	0.5 ± 0.4 µmol L^{-1}	0.6 ± 0.4 µmol L^{-1}
**albumin ± SD**	34.8 ± 6.4 g L^{-1}	29.9 ± 3.8 g L^{-1} *
**C-reactive protein ± SD**	52.5 ± 79.3 mg L^{-1}	14.7 ± 17.5 mg L^{-1}
**disease entities included**	metastatic sigma carcinoma, Klatskin carcinoma, metastatic insulinoma, metastatic colon carcinoma, metastatic rectal carcinoma, adenoma, breast carcinoma, liver hematoma, hepatocellular carcinoma, adenocarcinoma (rectal, ovary, colon, pancreas, stomach), squamous cell carcinoma, nonalcoholic steatohepatitis	metastatic colon carcinoma, metastatic cecum carcinoma, gall bladder carcinoma, Klatskin carcinoma, adenocarcinoma (pancreas), liver cirrhosis, nonalcoholic steatohepatitis

Supplementary Table S5: The manuscript's sequences and melting temperature of used primers. Tm: melting temperature that was used for annealing in the site-directed mutagenesis

name	forward primer (5' -> 3')	reverse primer (5' -> 3')
**IL-6**	CAGTTCCTGCAGAAAAAGGCAA	AGCTGCGCAGAATGAGATGA
**IL-8**	CAGTTTTGCCAAGGAGTGCT	ACTTCTCCACAACCCTCTGC
**IL-33**	CCTCATCATCTGAGACCAGCAC	TGGCCTTCTGTTGGGATTTTCC
**HMGB1**	AAGGGTCATCACACACGGAG	GCCCATGTTTAGTTATTTTTCGTGA
**HPRT**	TGACACTGGCAAAACAATGCA	GGTCCTTTTCACCAGCAAGCT
**[Ser199Ala] (Tm: 70°C)**	CCGGAAGGCC**GCC**ACAGCCAGTG	TTTACGTTAACAAACAGTTCTGGGGCCAAG
**[Ser227Ala] (Tm: 53°C)**	AACCGAACCA**GCC**CTCGTGTACG	GGCAACTCAACTTCTCTTC
**[Ser199Asp] (Tm: 62°C)**	CCGGAAGGCC**GAC**ACAGCCAGTG	TTTACGTTAACAAACAGTTCTGG
**[Ser227Asp] (Tm: 53°C)**	AACCGAACCA**GAC**CTCGTGTACG	GGCAACTCAACTTCTCTTC
**[Lys5Ala] (Tm: 70°C)**	GTCGTGCGTC**GCC**TTATGGCCCAGCGGTGCC	ATCTCGAGCGGCCGCCAG
**[Lys5Arg] (Tm: 68°C)**	GTCGTGCGTC**AGA**TTATGGCCCAGCGGTGC	ATCTCGAGCGGCCGCCAG

Supplementary Table S6: n-number table for western blot analysis of Figure 1C.

	**HepG2**	**pHep** (single donor)*	**pHep** (pool, 20 donors)*	**pMac**
**RIPK1**	3	2		2
**pRIPK1**	3	2		2
**RIPK3**	5	4	2 *(1 male, 1 female)*	4
**pRIPK3**	3	3		3
**MLKL**	2	2		2
**pMLKL**	2	2		2

* the basal characterization of the hepatocytes and donor pools are now provided in supplementary **Table S1**

Supplementary Table S7: DNA methylation state of human RIPK3 promotor. RIPK3 promoter methylation analysis in human hepatocytes detected by Illumina sequencing (Zymo Research). Values depict the mean percentage of methylated cytosine in the different promoter regions of each sample. Sequencing was performed on 1.5 µg of DNA. pHep: primary hepatocyte; NPC: primary non-parenchymal cells (Kupffer cells, cholangiocytes, liver sinusoidal endothelial cells, stellate cells, and other immune cells from the liver. The ratio between different cell types had been not characterized).

chromosome 14, position 24809110 to 248099315

CGGCAGGTGCTCAGGAAA**CG**CTAGTCAAGGAGAAAGGCACTAGCTTT**CG**CTCTGC
CCCCTGCCCCACCCCAGGGG**CG**GGACTGTAGAGG**CG**CCTATAAGGGAAGTTGTTC
AGTCAACT**CG**GAAAAAGGGTAACAACC**CG**GAAAGTAGACTCAC**CG**TCTTGGTCTAG
AGACTGACCCCTGCACAGACAGACCCCTTCCCCTCTCTG**CG**

	**mean methylation of 9 CpG regions in individual samples or donor pools**
**pHep (20 male donors)**	53%
**pHep (20 female donors)**	63%
**NPC (single female donor)**	13%
