# Supplementary material for: RIPK3 promoter hypermethylation in hepatocytes protects from bile acid-induced inflammation and necroptosis
Source: Cell Death Dis. 2023 Apr 18;14(4):275. doi: 10.1038/s41419-023-05794-0 (PMC10113265; doi:10.1038/s41419-023-05794-0)
Supplement: Supplementary file 2 — Original Data File [file 41419_2023_5794_MOESM2_ESM.pdf]

Supplementary Material - 2

RIPK3 promoter hypermethylation in hepatocytes protects from bile acid-induced inflammation and necroptosis

Jessica Hoff[1,2], Ling Xiong[1,2], Tobias Kammann[1,2], Sophie Neugebauer[3], Julia M. Micheel[1,2], Nikolaus Gaßler[4], Michael Bauer[1,2], Adrian T. Press[1,2,5]

[1] Department of Anesthesiology and Intensive Care Medicine, Nanophysiology Group, Jena University Hospital, Jena 07747, Germany
[2] Center for Sepsis Control and Care, Jena University Hospital, Jena 07743, Germany
[3] Department of Clinical Chemistry and Laboratory Diagnostics, Jena University Hospital, Jena 07747, Germany
[4] Pathology, Jena University Hospital, Jena 07747, Germany
[5] Faculty of Medicine, Friedrich Schiller University Jena, Jena 07747, Germany

Correspondence
Adrian Press,
Am Klinikum 1, 07747 Jena
+49 3641/ 9 323139
Adrian.Press@med.uni-jena.de

Keywords
necroptosis, hepatocytes, RIPK3, bile acids, methylation, inflammation

Supplementary Information – Raw data

Figure 1C

RESULTS EPIGENDX (PYROSEQUENCING)

**From TSS**	-65	-47	-18	11	25	54	73	89	-65 to +89			
**GRCh38/hg38**	Chr14:24340110	Chr14:24340092	Chr14:24340063	Chr14:24340035	Chr14:24340021	Chr14:24339992	Chr14:24339973	Chr14:24339957	Chr14:24340110-24339957			
**Location**	5-Upstream	5-Upstream	5-Upstream	5-UTR	5-UTR	5-UTR	5-UTR	5-UTR	5-Upstream to 5-UTR			
**pHep**	70.0	78.5	83.3	59.7	73.7	67.7	58.7	35.7	65.9	14.9	35.7	83.3
	69.7	76.1	83.1	57.1	75.3	55.5	51.6	38.3	63.3	15.1	38.3	83.1
**mean %**	**70**	**77**	**83**	**58**	**74**	**62**	**55**	**37**				
**HepG2**	38.3	38.6	50.8	19.9	39.7	35.6	39.2	43.1	38.1	8.7	19.9	50.8
	35.8	39.2	48.9	22.0	36.6	18.3	17.5	39.7	32.2	11.5	17.5	48.9
**mean %**	**37**	**39**	**50**	**21**	**38**	**27**	**28**	**41**				
**pMak**	0.0	6.5	0.0	0.0	6.2	15.6	15.9	4.8	6.1	6.6	0.0	15.9
	0.0	3.3	4.5	3.1	4.2	3.5	0.0	0.0	2.3	2.0	0.0	4.5
**mean %**	**0**	**5**	**2**	**2**	**5**	**10**	**8**	**2**				

Figure 1D

Raw data included in figure S1 and table S6.

Figure 2B

group
0: Sham
1: Treatment

expression
0: no Effect
1: Effect

PCI

animal	Group	Expression
**1**	0	0
**2**	0	0
**3**	0	0
**4**	0	0
**5**	0	0
**6**	0	0
**7**	0	0
8	1	1
9	1	1
10	1	1
11	1	1
12	1	1
13	1	1
14	1	1
15	1	1

(%)	Sham	PCI
expression	0	8
no expression	7	0

APAP

animal	Group	Expression
**1**	0	0
**2**	0	0
**3**	0	0
**4**	0	0
5	1	0
6	1	0
7	1	0
8	1	0
9	1	0
10	1	0

(%)	Sham	APAP
expression	0	0
no expression	4	6

IR

animal	Group	Expression
**1**	0	0
**2**	0	0
**3**	0	1
**4**	0	1
**5**	0	1
**6**	0	1
7	1	1
8	1	1
9	1	1
10	1	1
11	1	1
12	1	1

(%)	Sham	IR
expression	4	6
no expression	2	0

BDL 1

animal	Group	Expression
**1**	0	0
**2**	0	0
**3**	0	0
**4**	0	1
**5**	0	0
**6**	0	0
7	1	1
8	1	1
9	1	1
10	1	1
11	1	1
12	1	1

(%)	Sham	BDL1
expression	1	6
no expression	5	0

BDL 3

animal	Group	Expression
**1**	0	1
**2**	0	1
**3**	0	0
**4**	0	0
**5**	0	0
**6**	0	0
7	1	1
8	1	1
9	1	1
10	1	1
11	1	1
12	1	1

(%)	Sham	BDL3
expression	2	6
no expression	4	0

BDL 7

animal	Group	Expression
**1**	0	1
**2**	0	0
**3**	0	0
**4**	0	0
**5**	0	0
**6**	0	0
7	1	1
8	1	1
9	1	1
10	1	?
11	1	1
12	1	1

(%)	Sham	BDL7
expression	1	5
no expression	5	0

	expression	no expression	amount	relative expression
**PBS**	0	7	7	0.0
**APAP**	0	6	6	0.0
**Ringer**	0	4	4	0.0
**PCI**	8	0	8	1.0
**surgery 1d**	1	5	6	0.2
**BDL 1d**	6	0	6	1.0
**surgery 3d**	2	4	6	0.3
**BDL 3d**	6	0	6	1.0
**surgery 7d**	1	5	6	0.2
**BDL 7d**	5	0	5	1.0
**non-ischemic**	4	2	6	0.7
**ischemic**	6	0	6	1.0

intensity	Ringer	PCI	surgery 1d	BDL 1d	surgery 3d	BDL 3d	sugery 7d	BDL 7d	PBS	APAP	non-ischemic	ischemic
	0.0	16464.2	0.0	602.1	1306.0	23904.8	319.0	23876.8	0.0	0.0	0.0	490.6
	0.0	1343.4	0.0	5341.1	326.8	15638.6	0.0	15489.4	0.0	0.0	0.0	781.5
	0.0	13253.9	0.0	10567.1	0.0	3758.4	0.0	6131.1	0.0	0.0	2250.8	1918.0
	0.0	15372.0	6148.5	13551.5	0.0	2173.0	0.0		0.0	0.0	4947.7	299.2
	0.0	4641.9	0.0	13792.7	0.0	6430.2	0.0	2015.6		0.0	20555.6	2137.9
	0.0	5748.9	0.0	5524.0	0.0	1453.2	0.0	4703.0		0.0	244.8	6526.6
	0.0	19537.4										
		15738.7										
**mean**	**0**	**11513**	**1025**	**8230**	**272**	**8893**	**53**	**10443**	**0**	**0**	**4666**	**2026**

Figure 2C-D

Name	Category 1	Category 2	CA	TCA	GCA	CDCA	TCDCA	GCDCA	UDCA	TUDCA	GUDCA	LCA	TLCA	GLCA
1	BDL	7d	0.05	1.74	0.05	0.05	0.85	0.05	0.05	0.05	0.03	0.06	0.05	0.05
2			0.05	3.10	0.05	0.05	0.96	0.05	0.05	0.05	0.03	0.06	0.05	0.05
3			0.05	1.09	0.05	0.05	0.04	0.05	0.05	0.05	0.03	0.06	0.05	0.05
4			0.05	0.99	0.05	0.05	0.60	0.05	0.05	0.05	0.03	0.06	0.05	0.05
5			0.05	0.59	0.05	0.05	0.66	0.05	0.05	0.06	0.03	0.06	0.05	0.05
6			0.05	1.02	0.05	0.05	0.80	0.05	0.05	0.10	0.03	0.06	0.05	0.05
			**0.05**	**1.42**	**0.05**	**0.05**	**0.65**	**0.05**	**0.05**	**0.06**	**0.03**	**0.06**	**0.05**	**0.05**
1	shamOP	7d	0.05	0.39	0.05	0.05	0.04	0.05	0.05	0.05	0.03	0.06	0.05	0.05
2			0.05	0.13	0.05	0.05	0.04	0.05	0.05	0.05	0.03	0.06	0.05	0.05
3			0.05	0.41	0.05	0.05	0.04	0.05	0.05	0.05	0.03	0.06	0.05	0.05
4			0.05	0.65	0.05	0.05	0.04	0.05	0.05	0.05	0.03	0.06	0.05	0.05
5			0.05	0.69	0.05	0.05	0.04	0.05	0.05	0.05	0.03	0.06	0.05	0.05
6			0.05	0.67	0.05	0.05	0.04	0.05	0.05	0.05	0.03	0.06	0.05	0.05
			**0.05**	**0.49**	**0.05**	**0.05**	**0.04**	**0.05**	**0.05**	**0.05**	**0.03**	**0.06**	**0.05**	**0.05**
1	BDL	3d	0.05	7.98	0.05	0.05	0.04	0.05	0.05	0.05	0.03	0.06	0.05	0.05
2			0.05	4.03	0.05	0.05	0.04	0.05	0.05	0.05	0.03	0.06	0.05	0.05
3			0.05	3.23	0.05	0.05	0.04	0.05	0.05	0.05	0.03	0.06	0.05	0.05
4			0.07	0.52	0.05	0.05	0.04	0.05	0.05	0.05	0.03	0.06	0.05	0.05
5			0.05	3.17	0.05	0.05	0.07	0.05	0.05	0.05	0.03	0.06	0.05	0.05
6			0.05	2.68	0.05	0.05	0.04	0.05	0.05	0.05	0.03	0.06	0.05	0.05
			**0.05**	**3.60**	**0.05**	**0.05**	**0.05**	**0.05**	**0.05**	**0.05**	**0.03**	**0.06**	**0.05**	**0.05**
1	shamOP	3d	0.05	0.44	0.05	0.05	0.04	0.05	0.05	0.05	0.03	0.06	0.05	0.05
2			0.05	0.40	0.05	0.05	0.04	0.05	0.05	0.05	0.03	0.06	0.05	0.05
3			0.05	0.15	0.05	0.05	0.04	0.05	0.05	0.05	0.03	0.06	0.05	0.05
4			0.05	0.09	0.05	0.05	0.04	0.05	0.05	0.05	0.03	0.06	0.05	0.05
5			0.05	0.28	0.05	0.05	0.04	0.05	0.05	0.05	0.03	0.06	0.05	0.05
6			0.05	0.30	0.05	0.05	0.04	0.05	0.05	0.05	0.03	0.06	0.05	0.05
			**0.05**	**0.28**	**0.05**	**0.05**	**0.04**	**0.05**	**0.05**	**0.05**	**0.03**	**0.06**	**0.05**	**0.05**
1	BDL	1d	0.16	10.90	0.05	0.05	0.23	0.05	0.05	0.05	0.03	0.06	0.05	0.05
2			0.45	21.70	0.05	0.05	0.34	0.05	0.05	0.05	0.03	0.06	0.05	0.05
3			0.05	3.45	0.05	0.05	0.04	0.05	0.05	0.05	0.03	0.06	0.05	0.05
4			0.05	5.40	0.05	0.05	0.13	0.05	0.05	0.05	0.03	0.06	0.05	0.05
5			0.05	3.97	0.05	0.05	0.12	0.05	0.05	0.05	0.03	0.06	0.05	0.05
6			0.05	3.80	0.05	0.05	0.05	0.05	0.05	0.05	0.03	0.06	0.05	0.05
			**0.14**	**8.20**	**0.05**	**0.05**	**0.15**	**0.05**	**0.05**	**0.05**	**0.03**	**0.06**	**0.05**	**0.05**
1	shamOP	1d	0.06	0.99	0.05	0.05	0.04	0.05	0.05	0.05	0.03	0.06	0.05	0.05
2			0.05	0.90	0.05	0.05	0.04	0.05	0.05	0.05	0.06	0.06	0.05	0.05
3			0.05	0.96	0.05	0.05	0.04	0.05	0.05	0.05	0.03	0.06	0.05	0.05
4			0.05	0.73	0.05	0.05	0.04	0.05	0.05	0.05	0.03	0.06	0.05	0.05
5			0.05	0.60	0.05	0.05	0.04	0.05	0.05	0.05	0.03	0.06	0.05	0.05
6			0.05	0.86	0.05	0.05	0.04	0.05	0.05	0.05	0.03	0.06	0.05	0.05
			**0.05**	**0.84**	**0.05**	**0.05**	**0.04**	**0.05**	**0.05**	**0.05**	**0.03**	**0.06**	**0.05**	**0.05**
1	Sham (PBS)	24h	0.05	0.05	0.05	0.05	0.04	0.05	0.05	0.05	0.03	0.06	0.05	0.05
2			0.05	0.23	0.05	0.05	0.04	0.05	0.05	0.05	0.03	0.06	0.05	0.05
3			0.05	0.26	0.05	0.05	0.04	0.05	0.05	0.05	0.03	0.06	0.05	0.05
4			0.05	0.18	0.05	0.05	0.04	0.05	0.05	0.05	0.03	0.06	0.05	0.05
			**0.05**	**0.18**	**0.05**	**0.05**	**0.04**	**0.05**	**0.05**	**0.05**	**0.03**	**0.06**	**0.05**	**0.05**
1	APAP	24h	0.05	0.32	0.05	0.05	0.04	0.05	0.05	0.05	0.03	0.06	0.05	0.05
2			0.05	0.36	0.05	0.05	0.04	0.05	0.05	0.05	0.03	0.06	0.05	0.05
3			0.05	0.68	0.05	0.05	0.04	0.05	0.05	0.05	0.03	0.06	0.05	0.05
4			0.05	0.62	0.05	0.05	0.04	0.05	0.05	0.05	0.03	0.06	0.05	0.05
5			0.05	0.36	0.05	0.05	0.04	0.05	0.05	0.05	0.03	0.06	0.05	0.05
6			0.05	0.43	0.05	0.05	0.04	0.05	0.05	0.05	0.03	0.06	0.05	0.05
			**0.05**	**0.46**	**0.05**	**0.05**	**0.04**	**0.05**	**0.05**	**0.05**	**0.03**	**0.06**	**0.05**	**0.05**
1h	IR healthy	24h	0.05	0.51	0.05	0.05	0.04	0.05	0.05	0.05	0.03	0.05	0.05	0.05
2h			0.05	0.37	0.05	0.05	0.04	0.05	0.05	0.05	0.03	0.05	0.05	0.05
3h			0.05	0.78	0.05	0.05	0.04	0.05	0.05	0.05	0.03	0.05	0.05	0.05
4h			0.05	2.29	0.05	0.05	0.04	0.05	0.05	0.05	0.03	0.05	0.05	0.05
5h			0.05	0.85	0.05	0.05	0.04	0.05	0.05	0.05	0.03	0.05	0.05	0.05
6h			0.05	0.22	0.05	0.05	0.04	0.05	0.05	0.05	0.03	0.05	0.05	0.05
			**0.05**	**0.83**	**0.05**	**0.05**	**0.04**	**0.05**	**0.05**	**0.05**	**0.03**	**0.05**	**0.05**	**0.05**
1d	IR diseased	24h	0.05	0.22	0.05	0.05	0.04	0.05	0.05	0.05	0.03	0.06	0.05	0.05
2d			0.05	0.16	0.05	0.05	0.04	0.05	0.05	0.05	0.03	0.06	0.05	0.05
3d			0.05	0.37	0.05	0.05	0.04	0.05	0.05	0.05	0.03	0.06	0.05	0.05
4d			0.05	0.62	0.05	0.05	0.04	0.05	0.05	0.05	0.03	0.06	0.05	0.05
5d			0.05	0.16	0.05	0.05	0.04	0.05	0.05	0.05	0.03	0.09	0.05	0.05
6d			0.05	0.73	0.05	0.05	0.08	0.05	0.05	0.05	0.03	0.06	0.05	0.05
			**0.05**	**0.38**	**0.05**	**0.05**	**0.05**	**0.05**	**0.05**	**0.05**	**0.03**	**0.06**	**0.05**	**0.05**
1	PCI	24h	0.05	0.24	0.05	0.05	0.04	0.05	0.05	0.05	0.03	0.06	0.05	0.05
2			0.05	0.86	0.05	0.05	0.18	0.05	0.05	0.05	0.03	0.06	0.05	0.05
3			0.05	0.33	0.05	0.05	0.04	0.05	0.05	0.05	0.03	0.06	0.05	0.05
4			0.05	0.29	0.05	0.05	0.05	0.05	0.05	0.05	0.03	0.06	0.05	0.05
5			0.05	0.68	0.05	0.05	0.07	0.05	0.05	0.05	0.03	0.06	0.05	0.05
6			0.05	1.54	0.05	0.05	0.16	0.05	0.05	0.05	0.03	0.06	0.05	0.05
7			0.05	1.77	0.05	0.05	0.18	0.05	0.05	0.08	0.03	0.06	0.05	0.05
8			0.05	2.20	0.05	0.05	0.12	0.05	0.05	0.05	0.03	0.06	0.05	0.05
			**0.05**	**0.99**	**0.05**	**0.05**	**0.11**	**0.05**	**0.05**	**0.05**	**0.03**	**0.06**	**0.05**	**0.05**
1	Sham (Ringer)	24h	0.05	0.35	0.05	0.05	0.08	0.05	0.05	0.05	0.03	0.06	0.05	0.05
2			0.07	0.87	0.05	0.05	0.12	0.05	0.05	0.05	0.03	0.06	0.05	0.05
3			0.05	0.71	0.05	0.05	0.07	0.05	0.05	0.05	0.03	0.06	0.05	0.05
4			0.05	0.71	0.05	0.05	0.08	0.05	0.05	0.05	0.03	0.06	0.05	0.05
5			0.05	0.34	0.05	0.05	0.07	0.05	0.05	0.05	0.03	0.06	0.05	0.05
6			0.05	0.26	0.05	0.05	0.08	0.05	0.05	0.05	0.03	0.06	0.05	0.05
7			0.05	0.64	0.05	0.05	0.06	0.05	0.05	0.05	0.03	0.06	0.05	0.05
8			0.05	0.67	0.05	0.05	0.09	0.05	0.05	0.05	0.03	0.06	0.05	0.05
			**0.05**	**0.57**	**0.05**	**0.05**	**0.08**	**0.05**	**0.05**	**0.05**	**0.03**	**0.06**	**0.05**	**0.05**

Figure 2F

LSEC				hepatocytes			
**Sample (according Tab S3)**	**reference**	**Sample (according Tab S3)**	**cholestasis**	**Sample (according Tab S3)**	**reference**	**Sample (according Tab S3)**	**cholestasis**
**24**	1865.20	**42**	494.98	**24**	921.56	**42**	3419.04
**31**	1913.27	**50**	3239.38	**31**	1602.81	**50**	2200.11
**25**	316.86	**57**	5233.78	**25**	1828.89	**57**	3348.48
**38**	2805.14	**56**	5511.54	**38**	2310.19	**56**	1644.36
**40**	1028.55	**49**	3977.03	**40**	621.61	**49**	4286.78
**41**	1321.71	**18**	6132.45	**41**	526.30	**18**	1819.59
**51**	1577.10	**15**	5392.04	**51**	703.18	**15**	907.22
**1**	6045.96	**17**	4203.95	**1**	707.62	**17**	3731.72
**2**	6045.96	**19**	6723.28	**2**	1088.40	**19**	2497.51
**3**	5247.43			**3**	1252.48		
**5**	2426.13	**mean**	**4545.38**	**5**	1368.12	**mean**	**2650.53**
**6**	3372.77	**standard deviation**	**1868.73**	**6**	1014.63	**standard deviation**	**1112.00**
**7**	4458.98	**amount**	**9.00**	**7**	2501.78	**amount**	**9.00**
**8**	4139.76	**standard error**	**622.91**	**8**	2401.13	**standard error**	**370.67**
**9**	5080.78			**9**	1758.95		
**10**	3033.05			**10**	1832.93		
**11**	4705.88			**11**	1832.93		
**12**	4925.20			**12**	3400.57		
**13**	2722.20			**13**	1771.54		
**14**	5004.28						
**mean**	**3401.81**			**mean**	**1549.77**		
**standard deviation**	**1743.39**			**standard deviation**	**757.15**		
**amount**	**20.00**			**amount**	**19.00**		
**standard error**	**389.83**			**standard error**	**173.70**		

Figure 3B

	CA	TCA	GCA	CDCA	TCDCA	GCDCA	UDCA	TUDCA	GUDCA	LCA	TLCA	GLCA
Hep G2	n.d.	n.d.	n.d.	n.d.	n.d.	n.d.	n.d.	n.d.	n.d.	n.d.	n.d.	n.d.
Hep G2	n.d.	n.d.	n.d.	n.d.	n.d.	n.d.	n.d.	n.d.	n.d.	n.d.	n.d.	n.d.
Hep G2	n.d.	n.d.	n.d.	n.d.	n.d.	n.d.	n.d.	n.d.	n.d.	n.d.	n.d.	n.d.
Hep G2	n.d.	n.d.	n.d.	n.d.	n.d.	n.d.	n.d.	n.d.	n.d.	n.d.	n.d.	n.d.
Hep G2	n.d.	n.d.	n.d.	n.d.	n.d.	n.d.	n.d.	n.d.	n.d.	n.d.	n.d.	n.d.
Hep G2	n.d.	n.d.	0.0953	n.d.	0.0808	0.103	n.d.	n.d.	n.d.	n.d.	n.d.	n.d.
Hep G2	n.d.	n.d.	n.d.	n.d.	n.d.	n.d.	n.d.	n.d.	n.d.	n.d.	n.d.	n.d.
Hep G2	n.d.	n.d.	n.d.	n.d.	n.d.	n.d.	n.d.	n.d.	n.d.	n.d.	n.d.	n.d.
Hep G2	n.d.	n.d.	n.d.	n.d.	n.d.	0.0494	n.d.	n.d.	n.d.	n.d.	n.d.	n.d.
Hep G2	n.d.	n.d.	n.d.	n.d.	0.0654	0.0537	n.d.	n.d.	n.d.	n.d.	n.d.	n.d.
Hep G2	n.d.	n.d.	n.d.	n.d.	0.0633	0.0536	n.d.	n.d.	n.d.	n.d.	n.d.	n.d.
Hep G2	n.d.	n.d.	n.d.	n.d.	0.0626	0.0526	n.d.	n.d.	n.d.	n.d.	n.d.	n.d.
	n.d.	n.d.	**0.0953**	n.d.	**0.068025**	**0.06246**	n.d.	n.d.	n.d.	n.d.	n.d.	n.d.

Figure 3C

CA

**RIPK3**	n=1	n=2	n=3	n=4	**Mean**	**standard deviation**	**standard error**	**pRIPK3**	n=1	n=2	n=3	n=4	**Mean**	**standard deviation**	**standard error**
**control**	1.0	1.0	1.0		**1.0**	**0.0**	**0.0**	**control**	1.0	1.0	1.0		**1.0**	**0.0**	**0.0**
**CA 6h**	0.6	1.6	1.5		**1.2**	**0.6**	**0.3**	**CA 6h**	1.4	1.2	1.7		**1.5**	**0.3**	**0.1**
**CA 24h**	0.9	2.8	3.1		**2.2**	**1.2**	**0.7**	**CA 24h**	3.1	2.7	3.5		**3.1**	**0.4**	**0.2**
**control**	1.0	1.0	1.0		**1.0**	**0.0**	**0.0**	**control**	1.0	1.0	1.0		**1.0**	**0.0**	**0.0**
**TCA 6h**	0.2	0.5	0.8		**0.5**	**0.3**	**0.2**	**TCA 6h**	0.7	0.7	1.0		**0.8**	**0.2**	**0.1**
**TCA 24h**	1.3	0.9	0.6		**1.0**	**0.3**	**0.2**	**TCA 24h**	3.0	3.8	2.3		**3.0**	**0.7**	**0.4**
**control**	1.0	1.0	1.0		**1.0**	**0.0**	**0.0**	**control**	1.0	1.0	1.0		**1.0**	**0.0**	**0.0**
**GCA 6h**	0.9	0.9	1.4		**1.1**	**0.3**	**0.2**	**GCA 6h**	1.0	0.9	1.3		**1.1**	**0.2**	**0.1**
**GCA 24h**	0.9	2.2	2.5		**1.9**	**0.8**	**0.5**	**GCA 24h**	1.5	3.0	3.3		**2.6**	**1.0**	**0.6**

Figure 3D

UDCA

**RIPK3**	n=1	n=2	n=3	n=4	**Mean**	**standard deviation**	**standard error**	**pRIPK3**	n=1	n=2	n=3	n=4	**Mean**	**standard deviation**	**standard error**
**control**	1.0	1.0	1.0		**1.0**	**0.0**	**0.0**	**control**	1.0	1.0	1.0		**1.0**	**0.0**	**0.0**
**UDCA 6h**	1.6	1.3	0.7		**1.2**	**0.5**	**0.3**	**UDCA 6h**	1.3	1.0	0.8		**1.1**	**0.3**	**0.2**
**UDCA 24 h**	1.5	1.6	0.7		**1.3**	**0.5**	**0.3**	**UDCA 24 h**	3.5	4.6	1.7		**3.2**	**1.5**	**0.8**
**control**	1.0	1.0	1.0	1.0	**1.0**	**0.0**	**0.0**	**control**	1.0	1.0	1.0	1.0	**1.0**	**0.0**	**0.0**
**TUDCA 6h**	2.2	0.9	1.6	0.5	**1.3**	**0.8**	**0.4**	**TUDCA 6h**	1.6	1.0	1.0	0.9	**1.1**	**0.3**	**0.2**
**TUDCA 24h**	2.1	1.2	2.1	0.7	**1.5**	**0.7**	**0.4**	**TUDCA 24h**	2.0	2.7	2.4	0.4	**1.9**	**1.0**	**0.5**
**control**	1.0	1.0	1.0	1.0	**1.0**	**0.0**	**0.0**	**control**	1.0	1.0	1.0	1.0	**1.0**	**0.0**	**0.0**
**GUDCA 6h**	1.3	0.5	0.4	1.1	**0.8**	**0.5**	**0.2**	**GUDCA 6h**	1.1	0.5	1.4	1.2	**1.0**	**0.4**	**0.2**
**GUDCA 24h**		1.2	0.6	0.6	**0.8**	**0.4**	**0.2**	**GUDCA 24h**	4.8	2.0	2.5	0.2	**2.4**	**1.9**	**0.9**

Figure 3F

**condition measured**	**PI* H33342**	**PI PI**	**H33342 H33342**	**H33342 PI**	**total cells**	**% PI positive**
**native**	64	149	1740	128	1761	8.5
**CA**	19	79	1097	14	1162	6,8
**RIPK3[Ser199Ala]**	8156	9335	51788	8441	52682	17,7
**RIPK3 [Ser199Asp][Ser227Asp]**	0	23801	5422	2246	26977	88,2
**RIPK3 + CA**	16	7949	3694	1484	10159	78,2
**RIPK3**	5462	8891	11832	6040	14683	60,6
**group**	PI-channel as mask		H33342-channel as mask			

**condition measured**	**total cells**	**% PI positive**	**% PI negative**	**% PI positive**	**% PI negative**	**amount positive**	**amount negativ**
**native**	1761	8.5	91.5	0.085	0.915	150	1611
**CA**	1162	6.8	93.2	0.068	0.932	79	1083
**RIPK3[Ser199Ala]**	52682	17.7	82.3	0.177	0.823	9325	43357
**RIPK3 [Ser199Asp][Ser227Asp]**	26977	88.2	11.8	0.882	0.118	23794	3183
**RIPK3 + CA**	10159	78.2	21.8	0.782	0.218	7944	2215
**RIPK3**	14683	60.6	39.4	0.606	0.394	8898	5785
**CONTROL* (summe CA+nativ)**	2923	7.8	92.2	0.078	0.922	229	2694

* z-test between native and CA was not significant --> put the two groups together to reduce the conditions

Figure 4A			
	control	RIPK3	RIPK3 + CA
	1.91	0.76	8.10
	0.73	0.80	4.96
	0.73	8.41	5.29
	2.30	2.86	5.05
	1.03	2.69	4.78
	0.95	3.98	4.27
	0.84	3.96	7.25
	1.16	3.72	7.58
	1.15	3.75	7.34
	1.54	4.54	7.18
	1.35	4.20	6.77
	1.02	5.41	7.99
	1.19	5.59	
	1.04	3.51	
	1.10	4.10	
	1.13	3.62	
	1.03	3.93	
	4.50	3.95	
	4.45	4.96	
	4.23	5.03	
	4.46	5.33	
	3.94	5.50	
	3.73	5.52	
	4.89	5.31	
	4.76	5.56	
	5.46	9.11	
		8.94	
		9.06	
		9.58	
		8.89	
		8.92	
**mean**	**2.33**	**5.21**	**6.38**
**standard deviation**	**1.66**	**2.39**	**1.39**
**standard error**	**0.33**	**0.43**	**0.40**

Figure 4B				
	RIPK3	[S199D]	[S227D]	[S199/227D]
	3.04	6.94	5.69	11.78
	3.43	5.83	5.97	10.13
	3.84	5.92	6.41	11.05
	3.47	5.59	5.93	11.13
	3.75	5.81	6.33	10.61
	3.94	6.16	6.88	9.63
	3.93	5.94	4.76	6.96
	3.95	5.30	4.48	7.76
	4.96	5.25	5.32	5.87
	5.03	5.17	4.75	6.02
	5.33	5.90	5.24	6.12
	5.50	5.01	4.94	5.94
	5.52	6.16	8.39	
	5.31	7.11	6.43	
	5.56	7.16	7.08	
	7.30	6.10	6.42	
	6.62	5.86	5.71	
	7.09	5.58	6.61	
	6.86			
	6.68			
	6.24			
**mean**	**5.11**	**5.93**	**5.96**	**8.58**
**standard deviation**	**1.34**	**0.62**	**0.98**	**2.35**
**standard error**	**0.29**	**0.15**	**0.23**	**0.68**

Figure 4C

RIPK3 transfection	no	yes	no	yes	yes	yes	no	yes	yes	yes	no	yes	yes	yes	no	yes	yes	yes
**Stimulation**	**no**	**no**	**CA**	**CA**	**GCA**	**TCA**	**CDCA**	**CDCA**	**GCDCA**	**TCDCA**	**UDCA**	**UDCA**	**GUDCA**	**TUDCA**	**LCA**	**LCA**	**GLCA**	**TLCA**
	0,12	2,83	-0,30	1,14	1,70	1,39	-0,98	3,47	0,98	1,05	-0,07	1,44	0,85	0,87	-0,40	1,86	0,62	1,05
	-0,18	1,88	0,19	1,23	1,20	0,57	-0,93	2,48	1,55	0,83	-0,30	1,43	1,14	0,68	-0,25	1,47	-0,99	0,43
	0,14	2,33	0,46	1,40	1,41	0,96	0,53	2,61	1,57	0,59	0,16	1,36	0,99	0,74	0,37	1,93	1,59	0,58
	-0,08	1,14	0,41	1,18	1,61	0,66	-0,22	2,51	0,91	1,09	0,29	1,47	0,81	0,69	0,29	1,23	1,15	0,80
	0,49	2,10	-0,85	1,27	1,39	0,70	0,98	1,89	0,90	0,82	-0,06	1,22	0,68	0,50		1,20	0,46	0,17
	-0,69	2,12	0,10	1,28	1,12	1,55	0,62	2,10	0,88	0,91	0,00	1,33	1,01	0,24		0,32	0,81	0,39
	0,09	2,06		1,78		0,98		1,98	0,87	0,72		1,36	0,52	0,67		1,13	0,36	0,35
	0,11	1,61		0,73						1,69								
	0,10	2,37		1,43						1,98								
	0,34	1,87		1,65						2,28								
	-0,13	2,42		2,29						2,59								
	-0,32	1,24		2,74														
	-0,03	0,96		3,82														
	0,04	0,76		2,76														
	0,09	0,81		2,46														
	-0,11	1,56		2,43														
	0,25	1,18		1,83														
	0,21	0,35		2,63														
	-0,22	0,82		1,84														
	-0,25	2,00		1,79														
	-0,02	1,72		1,27														
	-0,03	1,96		1,29														
	0,04	2,13		1,41														
	0,13	2,36		2,43														
	-0,08	1,21		2,49														
	0,25	0,74		2,55														
	-0,30	0,70		2,32														
	-0,02	0,72																
	-0,62	0,84																
	0,39	2,93																
	0,08	2,89																
	0,15	2,84																
	0,17	3,11																
	-0,18																	
	-0,05																	
	0,04																	
	-0,01																	
	0,00																	
	-0,16																	
	0,16																	
**mean**	**0,00**	**1,72**	**0,00**	**1,91**	**1,41**	**0,97**	**0,00**	**2,43**	**1,09**	**1,32**	**0,00**	**1,37**	**0,86**	**0,63**	**0,00**	**1,31**	**0,57**	
**standard deviation**	**0,24**	**0,78**	**0,50**	**0,70**	**0,23**	**0,37**	**0,84**	**0,54**	**0,32**	**0,69**	**0,20**	**0,09**	**0,21**	**0,21**	**0,38**	**0,54**	**0,81**	
**standard error**	**0,04**	**0,14**	**0,20**	**0,14**	**0,09**	**0,14**	**0,34**	**0,20**	**0,12**	**0,21**	**0,08**	**0,03**	**0,08**	**0,08**	**0,19**	**0,20**	**0,31**	
**amount**	**40**	**33**	**6**	**27**	**6**	**7**	**6**	**7**	**7**	**11**	**6**	**7**	**7**	**7**	**4**	**7**	**7**	

Figure 4D								
	n=1	n=2	n=3	n=4	n=5	mean	standard deviation	standard error
control	1.00	1.00	1.00	1.00	1.00	1.00	0.00	0.00
RIPK3-pcDNA	4.84	5.28	8.42	2.49	1.34	4.48	2.75	1.59
CA	15.10	9.72	16.73	2.66	1.82	9.21	6.87	3.97
CDCA	7.45	4.55	7.72	1.60	1.03	4.47	3.14	1.82
UDCA	16.47	20.04	32.04	2.23	5.23	15.20	12.00	6.93
LCA	3.80	6.98	15.33	2.27	0.84	5.84	5.77	3.33

Figure S1

	RIPK1		pRIPK1		RIPK3		pRIPK3		MLKL		pMLKL	
**TBZ**	-	+	-	+	-	+	-	+	-	+	-	+
**pHep**	1.0	91.1	1.0	25.9	0.0	0.0	0.0	0.0	1.0	0.8	1.0	3.1
	1.0	14.4	1.0	1.6	0.0	0.0	0.0	0.0	1.0	0.6	1.0	3.4
					0.0	0.0	0.0	0.0				
					0.0	0.0						
**pMac**	1.0	1.7	1.0	0.9	1.0	0.5	1.0	0.6	1.0	1.0	1.0	0.5
	1.0	2.2	1.0	5.3	1.0	0.8	1.0	1.2	1.0	0.7	1.0	0.7
					1.0	1.2	1.0	0.7				
					1.0	0.9						

Figure S2

	Blot	Gel	Coomassie Ratio	Blot Ratio	Coomassie*Blot
**control**	11294.217	128031.746	1	1	1.0
**Aza 24h**	20508.229	142035.059	0.901409461	1.815816803	1.6
**CM 24h**	20809.894	157925.423	0.810710167	1.842526489	1.5

*information: ultra luminol, ECL standard 1 min

Figure S5

	6h_1	
	max Lvse	Medium
	1.2	0.1
	0.9	0.1
	1.3	0.1
	0.9	0.1
	0.8	0.1
	1.4	0.1
	1.4	0.1
	1.9	0.1
mean	1.2	0.1

	6h_2	
	max Lvse	Medium
	1.9	0.1
	1.9	0.1
	2.0	0.1
	1.9	0.1
	2.0	0.1
	2.2	0.1
	1.9	0.1
	2.0	0.1
mean	2.0	0.1

	24h_1	
	max Lvse	Medium
	1.4	0.1
	1.3	0.1
	1.3	0.1
	1.6	0.1
mean	1.4	0.1

	24h_2	
	max Lvse	Medium
	2.8	0.1
	2.7	0.1
	3.3	0.1
	2.8	0.1
	3.3	0.1
	2.8	0.1
	2.8	0.1
	2.6	0.1
mean	2.9	0.1

raw data (6h)

	raw data	6h_1				6h_2			
	[uM]	n=1	n=2	n=3	n=4	n=1	n=2	n=3	n=4
LCA	50	0.4	0.4	0.4	0.4	0.5	0.5	0.7	0.4
	10	0.2	0.2	0.2	0.4	0.2	0.3	0.3	0.2
	2	0.3	0.2	0.2	0.3	0.2	0.2	0.4	0.2
	0.4	0.2	0.2		0.3	0.2	0.3	0.2	0.4
	0.08	0.2	0.3	0.2	0.2	0.3	0.4	0.3	0.3
	0.016	0.2	0.3	0.2	0.2	0.3	0.3	0.3	0.3
	0.0032	0.2	0.3	0.2	0.3	0.3	0.3	0.3	0.3
	0.00064	0.2	0.3	0.2	0.2	0.3	0.3	0.3	0.3
	0	0.2	0.3	0.3	0.2	0.2	0.3	0.3	0.3
	25	0.3	0.2	0.2	0.3	0.3	0.3	0.3	0.3
	5	0.2	0.2	0.3	0.2	0.3	0.4	0.4	0.3
	0.16	0.3	0.2	0.2	0.2	0.3	0.3	0.4	0.2
	[uM]	n=1	n=2	n=3	n=4	n=1	n=2	n=3	n=4
GLCA	50	0.4	0.4	0.3	0.4				
	10	0.3	0.2	0.2	0.3				
	2	0.3	0.2	0.2	0.2	0.2	0.2	0.3	0.2
	0.4	0.3	0.2	0.3	0.2	0.3	0.3	0.3	0.4
	0.08	0.2	0.2	0.3	0.2	0.3	0.3	0.4	0.4
	0.016	0.2	0.3	0.2	0.2	0.3	0.3	0.3	0.2
	0.0032	0.3	0.2	0.2	0.2	0.3	0.3	0.2	0.2
	0.00064	0.2	0.2	0.2	0.2	0.3	0.3	0.4	0.3
	0	0.2	0.3	0.2	0.3	0.3	0.3	0.3	0.3
	25	0.3	0.3	0.3	0.3				
	5	0.2	0.2	0.2	0.2	0.2	0.2	0.3	0.3
	0.16	0.2	0.2	0.2	0.2	0.3	0.3	0.3	0.4
	[uM]	n=1	n=2	n=3	n=4	n=1	n=2	n=3	n=4
TLCA	50	0.3	0.2	0.2	0.3	0.4	0.3	0.3	0.3
	10	0.2	0.2	0.2	0.3	0.3	0.4	0.3	0.2
	2	0.3	0.2	0.2	0.3	0.2	0.4	0.2	0.3
	0.4	0.3	0.2	0.2	0.3	0.3	0.3	0.3	0.3
	0.08	0.2	0.3	0.3	0.2	0.2	0.3	0.3	0.2
	0.016	0.2	0.3	0.3	0.3	0.3	0.3	0.3	0.3
	0.0032	0.3	0.3	0.3	0.3	0.3	0.3	0.3	0.4
	0.00064	0.3	0.3	0.3	0.3	0.2	0.3	0.3	0.8
	0	0.3	0.3	0.3	0.4	0.2	0.5	0.3	0.4
	25	0.2	0.3	0.3	0.3	0.3	0.3	0.3	0.4
	5	0.3	0.3	0.3	0.3	0.4	0.3	0.3	0.3
	0.16	0.3	0.3	0.3	0.3	0.4	0.4	0.3	0.3

-Medium (6h)

	-Medium	6h_1				6h_2			
	[uM]	n=1	n=2	n=3	n=4	n=1	n=2	n=3	n=4
LCA	50	0.4	0.4	0.3	0.3	0.4	0.4	0.6	0.3
	10	0.2	0.1	0.2	0.3	0.2	0.2	0.3	0.2
	2	0.2	0.2	0.2	0.2	0.1	0.2	0.3	0.2
	0.4	0.1	0.2	-0.1	0.2	0.2	0.2	0.2	0.4
	0.08	0.1	0.2	0.2	0.2	0.2	0.3	0.3	0.2
	0.016	0.1	0.2	0.1	0.2	0.2	0.2	0.3	0.2
	0.0032	0.1	0.2	0.2	0.2	0.2	0.2	0.2	0.2
	0.00064	0.2	0.2	0.2	0.2	0.2	0.2	0.2	0.2
	0	0.2	0.2	0.2	0.2	0.2	0.2	0.2	0.2
	25	0.2	0.2	0.2	0.2	0.2	0.2	0.2	0.3
	5	0.2	0.2	0.2	0.2	0.3	0.3	0.3	0.3
	0.16	0.2	0.2	0.2	0.2	0.2	0.3	0.3	0.1
	[uM]	n=1	n=2	n=3	n=4	n=1	n=2	n=3	n=4
GLCA	50	0.3	0.3	0.2	0.4				
	10	0.2	0.1	0.2	0.2				
	2	0.2	0.1	0.1	0.2	0.1	0.1	0.2	0.2
	0.4	0.2	0.1	0.3	0.2	0.2	0.2	0.2	0.3
	0.08	0.2	0.2	0.2	0.1	0.2	0.2	0.3	0.3
	0.016	0.2	0.2	0.2	0.1	0.3	0.2	0.3	0.2
	0.0032	0.2	0.2	0.2	0.1	0.2	0.2	0.2	0.2
	0.00064	0.2	0.2	0.2	0.1	0.2	0.2	0.3	0.2
	0	0.2	0.2	0.1	0.2	0.2	0.2	0.3	0.2
	25	0.2	0.2	0.2	0.2				
	5	0.2	0.2	0.2	0.2	0.2	0.2	0.2	0.2
	0.16	0.1	0.2	0.2	0.1	0.2	0.3	0.2	0.3
	[uM]	n=1	n=2	n=3	n=4	n=1	n=2	n=3	n=4
TLCA	50	0.2	0.2	0.2	0.2	0.3	0.3	0.2	0.2
	10	0.2	0.2	0.1	0.2	0.2	0.4	0.2	0.2
	2	0.2	0.2	0.2	0.2	0.2	0.3	0.2	0.2
	0.4	0.2	0.1	0.1	0.2	0.2	0.3	0.2	0.2
	0.08	0.1	0.2	0.2	0.2	0.2	0.3	0.2	0.1
	0.016	0.2	0.2	0.2	0.3	0.2	0.3	0.2	0.2
	0.0032	0.2	0.2	0.2	0.2	0.2	0.3	0.2	0.3
	0.00064	0.3	0.2	0.2	0.2	0.1	0.3	0.3	0.7
	0	0.2	0.2	0.2	0.3	0.2	0.4	0.2	0.4
	25	0.2	0.2	0.2	0.2	0.2	0.2	0.3	0.3
	5	0.2	0.2	0.2	0.3	0.3	0.3	0.3	0.3
	0.16	0.2	0.2	0.3	0.3	0.3	0.3	0.3	0.2

% (6h)

	%	6h_1				6h_2			
	[uM]	n=1	n=2	n=3	n=4	n=1	n=2	n=3	n=4
LCA	50	29.3	30.6	26.4	27.1	22.1	21.1	29.8	16.1
	10	12.8	11.7	15.0	25.2	9.0	11.4	13.1	8.5
	2	16.0	13.9	15.4	18.5	6.8	7.8	15.6	8.8
	0.4	11.7	14.3	-5.1	17.1	9.2	10.9	8.3	18.6
	0.08	11.7	15.5	14.9	15.2	11.5	16.0	13.3	10.7
	0.016	11.1	16.2	11.7	15.2	9.6	12.2	14.0	10.8
	0.0032	11.7	18.0	14.4	20.1	10.1	9.8	12.2	12.2
	0.00064	13.6	17.8	13.1	15.2	11.8	11.6	12.0	10.7
	0	13.1	16.4	17.0	12.6	9.0	12.2	12.5	10.6
	25	16.8	14.6	14.7	16.6	10.4	9.6	11.1	13.9
	5	15.0	15.1	18.8	13.1	13.1	14.6	14.5	13.1
	0.16	15.9	15.0	14.6	13.8	9.5	13.6	14.8	6.2
	[uM]	n=1	n=2	n=3	n=4	n=1	n=2	n=3	n=4
GLCA	50	25.6	26.6	18.5	29.2				
	10	16.3	9.8	12.7	15.6				
	2	16.0	12.0	11.7	13.3	6.8	7.3	11.1	8.6
	0.4	15.8	10.8	21.6	15.1	9.4	10.2	9.5	16.9
	0.08	14.5	12.8	16.6	12.0	10.3	12.6	15.7	15.1
	0.016	15.0	16.7	14.5	11.4	13.2	11.9	13.3	8.6
	0.0032	15.9	12.7	14.0	9.9	10.1	11.9	9.1	8.7
	0.00064	13.6	13.9	14.8	8.0	11.4	10.9	15.9	10.4
	0	14.5	16.0	11.6	18.9	10.4	9.5	13.7	9.6
	25	17.6	17.1	19.2	19.5				
	5	13.8	13.0	13.9	12.9	8.6	7.8	10.3	9.8
	0.16	11.9	15.1	12.8	11.2	11.4	13.6	11.7	15.7
	[uM]	n=1	n=2	n=3	n=4	n=1	n=2	n=3	n=4
TLCA	50	16.4	12.5	13.0	18.5	14.7	12.9	10.5	10.3
	10	13.1	12.9	10.8	18.1	9.2	17.8	9.6	8.0
	2	16.6	14.0	15.0	16.9	9.1	15.2	8.4	12.2
	0.4	16.3	11.7	11.2	20.5	10.8	13.8	10.7	9.9
	0.08	12.1	16.8	16.8	13.9	8.4	13.8	12.1	7.1
	0.016	14.3	20.0	17.2	21.3	11.1	13.5	9.4	12.5
	0.0032	17.9	16.6	18.5	18.8	11.3	12.9	12.6	15.2
	0.00064	22.1	15.7	18.7	18.0	6.0	13.8	12.7	35.4
	0	18.7	19.4	19.5	26.2	9.0	21.6	9.7	17.8
	25	14.5	17.4	17.7	15.9	10.9	12.6	13.5	16.0
	5	18.9	19.5	18.9	20.8	15.6	14.0	13.9	14.1
	0.16	19.2	18.9	21.9	20.9	17.4	17.6	12.6	10.4

- control (6h)

	- control	6h_1				6h_2						
	[uM]	n=1	n=2	n=3	n=4	n=1	n=2	n=3	n=4	mean	standard deviation	standard error
LCA	50	14.5	15.8	11.6	12.4	11.1	10.0	18.8	5.1			
	10	-2.0	-3.0	0.2	10.5	-2.0	0.3	2.0	-2.5	0.4	4.4	1.6
	2	1.2	-0.9	0.6	3.7	-4.3	-3.2	4.5	-2.2	-0.1	3.2	1.1
	0.4	-3.0	-0.5	-19.9	2.3	-1.8	-0.1	-2.8	7.6	-2.3	7.9	2.8
	0.08	-3.1	0.7	0.1	0.4	0.4	4.9	2.2	-0.4	0.7	2.3	0.8
	0.016	-3.7	1.4	-3.0	0.4	-1.4	1.2	2.9	-0.2	-0.3	2.3	0.8
	0.0032	-3.1	3.2	-0.4	5.3	-0.9	-1.2	1.1	1.1	0.6	2.7	0.9
	0.00064	-1.2	3.0	-1.6	0.4	0.7	0.5	1.0	-0.3	0.3	1.5	0.5
	0											
	25	2.1	-0.2	-0.1	1.8	-0.6	-1.4	0.1	2.8	0.6	1.5	0.5
	5	0.2	0.3	4.0	-1.7	2.0	3.5	3.4	2.0	1.7	2.0	0.7
	0.16	1.1	0.2	-0.2	-1.0	-1.5	2.5	3.8	-4.9	0.0	2.6	0.9
	[uM]	n=1	n=2	n=3	n=4	n=1	n=2	n=3	n=4	mean	standard deviation	standard error
GLCA	50	10.3	11.3	3.2	13.9					9.7	4.6	1.6
	10	1.0	-5.5	-2.5	0.3					-1.7	3.0	1.1
	2	0.8	-3.3	-3.6	-2.0	-4.0	-3.5	0.2	-2.2	-2.2	1.8	0.6
	0.4	0.5	-4.5	6.4	-0.1	-1.4	-0.6	-1.3	6.1	0.6	3.8	1.3
	0.08	-0.8	-2.4	1.3	-3.3	-0.5	1.7	4.9	4.3	0.6	3.0	1.0
	0.016	-0.3	1.4	-0.8	-3.8	2.4	1.0	2.5	-2.2	0.0	2.2	0.8
	0.0032	0.6	-2.5	-1.2	-5.3	-0.7	1.0	-1.7	-2.1	-1.5	2.0	0.7
	0.00064	-1.7	-1.4	-0.5	-7.3	0.6	0.1	5.1	-0.4	-0.7	3.4	1.2
	0											
	25	2.3	1.8	4.0	4.2					3.1	1.2	0.4
	5	-1.5	-2.3	-1.4	-2.4	-2.2	-3.0	-0.5	-1.0	-1.8	0.8	0.3
	0.16	-3.4	-0.1	-2.4	-4.1	0.5	2.8	0.9	4.9	-0.1	3.1	1.1
	[uM]	n=1	n=2	n=3	n=4	n=1	n=2	n=3	n=4	mean	standard deviation	standard error
TLCA	50	-4.5	-8.5	-8.0	-2.4	0.1	-1.7	-4.0	-4.2	-4.1	2.9	1.0
	10	-7.9	-8.0	-10.2	-2.9	-5.3	3.3	-4.9	-6.5	-5.3	4.1	1.5
	2	-4.3	-7.0	-5.9	-4.0	-5.4	0.7	-6.1	-2.3	-4.3	2.5	0.9
	0.4	-4.7	-9.2	-9.7	-0.5	-3.8	-0.8	-3.9	-4.6	-4.6	3.4	1.2
	0.08	-8.9	-4.1	-4.1	-7.1	-6.1	-0.7	-2.4	-7.4	-5.1	2.7	1.0
	0.016	-6.6	-1.0	-3.8	0.3	-3.4	-1.1	-5.1	-2.0	-2.8	2.3	0.8
	0.0032	-3.0	-4.3	-2.4	-2.1	-3.3	-1.6	-2.0	0.6	-2.3	1.5	0.5
	0.00064	1.2	-5.2	-2.3	-2.9	-8.5	-0.8	-1.8	20.8	0.1	8.9	3.1
	0											
	25	-6.4	-3.5	-3.3	-5.1	-3.6	-2.0	-1.1	1.4	-2.9	2.4	0.9
	5	-2.0	-1.5	-2.0	-0.1	1.1	-0.5	-0.7	-0.4	-0.8	1.1	0.4
	0.16	-1.8	-2.0	0.9	-0.1	2.9	3.1	-1.9	-4.1	-0.4	2.5	0.9

raw data (24h)

	raw data	24h_1				24h_2			
	[uM]	n=1	n=2	n=3	n=4	n=1	n=2	n=3	n=4
LCA	50	0.7	0.8	0.8	0.8	1.6	1.7	1.9	1.7
	10	0.3	0.3	0.3	0.3	0.9	0.7	0.7	0.7
	2	0.3	0.3	0.3	0.3	0.3	0.3	0.2	0.3
	0.4	0.3	0.3	0.3	0.3	0.3	0.3	0.3	0.3
	0.08	0.3	0.3	0.3	0.3	0.3	0.3	0.3	0.3
	0.016	0.3	0.3	0.3	0.3	0.3	0.4	0.4	0.4
	0.0032	0.3	0.3	0.3	0.3	0.2	0.3	0.3	0.4
	0.00064	0.3	0.3	0.2	0.3	0.3	0.3	0.4	0.4
	0	0.2	0.3	0.3	0.3	0.3	0.3	0.3	0.3
	25	0.3	0.4	0.4	0.4	1.7	1.3	1.0	1.4
	5	0.3	0.3	0.3	0.3	0.4	0.3	0.4	0.4
	0.16	0.3	0.2	0.3	0.3	0.4	0.4	0.4	0.6
	[uM]	n=1	n=2	n=3	n=4	n=1	n=2	n=3	n=4
GLCA	50	0.7	0.7	0.8	0.8	1.2	1.4	1.2	1.2
	10	0.2	0.3	0.2	0.3	0.4	0.3	0.3	0.2
	2	0.3	0.3	0.3	0.3	0.2	0.3	0.2	0.2
	0.4	0.3	0.3	0.3	0.3	0.2	0.3	0.3	0.3
	0.08	0.3	0.3	0.2	0.3	0.3	0.3	0.3	0.3
	0.016	0.3	0.3	0.3	0.3	0.4	0.4	0.4	0.4
	0.0032	0.3	0.3	0.3	0.3	0.3	0.3	0.3	0.2
	0.00064	0.4	0.3	0.3	0.2	0.3	0.3	0.3	0.3
	0	0.3	0.2	0.4	0.3	0.4	0.3	0.3	0.3
	25	0.5	0.3	0.5	0.5	1.2	1.3	1.2	1.2
	5	0.3	0.3	0.2	0.2	0.4	0.4	0.5	0.5
	0.16	0.3	0.3	0.3	0.3	0.4	0.4	0.4	0.4
	[uM]	n=1	n=2	n=3	n=4	n=1	n=2	n=3	n=4
TLCA	50	0.3	0.3	0.3	0.3	0.4	0.3	0.3	0.3
	10	0.3	0.3	0.3	0.3	0.3	0.2	0.3	0.3
	2	0.2	0.3	0.2	0.3	0.3	0.3	0.3	0.3
	0.4	0.3	0.3	0.3	0.3	0.3	0.3	0.3	0.3
	0.08	0.3	0.3	0.3	0.3	0.2	0.3	0.2	0.2
	0.016	0.3	0.3	0.3	0.3	0.3	0.3	0.2	0.3
	0.0032	0.3	0.4	0.3	0.3	0.3	0.2	0.3	0.3
	0.00064	0.3	0.3	0.4	0.3	0.3	0.2	0.2	0.2
	0	0.3	0.4	0.3	0.3	0.2	0.2	0.3	0.3
	25	0.3	0.3	0.3	0.3	0.3	0.2	0.2	0.3
	5	0.3	0.2	0.3	0.3	0.3	0.2	0.3	0.3
	0.16	0.4	0.4	0.3	0.3	0.3	0.2	0.3	0.2

-Medium (24h)

	-Medium	24h_1				24h_2			
	[uM]	n=1	n=2	n=3	n=4	n=1	n=2	n=3	n=4
LCA	50	0.6	0.8	0.7	0.8	1.5	1.6	1.8	1.6
	10	0.2	0.3	0.2	0.3	0.8	0.7	0.6	0.6
	2	0.3	0.3	0.2	0.2	0.2	0.2	0.2	0.2
	0.4	0.2	0.2	0.2	0.2	0.3	0.2	0.2	0.2
	0.08	0.2	0.2	0.2	0.2	0.2	0.2	0.3	0.2
	0.016	0.2	0.2	0.2	0.2	0.3	0.3	0.4	0.3
	0.0032	0.2	0.3	0.2	0.3	0.2	0.2	0.2	0.3
	0.00064	0.2	0.2	0.2	0.3	0.3	0.3	0.3	0.3
	0	0.2	0.2	0.2	0.2	0.2	0.2	0.2	0.2
	25	0.3	0.3	0.3	0.3	1.6	1.2	1.0	1.3
	5	0.2	0.2	0.2	0.2	0.3	0.2	0.3	0.3
	0.16	0.2	0.2	0.3	0.2	0.3	0.3	0.3	0.5
	[uM]	n=1	n=2	n=3	n=4	n=1	n=2	n=3	n=4
GLCA	50	0.6	0.7	0.7	0.7	1.2	1.3	1.2	1.1
	10	0.2	0.2	0.2	0.2	0.3	0.3	0.2	0.2
	2	0.2	0.2	0.2	0.2	0.2	0.2	0.2	0.2
	0.4	0.2	0.2	0.2	0.2	0.2	0.2	0.2	0.2
	0.08	0.2	0.2	0.2	0.3	0.2	0.2	0.2	0.2
	0.016	0.2	0.2	0.2	0.2	0.3	0.3	0.3	0.3
	0.0032	0.2	0.3	0.2	0.2	0.2	0.2	0.2	0.2
	0.00064	0.3	0.2	0.3	0.2	0.2	0.2	0.3	0.3
	0	0.3	0.2	0.3	0.3	0.3	0.3	0.3	0.2
	25	0.4	0.3	0.4	0.5	1.2	1.2	1.2	1.1
	5	0.3	0.2	0.2	0.2	0.3	0.3	0.4	0.4
	0.16	0.2	0.2	0.2	0.2	0.3	0.3	0.4	0.4
	[uM]	n=1	n=2	n=3	n=4	n=1	n=2	n=3	n=4
TLCA	50	0.2	0.2	0.2	0.2	0.3	0.2	0.2	0.2
	10	0.2	0.2	0.2	0.2	0.3	0.1	0.2	0.2
	2	0.2	0.2	0.2	0.3	0.2	0.2	0.2	0.2
	0.4	0.2	0.2	0.2	0.2	0.3	0.2	0.2	0.2
	0.08	0.2	0.2	0.2	0.3	0.2	0.2	0.2	0.2
	0.016	0.3	0.2	0.2	0.3	0.2	0.2	0.1	0.2
	0.0032	0.3	0.3	0.2	0.3	0.3	0.1	0.3	0.2
	0.00064	0.3	0.3	0.3	0.3	0.2	0.1	0.1	0.2
	0	0.2	0.3	0.3	0.2	0.2	0.2	0.2	0.2
	25	0.2	0.2	0.3	0.2	0.2	0.2	0.1	0.2
	5	0.2	0.2	0.2	0.2	0.2	0.2	0.3	0.2
	0.16	0.3	0.3	0.2	0.2	0.2	0.2	0.2	0.2

% (24h)

	%	24h_1				24h_2			
	[uM]	n=1	n=2	n=3	n=4	n=1	n=2	n=3	n=4
LCA	50	44.7	54.7	50.4	54.6	53.4	56.1	62.1	55.0
	10	17.8	19.9	16.9	18.0	28.3	22.6	20.6	21.9
	2	18.4	19.8	16.9	15.7	7.4	7.5	6.1	7.0
	0.4	16.7	17.5	16.2	15.6	9.5	7.2	8.7	6.6
	0.08	15.8	16.5	16.3	14.2	7.4	8.5	9.0	8.5
	0.016	14.4	16.2	16.5	17.1	9.6	10.0	12.3	11.2
	0.0032	14.2	18.3	16.5	18.9	6.0	7.5	8.5	9.8
	0.00064	14.5	13.7	12.2	18.4	9.5	9.5	10.2	10.1
	0	13.1	14.0	15.7	16.3	7.2	8.3	8.5	8.6
	25	19.8	20.6	20.6	21.0	56.2	43.2	33.9	45.4
	5	17.5	14.7	15.7	16.2	11.2	8.5	10.1	10.3
	0.16	14.0	11.7	18.4	16.7	11.8	10.5	11.8	17.4
	[uM]	n=1	n=2	n=3	n=4	n=1	n=2	n=3	n=4
GLCA	50	42.8	48.4	52.9	51.7	40.4	45.8	40.1	39.6
	10	12.9	14.7	12.5	16.0	10.9	9.5	6.5	5.9
	2	16.2	13.4	15.1	16.5	6.1	7.9	5.9	5.9
	0.4	14.6	16.5	15.0	17.0	6.2	7.0	6.7	8.0
	0.08	14.2	17.7	10.8	19.2	8.0	8.2	6.9	7.6
	0.016	17.7	17.6	14.1	13.2	9.8	10.4	11.5	11.2
	0.0032	17.4	18.5	15.3	16.7	7.8	7.8	8.4	5.8
	0.00064	22.7	17.2	19.3	13.1	7.8	8.0	9.5	9.2
	0	18.3	11.4	21.6	18.7	11.5	8.9	8.7	7.6
	25	30.0	20.3	28.5	33.0	40.7	41.3	40.2	39.9
	5	20.2	16.1	12.6	11.9	10.6	10.1	13.7	13.4
	0.16	17.7	16.5	16.0	15.9	10.2	10.9	12.7	12.9
	[uM]	n=1	n=2	n=3	n=4	n=1	n=2	n=3	n=4
TLCA	50	14.4	14.9	15.8	13.7	9.8	7.6	7.6	6.7
	10	14.9	14.5	16.9	14.3	9.5	5.1	7.1	6.7
	2	11.9	13.7	11.7	19.1	6.8	7.0	6.4	7.3
	0.4	13.4	14.7	15.1	15.2	9.6	7.7	8.5	6.6
	0.08	17.8	15.8	17.6	18.1	6.1	7.6	6.1	6.1
	0.016	18.1	15.4	14.9	19.0	7.1	6.7	4.7	6.5
	0.0032	17.9	21.3	14.6	19.3	9.0	5.2	9.7	6.8
	0.00064	20.2	18.7	20.9	19.2	7.8	4.2	4.4	5.9
	0	17.7	23.0	18.2	16.4	6.2	5.4	6.7	7.2
	25	15.7	13.7	19.1	13.9	6.7	5.7	4.4	7.1
	5	13.6	11.2	17.0	15.2	6.9	5.7	8.9	7.5
	0.16	21.5	24.2	17.5	16.7	8.3	5.6	7.1	5.6

- control (24h)

	- control	24h_1				24h_2						
	[uM]	n=1	n=2	n=3	n=4	n=1	n=2	n=3	n=4	mean	standard deviation	standard error
LCA	50	30.0	40.0	35.7	39.9	45.3	47.9	53.9	46.8	42.4	7.6	2.7
	10	3.1	5.2	2.2	3.3	20.2	14.4	12.4	13.7	9.3	6.7	2.4
	2	3.7	5.1	2.2	1.0	-0.8	-0.7	-2.1	-1.1	0.9	2.5	0.9
	0.4	2.0	2.8	1.5	0.9	1.4	-0.9	0.5	-1.5	0.8	1.4	0.5
	0.08	1.1	1.8	1.6	-0.5	-0.8	0.3	0.9	0.3	0.6	0.9	0.3
	0.016	-0.3	1.5	1.8	2.4	1.4	1.9	4.2	3.0	2.0	1.3	0.5
	0.0032	-0.5	3.6	1.8	4.2	-2.1	-0.7	0.3	1.7	1.0	2.2	0.8
	0.00064	-0.2	-1.0	-2.5	3.7	1.3	1.3	2.0	1.9	0.8	2.0	0.7
	0											
	25	5.1	5.9	5.9	6.3	48.0	35.1	25.7	37.3	21.2	17.5	6.2
	5	2.8	0.0	1.0	1.5	3.0	0.3	1.9	2.1	1.6	1.1	0.4
	0.16	-0.7	-3.0	3.7	2.0	3.7	2.4	3.7	9.2	2.6	3.6	1.3
	[uM]	n=1	n=2	n=3	n=4	n=1	n=2	n=3	n=4	mean	standard deviation	standard error
GLCA	50	25.3	30.8	35.4	34.2	31.2	36.6	30.9	30.4	31.8	3.6	1.3
	10	-4.6	-2.8	-5.0	-1.6	1.7	0.3	-2.7	-3.3	-2.3	2.3	0.8
	2	-1.3	-4.1	-2.4	-1.0	-3.1	-1.3	-3.3	-3.3	-2.5	1.2	0.4
	0.4	-2.9	-1.1	-2.5	-0.6	-3.0	-2.2	-2.5	-1.2	-2.0	0.9	0.3
	0.08	-3.3	0.2	-6.7	1.7	-1.2	-1.0	-2.3	-1.6	-1.8	2.5	0.9
	0.016	0.2	0.1	-3.4	-4.3	0.6	1.2	2.4	2.0	-0.2	2.4	0.9
	0.0032	-0.1	1.0	-2.2	-0.8	-1.4	-1.4	-0.8	-3.4	-1.1	1.3	0.5
	0.00064	5.2	-0.3	1.8	-4.4	-1.4	-1.2	0.3	0.0	0.0	2.8	1.0
	0											
	25	12.5	2.8	11.0	15.5	31.5	32.1	31.0	30.7	20.9	11.7	4.1
	5	2.7	-1.4	-4.9	-5.6	1.4	0.9	4.5	4.2	0.2	3.9	1.4
	0.16	0.2	-1.1	-1.6	-1.6	1.0	1.7	3.5	3.7	0.7	2.1	0.8
	[uM]	n=1	n=2	n=3	n=4	n=1	n=2	n=3	n=4	mean	standard deviation	standard error
TLCA	50	-4.4	-3.9	-3.0	-5.2	3.5	1.3	1.2	0.3	-1.3	3.2	1.1
	10	-3.9	-4.3	-1.9	-4.5	3.1	-1.2	0.7	0.3	-1.5	2.7	1.0
	2	-6.9	-5.1	-7.1	0.3	0.5	0.6	0.1	1.0	-2.1	3.6	1.3
	0.4	-5.4	-4.1	-3.7	-3.6	3.2	1.4	2.1	0.2	-1.2	3.3	1.2
	0.08	-1.0	-3.0	-1.2	-0.7	-0.2	1.2	-0.2	-0.3	-0.7	1.2	0.4
	0.016	-0.7	-3.4	-3.9	0.2	0.7	0.3	-1.6	0.1	-1.0	1.8	0.6
	0.0032	-0.9	2.5	-4.2	0.5	2.6	-1.2	3.3	0.4	0.4	2.5	0.9
	0.00064	1.4	-0.1	2.1	0.4	1.5	-2.2	-2.0	-0.5	0.1	1.6	0.6
	0											
	25	-3.1	-5.1	0.3	-4.9	0.4	-0.7	-2.0	0.7	-1.8	2.4	0.8
	5	-5.2	-7.6	-1.9	-3.6	0.5	-0.6	2.6	1.2	-1.8	3.4	1.2
	0.16	2.7	5.4	-1.3	-2.1	2.0	-0.8	0.7	-0.7	0.7	2.5	0.9

Figure S6

	CA	TCA	GCA	CDCA	TCDCA	GCDCA	LCA	TLCA	GLCA	UDCA	TUDCA	GUDCA
control	n.d.	n.d.	n.d.	n.d.	n.d.	n.d.	n.d.	n.d.	n.d.	n.d.	n.d.	n.d.
control	n.d.	n.d.	n.d.	n.d.	n.d.	n.d.	n.d.	n.d.	n.d.	n.d.	n.d.	n.d.
control	n.d.	n.d.	n.d.	n.d.	n.d.	n.d.	n.d.	n.d.	n.d.	n.d.	n.d.	n.d.
control	n.d.	n.d.	n.d.	n.d.	n.d.	n.d.	n.d.	n.d.	n.d.	n.d.	n.d.	n.d.
control	n.d.	n.d.	n.d.	n.d.	n.d.	n.d.	n.d.	n.d.	n.d.	n.d.	n.d.	n.d.
CA	0.0572	n.d.	n.d.	0.0639	n.d.	n.d.	n.d.	n.d.	n.d.	n.d.	n.d.	n.d.
CA	0.0638	n.d.	n.d.	0.0609	n.d.	n.d.	n.d.	n.d.	n.d.	n.d.	n.d.	n.d.
CA	0.0575	n.d.	n.d.	0.0591	n.d.	n.d.	n.d.	n.d.	n.d.	n.d.	n.d.	n.d.
TCA	n.d.	0.144	n.d.	n.d.	n.d.	n.d.	n.d.	n.d.	n.d.	n.d.	n.d.	n.d.
TCA	n.d.	0.131	n.d.	n.d.	n.d.	n.d.	n.d.	n.d.	n.d.	n.d.	n.d.	n.d.
TCA	n.d.	0.142	n.d.	n.d.	n.d.	n.d.	n.d.	n.d.	n.d.	n.d.	n.d.	n.d.
GCA	n.d.	n.d.	0.0638	n.d.	n.d.	n.d.	n.d.	n.d.	n.d.	n.d.	n.d.	n.d.
GCA	n.d.	n.d.	0.0675	n.d.	n.d.	n.d.	n.d.	n.d.	n.d.	n.d.	n.d.	n.d.
GCA	n.d.	n.d.	0.0648	n.d.	n.d.	n.d.	n.d.	n.d.	n.d.	n.d.	n.d.	n.d.
CDCA	n.d.	n.d.	n.d.	0.371	n.d.	n.d.	n.d.	n.d.	n.d.	n.d.	n.d.	n.d.
CDCA	n.d.	n.d.	n.d.	0.33	n.d.	n.d.	n.d.	n.d.	n.d.	n.d.	n.d.	n.d.
CDCA	n.d.	n.d.	n.d.	0.296	n.d.	n.d.	n.d.	n.d.	n.d.	n.d.	n.d.	n.d.
TCDCA	n.d.	n.d.	n.d.	0.071	0.13	n.d.	n.d.	n.d.	n.d.	n.d.	n.d.	n.d.
TCDCA	n.d.	n.d.	n.d.	0.072	0.132	n.d.	n.d.	n.d.	n.d.	n.d.	n.d.	n.d.
TCDCA	n.d.	n.d.	n.d.	0.0625	0.114	n.d.	n.d.	n.d.	n.d.	n.d.	n.d.	n.d.
GCDCA	n.d.	n.d.	n.d.	0.0617	n.d.	0.0807	n.d.	n.d.	n.d.	n.d.	n.d.	n.d.
GCDCA	n.d.	n.d.	n.d.	0.0621	n.d.	0.0968	n.d.	n.d.	n.d.	n.d.	n.d.	n.d.
GCDCA	n.d.	n.d.	n.d.	0.061	n.d.	0.0866	n.d.	n.d.	n.d.	n.d.	n.d.	n.d.
LCA	n.d.	n.d.	n.d.	n.d.	n.d.	n.d.	0.298	n.d.	n.d.	n.d.	n.d.	n.d.
LCA	n.d.	n.d.	n.d.	n.d.	n.d.	n.d.	0.285	n.d.	n.d.	n.d.	n.d.	n.d.
LCA	n.d.	n.d.	n.d.	n.d.	n.d.	n.d.	0.277	n.d.	n.d.	n.d.	n.d.	n.d.
TLCA	n.d.	n.d.	n.d.	n.d.	n.d.	n.d.	n.d.	0.408	n.d.	n.d.	n.d.	n.d.
TLCA	n.d.	n.d.	n.d.	n.d.	n.d.	n.d.	n.d.	0.857	n.d.	n.d.	n.d.	n.d.
TLCA	n.d.	n.d.	n.d.	n.d.	n.d.	n.d.	n.d.	0.85	n.d.	n.d.	n.d.	n.d.
GLCA	n.d.	n.d.	n.d.	n.d.	n.d.	n.d.	n.d.	0.0771	0.124	n.d.	n.d.	n.d.
GLCA	n.d.	n.d.	n.d.	n.d.	n.d.	n.d.	n.d.	0.0737	0.167	n.d.	n.d.	n.d.
GLCA	n.d.	n.d.	n.d.	n.d.	n.d.	n.d.	n.d.	0.0736	0.174	n.d.	n.d.	n.d.
UDCA	n.d.	n.d.	n.d.	0.0588	n.d.	n.d.	n.d.	n.d.	n.d.	0.113	n.d.	n.d.
UDCA	n.d.	n.d.	n.d.	0.0608	n.d.	n.d.	n.d.	n.d.	n.d.	0.155	n.d.	n.d.
UDCA	n.d.	n.d.	n.d.	0.0617	n.d.	n.d.	n.d.	n.d.	n.d.	0.145	n.d.	n.d.
TUDCA	n.d.	n.d.	n.d.	n.d.	n.d.	n.d.	n.d.	n.d.	n.d.	0.0407	0.0453	n.d.
TUDCA	n.d.	n.d.	n.d.	n.d.	n.d.	n.d.	n.d.	n.d.	n.d.	0.04	0.0456	n.d.
TUDCA	n.d.	n.d.	n.d.	n.d.	n.d.	n.d.	n.d.	n.d.	n.d.	0.0385	0.0445	n.d.
GUDCA	n.d.	n.d.	n.d.	n.d.	n.d.	n.d.	n.d.	n.d.	n.d.	n.d.	n.d.	0.0463
GUDCA	n.d.	n.d.	n.d.	n.d.	n.d.	n.d.	n.d.	n.d.	n.d.	n.d.	n.d.	0.0446
GUDCA	n.d.	n.d.	n.d.	n.d.	n.d.	n.d.	n.d.	n.d.	n.d.	n.d.	n.d.	0.0538

mean values	CA	TCA	GCA	CDCA	TCDCA	GCDCA	LCA	TLCA	GLCA	UDCA	TUDCA	GUDCA
**control**	nd	nd	nd	nd	nd	nd	nd	nd	nd	nd	nd	nd
**CA**	0.060	nd	nd	0.061	nd	nd	nd	nd	nd	nd	nd	nd
**TCA**	nd	0.139	nd	nd	nd	nd	nd	nd	nd	nd	nd	nd
**GCA**	nd	nd	0.065	nd	nd	nd	nd	nd	nd	nd	nd	nd
**CDCA**	nd	nd	nd	0.332	nd	nd	nd	nd	nd	nd	nd	nd
**TCDCA**	nd	nd	nd	0.069	0.125	nd	nd	nd	nd	nd	nd	nd
**GCDCA**	nd	nd	nd	0.062	nd	0.088	nd	nd	nd	nd	nd	nd
**LCA**	nd	nd	nd	nd	nd	nd	0.287	nd	nd	nd	nd	nd
**TLCA**	nd	nd	nd	nd	nd	nd	nd	0.705	nd	nd	nd	nd
**GLCA**	nd	nd	nd	nd	nd	nd	nd	0.075	0.155	nd	nd	nd
**UDCA**	nd	nd	nd	nd	nd	nd	nd	nd	nd	0.138	nd	nd
**TUDCA**	nd	nd	nd	nd	nd	nd	nd	nd	nd	0.040	0.045	nd
**GUDCA**	nd	nd	nd	nd	nd	nd	nd	nd	nd	nd	nd	0.048

FigureS7B

CDCA

RIPK3	n=1	n=2	n=3	n=4	Mean	standard deviation	standard error
control	1.0	1.0	1.0		1.0	0.0	0.0
CDCA 6h	1.1	1.4	1.4		1.3	0.2	0.1
CDCA 24h	1.0	0.8	1.0		0.9	0.1	0.1
control	1.0	1.0	1.0	1.0	1.0	0.0	0.0
TCDCA 6h	1.0	0.5	0.8	0.7	0.7	0.2	0.1
TCDCA 24h	0.5	0.1	0.3	0.3	0.3	0.2	0.1
control	1.0	1.0	1.0		1.0	0.0	0.0
GCDCA 6h	2.5	0.8	1.0		1.4	0.9	0.5
GCDCA 24h	2.9	0.7	0.7		1.4	1.2	0.7

pRIPK3	n=1	n=2	n=3	n=4	Mean	standard deviation	standard error
control	1.0	1.0	1.0		1.0	0.0	0.0
CDCA 6h	1.5	1.1	1.6		1.4	0.3	0.2
CDCA 24h	1.7	0.6	1.1		1.2	0.5	0.3
control	1.0	1.0	1.0	1.0	1.0	0.0	0.0
TCDCA 6h	0.6	0.3	0.6	0.4	0.5	0.2	0.1
TCDCA 24h	0.3	0.1	0.1	0.2	0.2	0.1	0.0
control	1.0	1.0	1.0		1.0	0.0	0.0
GCDCA 6h	1.5	0.7	1.3		1.2	0.4	0.2
GCDCA 24h	1.6	0.6	1.0		1.1	0.5	0.3

FigureS7C

LCA

RIPK3	n=1	n=2	n=3	n=4	Mean	standard deviation	standard error
control	1.0	1.0	1.0		1.0	0.0	0.0
LCA 6h	0.6	0.6	0.5		0.6	0.1	0.0
LCA 24h	0.5	0.4	0.7		0.5	0.2	0.1
control	1.0	1.0	1.0		1.0	0.0	0.0
TLCA 6h	0.3	1.0	0.8		0.7	0.3	0.2
TLCA 24h	0.2	0.4	0.3		0.3	0.1	0.1
control	1.0	1.0	1.0		1.0	0.0	0.0
GLCA 6h	0.6	0.5	0.6		0.6	0.1	0.0
GLCA 24h	0.3	0.2	0.6		0.4	0.2	0.1

pRPK3	n=1	n=2	n=3	n=4	Mean	standard deviation	standard error
control	1.0	1.0	1.0		1.0	0.0	0.0
LCA 6h	0.5	0.6	1.2		0.8	0.4	0.2
LCA 24h	0.4	0.5	0.4		0.4	0.0	0.0
control	1.0	1.0	1.0		1.0	0.0	0.0
TLCA 6h	0.3	0.6	1.1		0.7	0.4	0.2
TLCA 24h	0.3	0.4	0.8		0.5	0.2	0.1
control	1.0	1.0	1.0		1.0	0.0	0.0
GLCA 6h	0.4	0.6	0.4		0.5	0.1	0.1
GLCA 24h	0.1	0.3	0.2		0.2	0.1	0.0

Figure S9						
**pMLKL**	**n=1**	**n=2**	**n=3**	**Mean**	**standard deviation**	**standard error**
**CA6**	1.3	1.4		1.4	0.1	0.0
**CA24**	2.3	4.3		3.3	1.4	1.0
**TCA6**	1.2	0.8	0.7	0.9	0.3	0.2
**TCA24**	3.2	0.7	1.7	1.9	1.3	0.7
**GCA6**	1.8	1.0		1.4	0.6	0.4
**GCA24**	2.5	1.7		2.1	0.6	0.4
**LCA6**	0.2	0.3		0.3	0.1	0.1
**LCA24**	0.2	0.5		0.4	0.2	0.2
**TLCA6**	0.6	0.5		0.6	0.1	0.1
**TLCA24**	0.2	0.3		0.3	0.1	0.1
**GLCA6**	0.7	0.9		0.8	0.1	0.1
**GLCA24**	0.6	0.4		0.5	0.1	0.1
**UDCA6**	1.5	1.7		1.6	0.1	0.1
**UDCA24**	2.4	5.2		3.8	2.0	1.4
**TUDCA6**	0.8	4.2	0.9	2.0	1.9	1.1
**TUDCA24**	1	8.6	1.4	3.7	4.3	2.5
**GUDCA6**	0.4	1.8	0.4	0.9	0.8	0.5
**GUDCA24**	1.1	5.2	1.3	2.5	2.3	1.3
**CDCA6**	0.7	0.8		0.8	0.1	0.1
**CDCA24**	0.7	0.5		0.6	0.1	0.1
**TCDCA6**	1.2	3.2		2.2	1.4	1.0
**TCDCA24**	0.9	1.3		1.1	0.3	0.2
**GCDCA6**	1	1.9	0.9	1.3	0.6	0.3
**GCDCA24**	1.1	3.9	0.7	1.9	1.7	1.0

Figure S10

	live	dead$^+$	pRIPK$^+$	pRIPK$^+$ + dead$^+$	total amount
**native**	**0.97862145**	**0.02137855**	**0**	**0**	**2713**
**CA**	0.96031128	0.03968872	0	0	2570
**RIPK3[S199A]**	0.96633086	0.03366914	0	0	3772
**RIPK3**	0.47501068	0.30414353	0.208457924	0.012387868	2341
**RIPK3+CA**	0.13222222	0.32277778	0.412222222	0.132777778	1800
**RIPK3[S199D][S227D]**	0.05562914	0.64370861	0.142384106	0.158278146	1510

condition measured	total cells	relative				%				amount			
		live	dead$^+$ + RIPK3$^-$	dead$^-$ + RIPK3$^+$	pRIPK$^+$ + dead$^+$	live	dead$^+$ + RIPK3$^-$	dead$^-$ + RIPK3$^+$	pRIPK$^+$ + dead$^+$	live	dead$^+$ + RIPK3$^-$	dead$^-$ + RIPK3$^+$	pRIPK$^+$ + dead$^+$
**native**	2713	0.97862145	0.021378548	0	0	98	2	0	0	2655	58	0	0
**CA**	2570	0.96031128	0.039688716	0	0	96	4	0	0	2468	102	0	0
**RIPK3[S199A]**	3772	0.96633086	0.033669141	0	0	97	3	0	0	3645	127	0	0
**RIPK3**	2341	0.47501068	0.304143528	0.208457924	0.012387868	48	30	21	1	1112	712	488	29
**RIPK3+CA**	1800	0.13222222	0.322777778	0.412222222	0.132777778	13	32	41	13	238	581	742	239
**RIPK3[S199D][S227D]**	1510	0.05562914	0.643708609	0.142384106	0.158278146	6	64	14	16	84	972	215	239
**CONTROL* (summe CA+nativ + RIPK[Ser199Ala])**	9055	9130.37277	298.8614263	0	0	913037	29886	0	0	8768	287	0	0

Figure S11

Ct raw data

IL-33			HMBG1			IL-6			HPRT		
control	ack	ack_CA	control	ack	ack_CA	control	ack	ack_CA	control	ack	ack_CA
29.2	29.4	30.8	24.1	24.1	29.3	28.4	31.0	32.5	18.0	18.5	18.6
29.7	28.8	30.9	24.1	23.7	24.3	29.7	29.4	31.7	18.1	18.4	20.6
31.2	29.8		24.3	27.0	29.5		32.5		17.8	18.8	18.8
29.3	30.9		24.1	24.1	25.7		32.5		17.8	17.9	18.5
29.6	30.0	29.3	24.4	24.5	24.4			32.6	18.3	18.5	18.5
29.7	29.8		24.2	24.7	24.6			32.5	18.2	18.4	
	30.8										

2^(CT(mean control) - CT(sample))

IL-33			HMBG1			IL-6			HPRT		
control	ack	ack_CA	control	ack	ack_CA	control	ack	ack_CA	control	ack	ack_CA
1.5	1.3	0.5	1.1	1.1	0.0	1.6	0.3	0.1	1.0	0.7	0.6
1.0	2.0	0.5	1.1	1.4	0.9	0.6	0.8	0.2	0.9	0.8	0.2
0.4	1.0		0.9	0.1	0.0		0.1		1.2	0.6	0.6
1.4	0.5		1.0	1.1	0.4		0.1		1.2	1.1	0.7
1.2	0.8	1.4	0.9	0.8	0.9			0.1	0.8	0.7	0.7
1.1	1.0		1.0	0.7	0.7			0.1	0.9	0.8	
	0.5										

sample/ HPRT

IL-33			HMBG1			IL-6		
control	ack	ack_CA	control	ack	ack_CA	control	ack	ack_CA
1.4	1.9	0.7	1.1	1.6	0.0	1.6	0.4	0.1
1.1	2.6	2.8	1.2	1.8	5.5	0.7	1.0	1.0
0.3	1.7		0.8	0.3	0.0		0.2	
1.2	0.4		0.9	1.0	0.5		0.1	
1.4	1.2	2.0	1.0	1.1	1.2			0.1
1.2	1.3		1.2	1.0				
	0.5							

Log2

	IL-33			HMBG1			IL-6		
	0.5	0.9	-0.4	0.1	0.7	-4.4	0.6	-1.4	-2.8
	0.1	1.4	1.5	0.2	0.9	2.5	-0.6	0.0	-0.1
	-1.7	0.8		-0.4	-2.0	-4.5		-2.7	
	0.3	-1.2		-0.2	0.0	-1.0		-3.5	
	0.4	0.2	1.0	0.0	0.2	0.3			-3.1
	0.3	0.4		0.2	-0.1				
		-1.1							
**mean**	**0.0**	**0.2**	**0.7**	**0.0**	**-0.1**	**-1.4**	**0.0**	**-1.9**	**-2.0**
**standard deviation**	**0.8**	**1.0**	**1.0**	**0.2**	**1.0**	**3.0**	**0.9**	**1.5**	**1.7**
**standard error**	**0.3**	**0.4**	**0.6**	**0.1**	**0.4**	**1.4**	**0.6**	**0.8**	**1.0**
**amount**	**6.0**	**7.0**	**3.0**	**6.0**	**6.0**	**5.0**	**2.0**	**4.0**	**3.0**

Figure S12						
**control**	**Anisomycine**			**RIPK3 + Anisomycine**		
	**15 min**	**30 min**	**24 h**	**15 min**	**30 min**	**24 h**
0.0	3735.8	13891.8	401.8	3290.2	13392.1	3434.0
0.0	5888.6	7903.4	15.9	2215.0	11214.7	0.0

Figure S13

	control	STS	RIPK3	RIPK3[S199A]	RIPK3[S227A]	RIPK3[S199/227A]	RIPK3[S199/227D]
	1.197844008	1.56139891	1.996816423	2.125617028	1.370227606	1.812811122	1.276702666
	1.006617263	1.63392947	1.267852306	1.744598942	1.571319029		1.648593911
	0.836466527		2.705187007	1.492024015	1.894389517	2.529650965	1.188880976
	0.959072203	2.41144576	1.975855754	2.458482524	1.768863842	1.856757698	2.075567873
	0.974406765	2.18755635	2.006328602	2.060107418	1.652837049	1.947637929	1.543201883
	1.302153067	1.96423585	1.512575616	1.656319084			1.107264808
	1.189831671	3.110787	1.7400678	1.598598873			2.487539368
	0.819439265		1.516780821	1.338664097			2.177039255
	0.714169232		1.400015162	2.485626297			1.899905467
**mean**	1	2.14489222	1.791275499	1.884448698	1.651527409	2.036714428	1.711632912
**standard deviation**	0.196816311	0.57271421	0.438819253	0.41610345	0.198943787	0.333385746	0.480438178
**amount**	9	6	9	9	5	4	9
**standard error**	0.065605437	0.2338096	0.146273084	0.13870115	0.088970366	0.166692873	0.160146059

* values relative to mean control

Figure S14

	control	CA
n=1	1	4.12616828
n=2	1	1.65544861
n=3	1	2.36608861

Table S3

animal	ASAT (µmol/ L*s)																					
1		4,61				5.22		12.7						36.64				7.96		5.33		6.39
2						5.12								8.12				2.96		7.54		4.08
3		3.59		3.72		8.28								10.24		3.31		4.32		3.76		4.45
4	sham OP 7d	2.51	sham OP 3d	6.21	sham OP 1d	5.8	BDL 7d	13.67	BDL 3d	19.85	BDL 1d		IR	42.40	APAP		PBS	3.85	Ringer	3.21	PCI	4.1
5				4.01		5.66				27.09										2.93		3.2
6		3.03				3.27								7.43		23.96				4.21		3.27
7																				3.75		3.91
8																				4.67		5.16
	**mean**	**3.0**	**mean**	**4.6**	**mean**	**5.6**	**mean**	**13.2**	**mean**	**23.5**	**mean**		**mean**	**21.0**	**mean**	**13.6**	**mean**	**4.8**	**mean**	**4.4**	**mean**	**4.3**
	**SD**	**0.5**	**SD**	**1.4**	**SD**	**1.6**	**SD**	**0.7**	**SD**	**5.1**	**SD**		**SD**	**17.1**	**SD**	**14.6**	**SD**	**2.2**	**SD**	**1.5**	**SD**	**1.0**
	**SE**	**0.3**	**SE**	**0.8**	**SE**	**0.7**	**SE**	**0.3**	**SE**	**3.0**	**SE**		**SE**	**8.5**	**SE**	**8.4**	**SE**	**0.9**	**SE**	**0.5**	**SE**	**0.4**

animal	ALAT (µmol/ L*s)																					
1		0.67				0.67		10.46										1.08		0.62		0.8
2						1.18								4.60				0.97		0.83		0.74
3		0.58		0.47		0.78						71.07		11.11		1.66		0.86				0.62
4	sham OP 7d	0.43	sham OP 3d		sham OP 1d	0.79	BDL 7d	8.76	BDL 3d	8.62	BDL 1d		IR		APAP		PBS	0.82	Ringer	1.14	PCI	
5						2.06		12.51		11.3						1.99				0.9		1.18
6		0.53				0.65		10.04						7.49		61.30				0.67		0.79
7																				0.97		0.62
8																				0.56		1.21
	**mean**	**0.6**	**mean**		**mean**	**1.0**	**mean**	**10.4**	**mean**	**10.0**	**mean**		**mean**	**7.7**	**mean**	**21.7**	**mean**	**0.9**	**mean**	**0.8**	**mean**	**0.9**
	**SD**	**0.1**	**SD**		**SD**	**0.5**	**SD**	**1.6**	**SD**	**1.9**	**SD**		**SD**	**3.3**	**SD**	**34.3**	**SD**	**0.1**	**SD**	**0.2**	**SD**	**0.2**
	**SE**	**0.1**	**SE**		**SE**	**0.2**	**SE**	**0.8**	**SE**	**1.1**	**SE**		**SE**	**1.6**	**SE**	**19.8**	**SE**	**0.0**	**SE**	**0.1**	**SE**	**0.1**

animal	body weight (g)																					
1		-0.2		-0.7		-0.8		-0.2		-3.7		-1		-1.60		1.00		2.80		0.6		-2.2
2		0.1		-1.2		0.4		-1.1		-3		-1.8		-0.50		-1.40		2.70		0.4		-2.4
3		-0.7		-2.8		-0.3		-3.9		-3.3		-1		-3.00		-1.00		2.30		0.6		-1.5
4	sham OP 7d	-1	sham OP 3d	-1.4	sham OP 1d	-1.6	BDL 7d	-0.4	BDL 3d	-0.6	BDL 1d	-2.4	IR	-0.80	APAP	-0.60	PBS	1.90	Ringer	0.7	PCI	-3.4
5		-2		0.5		-0.3		-2.7		-0.3		-1.3		-1.50		0.70				1		-1.8
6		-1.1		-0.4		0.1		-1.1		-1.9		-0.7		-1.60		-1.00				0.7		-2.8
7																				0.9		-3.3
8																				-0.6		-2.1
	**mean**	**-0.8**	**mean**	**-1.0**	**mean**	**-0.4**	**mean**	**-1.6**	**mean**	**-2.1**	**mean**	**-1.4**	**mean**	**-1.5**	**mean**	**-0.4**	**mean**	**2.4**	**mean**	**0.5**	**mean**	**-2.4**
	**SD**	**0.7**	**SD**	**1.1**	**SD**	**0.7**	**SD**	**1.4**	**SD**	**1.4**	**SD**	**0.6**	**SD**	**0.9**	**SD**	**1.0**	**SD**	**0.4**	**SD**	**0.5**	**SD**	**0.7**
	**SE**	**0.4**	**SE**	**0.6**	**SE**	**0.3**	**SE**	**0.7**	**SE**	**0.8**	**SE**	**0.3**	**SE**	**0.4**	**SE**	**0.6**	**SE**	**0.2**	**SE**	**0.2**	**SE**	**0.2**

Table S4

Sample	Group	age (years)	gender	Bili (< 21)	ASAT	ALAT	Albumin	CRP	diagnosis
**1**	**reference**	63	m	11.4	0.41	0.3	40.8	5	metastatic sigma carcinoma
**2**		80	w	10	0.37	0.26	32	3.8	kaltskin carcinoma
**3**		70	w	8	0.23	0.3	35	3	metastatic sigma carcinoma
**5**		26	m	16	0.37	0.38	41	3	metastatic insulinoma
**6**		66	w	6	0.33	0.51	35	3	metastatic colon carcinoma
**7**		70	m	8	0.37	0.54	44	2.8	metastatic rectal carcinoma
**8**		45	w	11	0.42	0.37	34	66.5	adenoma
**9**		54	w	20	0.77	1.87	32	56.2	breast carcinoma
**10**		54	m	6	0.72	0.33	35	29.3	metastatic sigma carcinoma
**11**		43	w	21	0.2	0.28	29	83.2	adenoma
**12**		63	m	9	1.08	0.44	34	133.9	metastatic rectal carcinoma
**13**		29	w	11	0.56	0.62	24	91.5	liver hematoma
**14**		74	m	10	0.63	0.43	26	91.9	hepatocellular carcinoma
**24**		58	w					6.9	adenocarcinoma rectal
**25**		45	m	17	0.32	0.62	45	5.7	adenocarcinoma ovary
**31**		75	w	12	0.44	0.43	35	3.2	adenocarcinoma pancreas
**38**		52	w	14	0.34	0.71	39	9.2	adenocarcinoma colon
**40**		61	w	11	0.33	0.49	22	336.4	adenocarcinoma stomach
**41**		51	w	9.7	0.44	0.55	37.6	104	squamous cell carcinoma
**51**		67	m	9	0.41	0.67	41	10.7	non-alcoholic steatohepatitis

**Mean**	**57**		**11.6**	**0.5**	**0.5**	**34.8**	**52.5**
**SD**	**14.6356191**		**4.3**	**0.2**	**0.4**	**6.4**	**79.3**
**alpha**	**0.05**						
**amount**	**20**						
**KI**	**6.41422501**						
**male**		**8**					
**femal**		**12**					

Sample	Group	age (years)	gender	Bili (< 21)	ASAT	ALAT	Albumin	CRP	diagnosis
**15**	**cholestasis**	76	m	**24**	0.66	0.43	28	8.5	metastatic colon carcinoma
**17**		48	m	**21**	0.41	0.26	37	3	metastatic cecum carcinoma
**18**		44	w	**148**	1.18	1.52	30	13.9	gall bladder carcinoma
**19**		70	m	**125.5**	0.32	0.82	29		klatskin carcinoma
**42**		66	m	**37**	0.35	0.47	31	3.9	adenocarcinoma pancreas
**50**		71	m	**119**	0.93	0.36	29	7.7	liver cirrhosis
**56**		66	w	**34**	0.95	0.46	34	16.9	liver cirrhosis
**57**		63	w	**22**	0.82	0.52	26	7.2	liver cirrhosis
**49**		29	w	**139**	8.37	0.93	25	56.3	non-alcoholic steatohepatitis

**Mean**	**59**		**74.4**	**1.6**	**0.6**	**29.9**	**14.7**
**SD**	**15.4746536**		**56.3**	**2.6**	**0.4**	**3.8**	**17.5**
**alpha**	**0.05**						
**amount**	**9**						
**KI**	**10.1099212**						
**male**		**5**					
**femal**		**4**					

Table S6

pMak

RIPK1

		Blot	Gel	Ratio Blot	Ratio Gel	mean
n=1	- TBZ	11686.38	67729.67	1.0	1.0	**1.0**
	+ TBZ	20594.936	71650.841	1.8	0.9	**1.7**
n=2	- TBZ	11662.5	97550.2	1.0	1.0	**1.0**
	+ TBZ	24342.5	91447.1	2.1	1.1	**2.2**

pRIPK1

		Blot	Gel	Ratio Blot	Ratio Gel	
n=1	- TBZ	16805.4	34189.4	1.0	1.0	**1.0**
	+ TBZ	27384.5	65137.1	1.6	0.5	**0.9**
n=2	- TBZ	1908.7	68626.0	1.0	1.0	**1.0**
	+ TBZ	13785.7	92954.4	7.2	0.7	**5.3**

RIPK3

		Blot	Gel	Ratio Blot	Ratio Gel	
n=1	- TBZ	25855.0	75522.1	1.0	1.0	**1.0**
	+ TBZ	14721.6	81716.4	0.6	0.9	**0.5**
n=2	- TBZ	26256.6	72159.8	1.0	1.0	**1.0**
	+ TBZ	26726.6	86562.1	1.0	0.8	**0.8**
n=3	- TBZ	16347.6	58443.4	1.0	1.0	**1.0**
	+ TBZ	20733.6	62578.1	1.3	0.9	**1.2**
n=4	- TBZ	15438.7	124521.8	1.0	1.0	**1.0**
	+ TBZ	15073.8	130776.2	1.0	1.0	**0.9**

pRIPK3

		Blot	Gel	Ratio Blot	Ratio Gel	
n=1	- TBZ	13834.6	31490.7	1.0	1.0	**1.0**
	+ TBZ	12262.4	44747.7	0.9	0.7	**0.6**
n=2	- TBZ	20772.4	89231.4	1.0	1.0	**1.0**
	+ TBZ	21837.6	79581.4	1.1	1.1	**1.2**
n=3	- TBZ	17490.9	77364.8	1.0	1.0	**1.0**
	+ TBZ	16871.7	105723.4	1.0	0.7	**0.7**

MLKL

		Blot	Gel	Ratio Blot	Ratio Gel	
n=1	- TBZ	22768.5	72764.5	1.0	1.0	**1.0**
	+ TBZ	24918.5	82441.2	1.1	0.9	**1.0**
n=2	- TBZ	18391.0	56355.3	1.0	1.0	**1.0**
	+ TBZ	17032.0	69864.2	0.9	0.8	**0.7**

pMLKL

		Blot	Gel	Ratio Blot	Ratio Gel	
n=1	- TBZ	6536.0	11941.1	1.0	1.0	**1.0**
	+ TBZ	11291.7	44754.6	1.7	0.3	**0.5**
n=2	- TBZ	10598.8	69299.0	1.0	1.0	**1.0**
	+ TBZ	10729.1	102647.0	1.0	0.7	**0.7**

pHep

RIPK1

		Blot	Gel	Ratio Blot	Ratio Gel	mean
n=1	- TBZ	380021.0	94692963.0	1.0	1.0	**1.0**
	+ TBZ	21855522.0	59792963.0	57.5	1.6	**91.1**
n=2	- TBZ	1740.6	100418.5	1.0	1.0	**1.0**
	+ TBZ	18449.0	73731.5	10.6	1.4	**14.4**

pRIPK1

		Blot	Gel	Ratio Blot	Ratio Gel	
n=1	- TBZ	1151.3	115489.5	1.0	1.0	**1.0**
	+ TBZ	25787.8	99775.7	22.4	1.2	**25.9**
n=2	- TBZ	11656.4	51919.1	1.0	1.0	**1.0**
	+ TBZ	19171.8	52607.7	1.6	1.0	**1.6**

RIPK3

		Blot	Gel	Ratio Blot	Ratio Gel	
n=1	- TBZ	nd	95494.5	nd	1.0	**nd**
	+ TBZ	nd	51288.0	nd	1.9	**nd**
n=2	- TBZ	nd	133164.1	nd	1.0	**nd**
	+ TBZ	nd	77084.5	nd	1.7	**nd**
n=3	- TBZ	nd	62025.3	nd	1.0	**nd**
	+ TBZ	nd	69969.4	nd	0.9	**nd**
n=4	- TBZ	nd	145672.1	nd	1.0	**nd**
	+ TBZ	nd	137854.3	nd	1.1	**nd**

pRIPK3

		Blot	Gel	Ratio Blot	Ratio Gel	
n=1	- TBZ	nd	76411.7	nd	1.0	**nd**
	+ TBZ	nd	57260.7	nd	1.3	**nd**
n=2	- TBZ	nd	85721.1	nd	1.0	**nd**
	+ TBZ	nd	119962.5	nd	0.7	**nd**
n=3	- TBZ	nd	89445.1	nd	1.0	**nd**
	+ TBZ	nd	94501.4	nd	0.9	**nd**

MLKL

		Blot	Gel	Ratio Blot	Ratio Gel	
n=1	- TBZ	15696.0	95304.6	1.0	1.0	**1.0**
	+ TBZ	13653.5	100932.9	0.9	0.9	**0.8**
n=2	- TBZ	37948.9	58589.1	1.0	1.0	**1.0**
	+ TBZ	28944.5	77176.6	0.8	0.8	**0.6**

pMLKL

		Blot	Gel	Ratio Blot	Ratio Gel	
n=1	- TBZ	2439.8	66479.4	1.0	1.0	**1.0**
	+ TBZ	10786.8	93608.8	4.4	0.7	**3.1**
n=2	- TBZ	4155.0	118942.7	1.0	1.0	**1.0**
	+ TBZ	17339.2	146963.1	4.2	0.8	**3.4**

HepG2

RIPK1

		Blot	Gel	Ratio Blot	Ratio Gel	mean
n=1	- TBZ	21871149.0	79922335.0	1.0	1.0	**1.0**
n=2	- TBZ	19936451.0	78365.6	0.9	1019.9	**929.6**
n=3	- TBZ	22844.5	125836.8	0.0	635.1	**0.7**

pRIPK1

		Blot	Gel	Ratio Blot	Ratio Gel	
n=1	- TBZ	nd	16975.5			**nd**
n=2	- TBZ	nd				**nd**
n=3	- TBZ	nd				**nd**

RIPK3

		Blot	Gel	Ratio Blot	Ratio Gel	
n=1	- TBZ	nd				**nd**
n=2	- TBZ	nd				**nd**
n=3	- TBZ	nd				**nd**
n=4	- TBZ	nd				**nd**
n=5	- TBZ	nd				**nd**

pRIPK3

		Blot	Gel	Ratio Blot	Ratio Gel	
n=1	- TBZ	nd				**nd**
n=2	- TBZ	nd				**nd**
n=3	- TBZ	nd				**nd**

MLKL

		Blot	Gel	Ratio Blot	Ratio Gel	
n=1	- TBZ	15434.6	139328.0	1.0	1.0	**1.0**
n=2	- TBZ	15167.1	78671.0	1.0	1.8	**1.7**

pMLKL

		Blot	Gel	Ratio Blot	Ratio Gel	
n=1	- TBZ	nd	24946.9			**nd**
n=2	- TBZ	nd	118263.8			**nd**

Table S7					
chrom	position	strand	female_meth_ratio	male_meth_ratio	NPC_meth_ratio
**chr14**	**24809110**	**-**	0.666999556	0.763636364	0.191177554
**chr14**	**24809162**	**-**	0.268729282	0.336589404	0.067450404
**chr14**	**24809178**	**-**	0.401086113	0.541909197	0.118049426
**chr14**	**24809197**	**-**	0.387676367	0.460287318	0.111102963
**chr14**	**24809226**	**-**	0.512617488	0.60839011	0.113210388
**chr14**	**24809240**	**-**	0.518875235	0.581474417	0.148265683
**chr14**	**24809268**	**-**	0.740912606	0.840751158	0.148893952
**chr14**	**24809297**	**-**	0.608152894	0.745845391	0.144882815
**chr14**	**24809315**	**-**	0.682886278	0.766359791	0.149342008
		mean	**0.531992869**	**0.627249239**	**0.132486132**
		%	**53**	**63**	**13**
