# Supplementary material for: RIPK3 promoter hypermethylation in hepatocytes protects from bile acid-induced inflammation and necroptosis
Source: Cell Death Dis. 2023 Apr 18;14(4):275. doi: 10.1038/s41419-023-05794-0 (PMC10113265; doi:10.1038/s41419-023-05794-0)
Supplement: Supplementary file 3 — Supplementary Material - 3 [file 41419_2023_5794_MOESM3_ESM.pdf]

Supplementary Material - 3

RIPK3 promoter hypermethylation in hepatocytes protects from bile acid-induced inflammation and necroptosis

Jessica Hoff[1,2], Ling Xiong[1,2], Tobias Kammann[1,2], Sophie Neugebauer[3], Julia M. Micheel[1,2], Nikolaus Gaßler[4], Michael Bauer[1,2], Adrian T. Press[1,2,5]

[1] Department of Anesthesiology and Intensive Care Medicine, Nanophysiology Group, Jena University Hospital, Jena 07747, Germany
[2] Center for Sepsis Control and Care, Jena University Hospital, Jena 07743, Germany
[3] Department of Clinical Chemistry and Laboratory Diagnostics, Jena University Hospital, Jena 07747, Germany
[4] Pathology, Jena University Hospital, Jena 07747, Germany
[5] Faculty of Medicine, Friedrich Schiller University Jena, Jena 07747, Germany

Correspondence
Adrian Press,
Am Klinikum 1, 07747 Jena
+49 3641/ 9 323139
Adrian.Press@med.uni-jena.de

Keywords
necroptosis, hepatocytes, RIPK3, bile acids, methylation, inflammation

Supplementary Information – Significances

Figure 1A/ 1B

Figure 1A and 1B show western blot pictures without statistical analysis.

Figure 1C

Statistical analysis are shown in figure S1B. Table with n-number are depicted in Table S5.

Figure 1D

Figure 1D shows methylation analysis without statistical analysis.

Figure 2A

Figure 2A show western blot images. Quantification in Figure 2B.

Figure 2B

expression

test: z-test
groups: control vs. treatment
parameters: α = 0.05

results:

	expression	
PBS / APAP	p-value:	p = 2.000
	Significance:	no
Ringer / PCI	p-value:	p < 0.001
	Significance:	yes
surgery 1d / BDL 1d	p-value:	p = 0.003
	Significance:	yes
surgery 3d / BDL 3d	p-value:	p = 0.014
	Significance:	yes
surgery 7d / BDL 7d	p-value:	p = 0.022
	Significance:	yes
non-ischemic / ischemic	p-value:	p = 0.121
	Significance:	no

intensity

test: t-test
groups: control vs. treatment
parameters: α = 0.05
Normality: Shapiro-Wilk
Equal varian Brown-Forsythe

results:

PBS	APAP	surgery 3d	BDL 3d
Normality:	failed (p < 0.05)	Normality:	failed (p < 0.05)
Equal variance:	not tested	Equal variance:	not tested
Mann- Whitney Test:	p = 1.000	Mann- Whitney Test:	p = 0.002
Significance:	no	Significance:	yes

Ringer	PCI	surgery 7d	BDL 7d
Normality:	passed (p = 0.122)	Normality:	failed (p < 0.05)
Equal variance:	passed (p = 1.000)	Equal variance:	not tested
Student's t-test:	p = 0.406	Mann- Whitney Test:	p = 0.02
Significance:	yes	Significance:	yes

surgery 1d	BDL 1d	non-ischemic	ischemic
Normality:	passed (p = 0.128)	Normality:	failed (p < 0.05)
Equal variance:	passed (p < 0.05)	Equal variance:	not tested
Welch's t-test:	p = 0.019	Mann- Whitney Test:	p = 0.818
Significance:	yes	Significance:	no

Figure 2C

Figure 2C shows a heatmap without statistical analysis.

Figure 2D

test: One-way ANOVA
groups: vs. PBS
parameters: α = 0.05
Normality: Shapiro-Wilk
Equal varian Brown-Forsythe

results: Normality: failed (p < 0.05)
Equal variance: not tested
Kruskal-Wallis with Dunn's test

APAP	p-value:	not tested
	Significance:	no
Ringer	p-value:	not tested
	Significance:	no
PCI	p-value:	0.175
	Significance:	no
surgery 1d	p-value:	0.042
	Significance:	yes
BDL 1d	p-value:	< 0.001
	Significance:	yes
surgery 3d	p-value:	not tested
	Significance:	no
BDL 3d	p-value:	0.001
	Significance:	yes
surgery 7d	p-value:	not tested
	Significance:	no
BDL 7d	p-value:	0.009
	Significance:	yes
non-ischemic	p-value:	not tested
	Significance:	no
ischemic	p-value:	not tested
	Significance:	no

Figure 2E

Figure 2E show representative fluorescent stainings. (Analysis in Figure 2 F)

Figure 2F

test: t-Test
groups: reference vs. cholestasis
parameters: α = 0.05
Normality: Shapiro-Wilk
Equal varian Brown-Forsythe

results:

LSEC	
reference	cholestasis
Normality:	passed (p = 0.398)
Equal variance:	passed (p = 0.963)
Student's t-test:	p = 0.121
Significance:	no

hepatocytes	
reference	cholestasis
Normality:	passed (p = 0.747)
Equal variance:	failed (p < 0.05)
Welch's t-test:	p = 0.02
Significance:	yes

Figure 3A

Figure 3A shows western blot images without statistical analysis.

Figure 3B

Figure 3B shows a heatmap without statistical analysis.

Figure 3C

test: One-way ANOVA
groups: vs. control
parameters: α = 0.05
Normality: Shapiro-Wilk
Equal variance: Brown-Forsythe

results:

RIPK3								
**control**	**CA 6h**	**CA 24h**	**control**	**TCA 6h**	**TCA 24h**	**control**	**GCA 6h**	**GCA 24h**
Normality:	passed	p = 0.307	Normality:	passed	p = 0.162	Normality:	passed	p = 0.236
Equal variance:	passed	p = 0.571	Equal variance:	passed	p = 0.423	Equal variance:	passed	p = 1.000
One-Way ANOVA			One-Way ANOVA			One-Way ANOVA		
p-value:	0.174		p-value:	0.118		p-value:	0.154	
Significance:	no		Significance:	no		Significance:	no	

pRIPK3								
**control**	**CA 6h**	**CA 24h**	**control**	**TCA 6h**	**TCA 24h**	**control**	**GCA 6h**	**GCA 24h**
Normality:	passed	p = 0.361	Normality:	passed	p = 0.071	Normality:	passed	p = 0.129
Equal variance:	passed	p = 0.095	Equal variance:	passed	p = 1.000	Equal variance:	passed	p = 0.369
One-Way ANOVA with Dunnett's test			One-Way ANOVA with Dunnett's test			One-Way ANOVA with Dunnett's test		
p-value:	0.167	<0.001	p-value:	0.811	0.002	p-value:	0.985	0.024
Significance:	no	yes	Significance:	no	yes	Significance:	no	yes

Figure 3D

test: One-way ANOVA
groups: vs. control
parameters: α = 0.05
Normality: Shapiro-Wilk
Equal variance: Brown-Forsythe

results:

RIPK3								
**control**	**UDCA 6h**	**UDCA 24 h**	**control**	**TUDCA 6h**	**TUDCA 24h**	**control**	**GUDCA 6h**	**GUDCA 24h**
Normality:	passed	p = 0.171	Normality:	passed	p = 0.643	Normality:	passed	p = 0.482
Equal variance:	passed	p = 1.000	Equal variance:	failed	p < 0.050	Equal variance:	failed	p < 0.050
One-Way ANOVA			Kruskal-Wallis			Kruskal-Wallis		
p-value:	0.698		p-value:	0.706		p-value:	0.959	
Significance:	no		Significance:	no		Significance:	no	

pRIPK3								
**control**	**UDCA 6h**	**UDCA 24 h**	**control**	**TUDCA 6h**	**TUDCA 24h**	**control**	**GUDCA 6h**	**GUDCA 24h**
Normality:	passed	p = 0.054	Normality:	failed	p < 0.050	Normality:	failed	p < 0.050
Equal variance:	passed	p = 0.077	Equal variance:	not tested		Equal variance:	not tested	
One-Way ANOVA with Dunnett's test			Kruskal-Wallis			Kruskal-Wallis		
p-value:	0.998	0.031	p-value:	0.452		p-value:	0.307	
Significance:	no	yes	Significance:	no		Significance:	no	

Figure 3E

Figure 3E shows western blot images without statistical analysis.

Figure 3F

test: z-test with Bonferroni adjusted α-value
groups: indicated below
parameters: α = 0.05/11 conditions = 0.00455

results:

	**p-value**	
**native vs. CA = control**	0.094	**ns**
**control vs. RIPK3[Ser199Ala]**	<0.001	yes
**control vs. RIPK3[Ser199Asp][Ser227Asp]**	<0.001	yes
**control vs. RIPK3**	<0.001	yes
**control vs. RIPK3 + CA**	<0.001	yes
**RIPK3 + CA vs. RIPK3**	<0.001	yes
**RIPK3 + CA vs. RIPK3[Ser199Ala]**	<0.001	yes
**RIPK3 + CA vs. RIPK3[Ser199Asp][Ser227Asp]**	<0.001	yes
**RIPK3 vs. RIPK3[Ser199Ala]**	<0.001	yes
**RIPK3 vs. RIPK3[Ser199Asp][Ser227Asp]**	<0.001	yes
**RIPK3[Ser199Asp][Ser227Asp] vs. RIPK3[Ser199Ala]**	<0.001	yes

Figure 4A

**test:**	One-way ANOVA			
**groups:**	vs. control			
**parameters:**	α = 0.05			
**Normality:**	Shapiro-Wilk			
**Equal variance:**	Brown-Forsythe			
**results:**				
Normality:	failed	$p < 0.050$		
Equal variance:	not tested			
Kruskal-Wallis with Dunn's test				
	**control**	**RIPK3**	**RIPK3 + CA**	
p-value:		<0.001	<0.001	
Significance:		yes	yes	

Figure 4B

**test:**	One-way ANOVA			
**groups:**	vs. RIPK3			
**parameters:**	α = 0.05			
**Normality:**	Shapiro-Wilk			
**Equal variance:**	Brown-Forsythe			
**results:**				
Normality:	passed	$p = 0.685$		
Equal variance:	failed	$p < 0.050$		
Kruskal-Wallis with Dunn's test				
	**RIPK3**	**[S199D]**	**[S227D]**	**[S199/227D]**
p-value:		0.278	0.258	<0.001
Significance:		no	no	yes

Figure 4C

**test:**	t-Test
**groups:**	HepG2 vs. RIPK3
**parameters:**	α = 0.05
**Normality:**	Shapiro-Wilk
**Equal variance:**	Brown-Forsythe

results:

Normality:	failed	$p < 0.050$
Equal variance:	not tested	
Mann-Whitney Test		

	HepG2 vs. RIPK3
p-value:	<0.001
Significance:	yes

**test:**	Two-way ANOVA
**groups:**	all pairwise
**parameters:**	α = 0.05
**Normality:**	Shapiro-Wilk
**Equal variance:**	Brown-Forsythe

results:

CA		
Normality:	passed	p = 0.143
Equal variance:	passed	p = 0.057
One-Way ANOVA with Tukey test		
	p-value:	Significance:
CA - RIPK3 vs. CA + RIPK3	< 0.001	yes
CA - RIPK3 vs. GCA + RIPK3	0.270	no
CA - RIPK3 vs. TCA + RIPK3	0.029	yes
CA + RIPK3 vs. GCA + RIPK3	0.270	no
CA + RIPK3 vs. TCA + RIPK3	0.004	yes
GCA + RIPK3 vs. TCA + RIPK3	0.575	no

CDCA		
Normality:	passed	p = 0.180
Equal variance:	passed	p = 0.242
One-Way ANOVA with Tukey test		
	p-value:	Significance:
CDCA - RIPK3 vs. CDCA + RIPK3	< 0.001	yes
CDCA - RIPK3 vs. GCDCA + RIPK3	0.02	yes
CDCA - RIPK3 vs. TCDCA + RIPK3	0.002	yes
CDCA + RIPK3 vs. GCDCA + RIPK3	0.002	yes
CDCA + RIPK3 vs. TCDCA + RIPK3	0.006	yes
GCDCA + RIPK3 vs. TCDCA + RIPK3	0.875	no

UDCA		
Normality:	passed	p = 0.391
Equal variance:	passed	p = 0.596
One-Way ANOVA with Tukey test		
	p-value:	Significance:
UDCA - RIPK3 vs. UDCA + RIPK3	< 0.001	yes
UDCA - RIPK3 vs. GUDCA + RIPK3	< 0.001	yes
UDCA - RIPK3 vs. TUDCA + RIPK3	< 0.001	yes
UDCA + RIPK3 vs. GUDCA + RIPK3	< 0.001	yes
UDCA + RIPK3 vs. TUDCA + RIPK3	< 0.001	yes
GUDCA + RIPK3 vs. TUDCA + RIPK3	0.114	no

LCA		
Normality:	passed	p = 0.072
Equal variance:	passed	p = 0.396
One-Way ANOVA with Tukey test		
	p-value:	Significance:
LCA - RIPK3 vs. LCA + RIPK3	0.007	yes
LCA - RIPK3 vs. GLCA + RIPK3	0.393	no
LCA - RIPK3 vs. TLCA + RIPK3	0.444	no
LCA + RIPK3 vs. GLCA + RIPK3	0.1	no
LCA + RIPK3 vs. TLCA + RIPK3	0.081	no
GLCA + RIPK3 vs. TLCA + RIPK3	1	no

Figure 4D

**test:**	One-way ANOVA
**groups:**	vs. control
**parameters:**	α = 0.05
**Normality:**	Shapiro-Wilk
**Equal variance:**	Brown-Forsythe

results:

Normality:	passed	p = 0.262
Equal variance:	failed	$p < 0.050$
Kruskal-Wallis with Dunn's test		

	RIPK3-pcDNA	CA	CDCA	UDCA	LCA
p-value:	0.156	0.018	0.241	0.003	0.262
Significance:	no	yes	no	yes	no

Figure S1A

Figure S1A shows western blot pictures without statistical analysis.

Figure S1B

test: t-Test
groups: -TBZ vs. +TBZ
parameters: α = 0.05
Normality: Shapiro-Wilk
Equal variance: Brown-Forsythe

results:

hepatocytes		
**RIPK1**	Normality:	passed ($p = 0.683$)
	Equal variance:	failed ($p < 0.05$)
	Welch's t-test:	$p = 0.406$
	Significance:	no
**pRIPK1**	Normality:	passed ($p = 0.683$)
	Equal variance:	failed ($p < 0.05$)
	Welch's t-test:	$p = 0.484$
	Significance:	no
**MLKL**	Normality:	passed ($p = 0.683$)
	Equal variance:	failed ($p < 0.05$)
	Welch's t-test:	$p = 0.245$
	Significance:	no
**pMLKL**	Normality:	passed ($p = 0.683$)
	Equal variance:	failed ($p < 0.05$)
	Welch's t-test:	$p = 0.033$
	Significance:	yes

macrophages		
**RIPK1**	Normality:	passed ($p = 0.683$)
	Equal variance:	failed ($p < 0.05$)
	Welch's t-test:	$p = 0.183$
	Significance:	no
**pRIPK1**	Normality:	passed ($p = 0.683$)
	Equal variance:	failed ($p < 0.05$)
	Welch's t-test:	$p = 0.521$
	Significance:	no
**RIPK3**	Normality:	failed ($p < 0.05$)
	Equal variance:	not tested
	Mann-Whitney Test:	$p = 0.343$
	Significance:	no
**pRIPK3**	Normality:	passed ($p = 0.198$)
	Equal variance:	passed ($p = 1.000$)
	Student's t-test:	$p = 0.397$
	Significance:	no
**MLKL**	Normality:	passed ($p = 0.683$)
	Equal variance:	failed ($p < 0.05$)
	Welch's t-test:	$p = 0.415$
	Significance:	no
**pMLKL**	Normality:	passed ($p = 0.683$)
	Equal variance:	failed ($p < 0.05$)
	Welch's t-test:	$p = 0.162$
	Significance:	no

Figure S2

Figure S2 shows western blot pictures without statistical analysis.

Figure S3

Figure S3 shows western blot pictures without statistical analysis.

Figure S4

Figure S4 shows western blot pictures without statistical analysis.

Figure S5

test: One-way ANOVA
groups: vs. 0.000064
parameters: α = 0.05
Normality: Shapiro-Wilk
Equal variance: Brown-Forsythe

results:

LCA 6h

50	25	10	5	2	0.4	0.16	0.08	0.016	0.0032	0.00064
Normality:	failed	p < 0.050								
Equal variance:	not tested									
Kruskal-Wallis with Dunn's										
0.01	1	1	1	1	1	1	1	1	1	p-value:
yes	no	no	no	no	no	no	no	no	no	Significance:

LCA 24h

50	25	10	5	2	0.4	0.16	0.08	0.016	0.0032	0.00064
Normality:	failed	p < 0.050								
Equal variance:	not tested									
Kruskal-Wallis with Dunn's										
<0.001	0.004	0.043	1	1	1	1	1	1	1	p-value:
yes	yes	yes	no	no	no	no	no	no	no	Significance:

GLCA 6h

50	25	10	5	2	0.4	0.16	0.08	0.016	0.0032	0.00064
Normality:	passed	p = 0.287								
Equal variance:	passed	p = 0.476								
One-Way ANOVA with Dunnett's test										
<0.001	0.187	0.999	0.985	0.894	0.95	1	0.949	1	0.999	p-value:
yes	no	no	no	no	no	no	no	no	no	Significance:

GLCA 24h

50	25	10	5	2	0.4	0.16	0.08	0.016	0.0032	0.00064
Normality:	failed	p < 0.050								
Equal variance:	not tested									
Kruskal-Wallis with Dunn's										
0.033	0.101	1	1	0.79	1	1	1	1	1	p-value:
yes	no	no	no	no	no	no	no	no	no	Significance:

TLCA 6h

50	25	10	5	2	0.4	0.16	0.08	0.016	0.0032	0.00064
Normality:	failed	p < 0.050								
Equal variance:	not tested									
Kruskal-Wallis with Dunn's										
1	1	0.367	1	1	1	1	0.533	1	1	p-value:
no	no	no	no	no	no	no	no	no	no	Significance:

TLCA 24h

50	25	10	5	2	0.4	0.16	0.08	0.016	0.0032	0.00064
Normality:	failed	p < 0.050								
Equal variance:	not tested									
Kruskal-Wallis										
				0.721						p-value:
no	no	no	no	no	no	no	no	no	no	Significance:

Figure S6

Figure S6 shows a table and a heatmap without statistic.

Figure S7A

Figure S7A shows showsplots with single point measurement without statistic.

Figure S7B

test: One-way ANOVA
groups: vs. control
parameters: α = 0.05
Normality: Shapiro-Wilk
Equal varian Brown-Forsythe

results:

RIPK3								
**control**	**CDCA 6h**	**CDCA 24h**	**control**	**TCDCA 6h**	**TCDCA 24h**	**control**	**GCDCA 6h**	**GCDCA 24h**
Normality:	passed	p = 0.079	Normality:	passed	p = 0.091	Normality:	passed	p = 0.065
Equal variance:	passed	p = 1.000	Equal variance:	passed	p = 0.126	Equal variance:	passed	p = 1.000
One-Way ANOVA with Dunnett's test			One-Way ANOVA with Dunnett's test			One-Way ANOVA		
p-value:	0.039	0.733	p-value:	0.081	<0.001	p-value:	0.803	
Significance:	yes	no	Significance:	no	yes	Significance:	no	

pRIPK3								
**control**	**CDCA 6h**	**CDCA 24h**	**control**	**TCDCA 6h**	**TCDCA 24h**	**control**	**GCDCA 6h**	**GCDCA 24h**
Normality:	passed	p = 0.543	Normality:	passed	p = 0.195	Normality:	passed	p = 0.422
Equal variance:	passed	p = 0.198	Equal variance:	failed	p < 0.001	Equal variance:	passed	p = 0.698
One-Way ANOVA			Kruskal-Wallis with Dunn's			One-Way ANOVA		
p-value:	0.422		p-value:	0.211	0.004	p-value:	0.865	
Significance:	no		Significance:	no	yes	Significance:	no	

Figure S7C

test: One-way ANOVA
groups: vs. control
parameters: α = 0.05
Normality: Shapiro-Wilk
Equal varian Brown-Forsythe

results:

RIPK3								
**control**	**LCA 6h**	**LCA 24h**	**control**	**TLCA 6h**	**TLCA 24h**	**control**	**GLCA 6h**	**GLCA 24h**
Normality:	passed	p = 0.445	Normality:	passed	p = 0.162	Normality:	passed	p = 0.158
Equal variance:	passed	p = 1.000	Equal variance:	passed	p = 0.238	Equal variance:	passed	p = 1.000
One-Way ANOVA with Dunnett's test			One-Way ANOVA with Dunnett's test			One-Way ANOVA with Dunnett's test		
p-value:	0.002	0.002	p-value:	0.231	0.013	p-value:	0.01	0.001
Significance:	yes	yes	Significance:	no	yes	Significance:	yes	yes

pRIPK3								
**control**	**LCA 6h**	**LCA 24h**	**control**	**TLCA 6h**	**TLCA 24h**	**control**	**GLCA 6h**	**GLCA 24h**
Normality:	passed	p = 0.064	Normality:	passed	p = 0.493	Normality:	passed	p = 0.236
Equal variance:	passed	p = 1.000	Equal variance:	passed	p 0 0.493	Equal variance:	passed	p = 1.000
One-Way ANOVA			One-Way ANOVA			One-Way ANOVA with Dunnett's test		
p-value:	0.053		p-value:	0.162		p-value:	<0.001	<0.001
Significance:	no		Significance:	no		Significance:	yes	yes

Figure S8

Figure S8 shows western blot images without statistical analysis.

Figure S9

test: One-way ANOVA
groups: vs. control
parameters: α = 0.05
Normality: Shapiro-Wilk
Equal variance: Brown-Forsythe

results:

pMLKL			
**control**	**CA 24h**	**TCA 24h**	**GCA 24h**
Normality:	passed	p = 0.068	
Equal variance:	passed	p = 0.053	
One-Way ANOVA with Dunnett's test			
p-value:	0.009	0.294	0.234
Significance:	yes	no	no

pMLKL			
**control**	**LCA 24 h**	**TLCA 24h**	**GLCA 24h**
Normality:	passed	p = 0.313	
Equal variance:	failed	p < 0.050	
Kruskal-Wallis with Dunn's test			
p-value:	0.101	0.041	0.379
Significance:	no	yes	no

pMLKL			
**control**	**UDCA 24h**	**TUDCA 24h**	**GUDCA 24h**
Normality:	failed	p < 0.050	
Equal variance:	not tested		
Kruskal-Wallis with Dunn's test			
p-value:	0.06	0.188	0.102
Significance:	no	no	no

pMLKL			
**control**	**CDCA 24h**	**TCDCA 24h**	**GCDCA 24h**
Normality:	failed	p < 0.050	
Equal variance:	not tested		
Kruskal-Wallis			
p-value:		0.148	
Significance:		no	

Figure S10

test: Chi-Square test with Bonferroni adjusted α-value
groups: indicated below
parameters: α = 0.05/9 conditions = 0.0056

results:

	p-value	
**native vs. CA**	not tested	
**native vs. RIPK3[Ser199Ala]**	not tested	**= control**
**CA vs. RIPK3[Ser199Ala]**	not tested	
**control vs. RIPK3[Ser199Asp][Ser227Asp]**	<0.001	yes
**control vs. RIPK3**	<0.001	yes
**control vs. RIPK3 + CA**	<0.001	yes
**RIPK3 + CA vs. RIPK3**	<0.001	yes
**RIPK3 + CA vs. RIPK3[Ser199Asp][Ser227Asp]**	<0.001	yes
**RIPK3 vs. RIPK3[Ser199Asp][Ser227Asp]**	<0.001	yes

Figure S11

test: One-way ANOVA
groups: vs. control
parameters: α = 0.05
Normality: Shapiro-Wilk
Equal variance: Brown-Forsythe

results:

IL-33		
**control**	**RIPK3**	**RIPK+CA**
Normality:	failed	p < 0.050
Equal variance:	not tested	
Kruskal-Wallis		
p-value:	0.436	
Significance:	no	

HMBG1		
**control**	**RIPK3**	**RIPK+CA**
Normality:	failed	p < 0.050
Equal variance:	not tested	
Kruskal-Wallis		
p-value:	0.731	
Significance:	no	

IL-6		
**control**	**RIPK3**	**RIPK+CA**
Normality:	passed	p = 0.208
Equal variance:	passed	p = 0.669
One-Way ANOVA		
p-value:	0.333	
Significance:	no	

Figure S12

test: One-way ANOVA
groups: vs. control
parameters: α = 0.05
Normality: Shapiro-Wilk
Equal variance: Brown-Forsythe

results:

control		Aniyomycine			RIPK3 + Aniyomycine		
		**15 min**	**30 min**	**24 h**	**15 min**	**30 min**	**24 h**
Normality:	passed (p = 0.97)						
Equal variance:	failed (p < 0.05)						
Kruskal-Wallis with Dunn test							
	p-value:	0.3	0.042	1	1	0.042	1
	Significance:	no	yes	no	no	yes	no

Figure S13

test: One-way ANOVA
groups: vs. control
parameters: α = 0.05
Normality: Shapiro-Wilk
Equal variance: Brown-Forsythe

results:

control	STS	RIPK3	RIPK3[S199A]	RIPK3[S227A]	RIPK3[S199/227A]	RIPK3[S199/227D]
Normality:	passed	p = 0,098				
Equal variance:	passed	p = 0,155				
One-Way ANOVA with Dunnett's test						
p-value:	<0,001	<0,001	<0,001	0,031	<0,001	0,003
Significance:	yes	yes	yes	yes	yes	yes

Figure S14

test: t-Test
groups: control vs. CA
parameters: α = 0.05
Normality: Shapiro-Wilk
Equal variance: Brown-Forsythe

results:

control	CA
Normality:	passed (p = 0.266)
Equal variance:	passed (p = 1.000)
Student's t-test:	p = 0.08
Significance:	no

Figure S15

Figure S15 shows western blot pictures without statistical analysis.

Figure S16

Figure S16 shows western blot pictures without statistical analysis.

Figure S17

Figure S17 shows western blot pictures without statistical analysis.

Table S1

Table S1 summarizes effects of RIPK3 knockout mice in liver disease models no statistical analysis was performed.

Table S2

In Table S2 contains demographic and group data characterizing the human hepatocytes, no statistical analysis was performed.

Table S3

In Table S3 contains demographic and group data characterizing the human hepatocytes, no statistical analysis was performed.

Table S4

test: t-Test
groups: reference vs. cholestasis
parameters: α = 0.05
Normality: Shapiro-Wilk
Equal varian Brown-Forsythe

results:

bilirubin	
**reference**	**cholestasis**
Normality:	failed (p < 0.050)
Equal variance:	not tested
Mann-Whitney Test:	p < 0.001
Significance:	yes

ASAT	
**reference**	**cholestasis**
Normality:	failed (p < 0.050)
Equal variance:	not tested
Mann-Whitney Test:	p = 0.043
Significance:	yes

ALAT	
**reference**	**cholestasis**
Normality:	failed (p < 0.050)
Equal variance:	not tested
Mann-Whitney Test:	p = 0.491
Significance:	no

albumin	
**reference**	**cholestasis**
Normality:	passed (p = 0.567)
Equal variance:	passed (p = 0.109)
Student's t-test:	p = 0.043
Significance:	yes

CRP	
**reference**	**cholestasis**
Normality:	failed (p < 0.050)
Equal variance:	not tested
Mann-Whitney Test:	p = 0.629
Significance:	no

Table S5

Table S5 contains primar sequences used in this study, no statistical analysis was performed.

Table S6

Table S6 contains detailed information on replica in Figure 1C, no statistical analysis was performed

Table S7

In Table S7 contain additional methylation sequencing data of the hepatocyte donor pool and non-parenchymal liver cells, no statistical analysis was performed.
