# Supplementary material for: RIPK3 promoter hypermethylation in hepatocytes protects from bile acid-induced inflammation and necroptosis
Source: Cell Death Dis. 2023 Apr 18;14(4):275. doi: 10.1038/s41419-023-05794-0 (PMC10113265; doi:10.1038/s41419-023-05794-0)
Supplement: Supplementary file 4 — Original Data File [file 41419_2023_5794_MOESM4_ESM.pdf]

Supplementary Material - 4

RIPK3 promoter hypermethylation in hepatocytes protects from bile acid-induced inflammation and necroptosis

Jessica Hoff[1,2], Ling Xiong[1,2], Tobias Kammann[1,2], Sophie Neugebauer[3], Julia M. Micheel[1,2], Nikolaus Gaßler[4], Michael Bauer[1,2], Adrian T. Press[1,2,5]

1 Department of Anesthesiology and Intensive Care Medicine, Nanophysiology Group, Jena University Hospital, Jena 07747, Germany
2 Center for Sepsis Control and Care, Jena University Hospital, Jena 07743, Germany
3 Department of Clinical Chemistry and Laboratory Diagnostics, Jena University Hospital, Jena 07747, Germany
4 Pathology, Jena University Hospital, Jena 07747, Germany
5 Faculty of Medicine, Friedrich Schiller University Jena, Jena 07747, Germany

Correspondence

Adrian Press,
Am Klinikum 1, 07747 Jena
+49 3641/ 9 323139
Adrian.Press@med.uni-jena.de

Keywords

necroptosis, hepatocytes, RIPK3, bile acids, methylation, inflammation

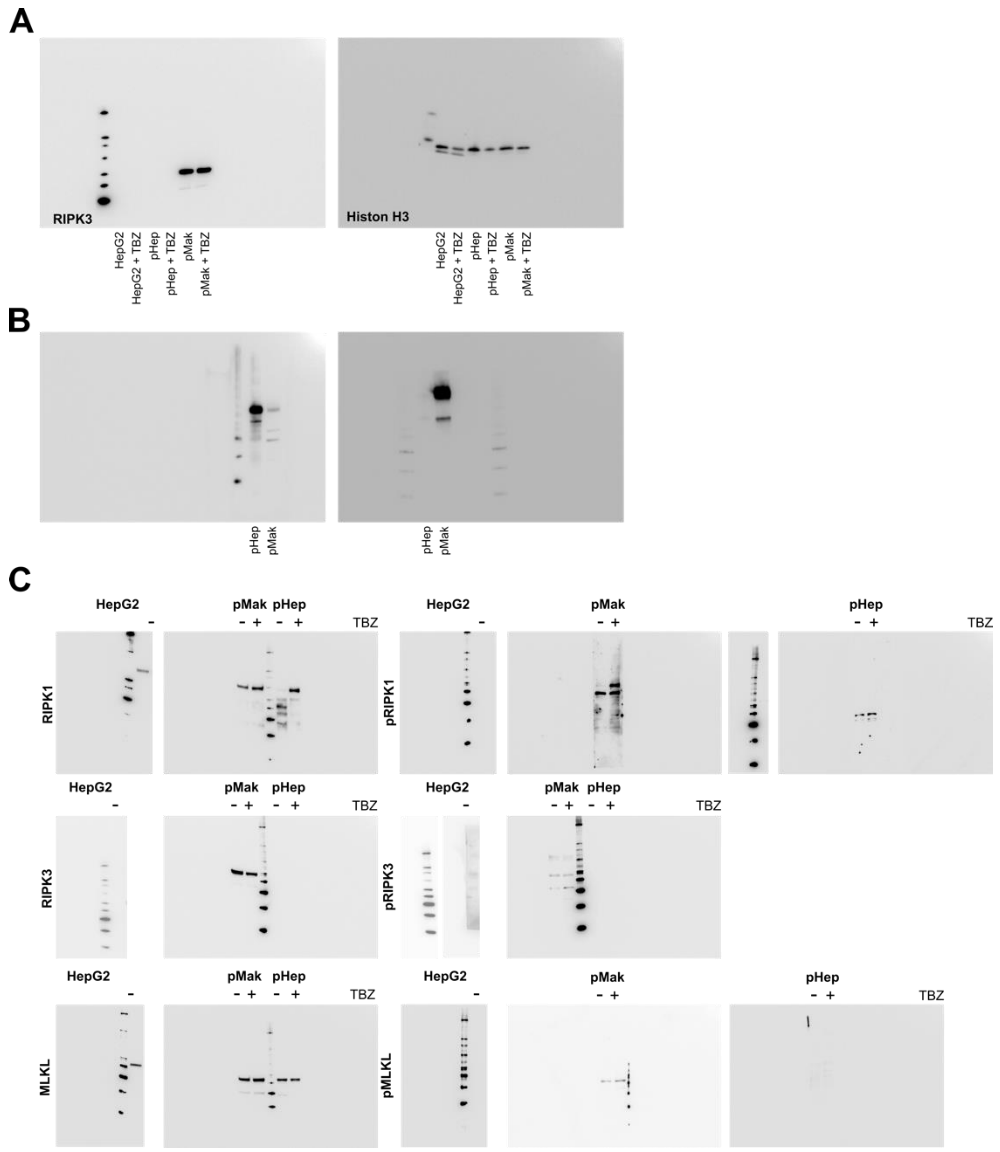

Supplementary Figure S15: Western blot raw data files for main Figure 1.

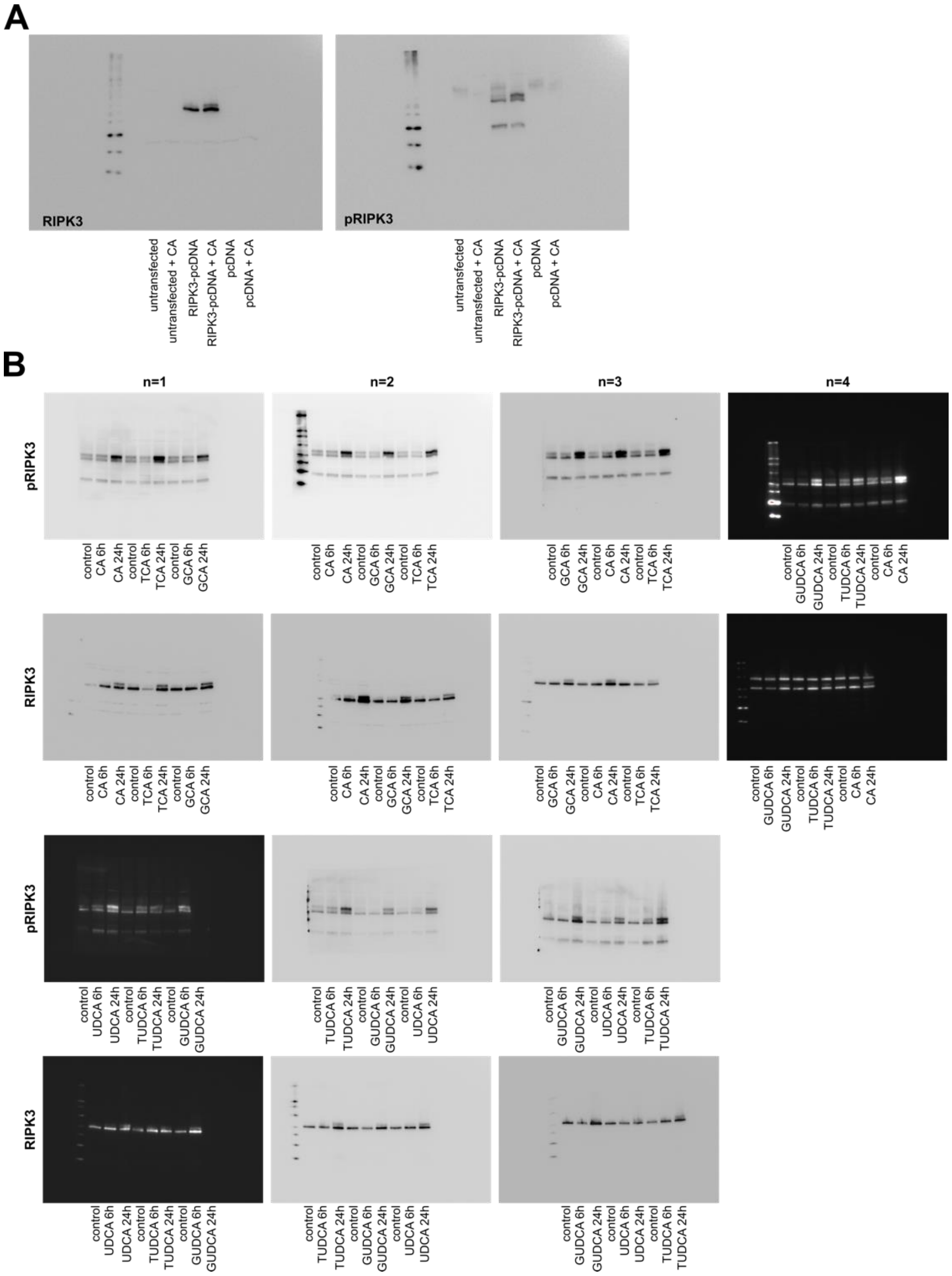

Supplementary Figure S16: Western blot raw data files for main Figure 3.

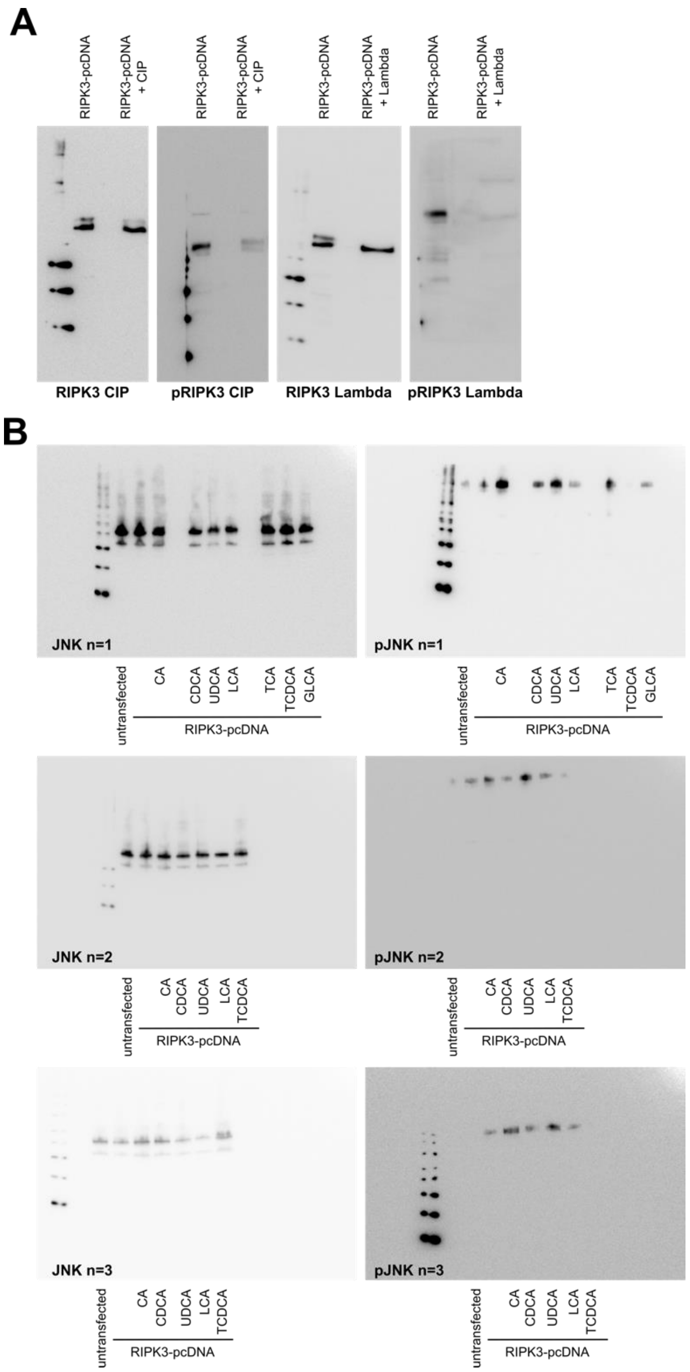

Supplementary Figure S17: Western blot raw data files for main Figure 4.
